# Reduction in mobility and COVID-19 transmission

**DOI:** 10.1038/s41467-021-21358-2

**Published:** 2021-02-17

**Authors:** Pierre Nouvellet, Sangeeta Bhatia, Anne Cori, Kylie E. C. Ainslie, Marc Baguelin, Samir Bhatt, Adhiratha Boonyasiri, Nicholas F. Brazeau, Lorenzo Cattarino, Laura V. Cooper, Helen Coupland, Zulma M. Cucunuba, Gina Cuomo-Dannenburg, Amy Dighe, Bimandra A. Djaafara, Ilaria Dorigatti, Oliver D. Eales, Sabine L. van Elsland, Fabricia F. Nascimento, Richard G. FitzJohn, Katy A. M. Gaythorpe, Lily Geidelberg, William D. Green, Arran Hamlet, Katharina Hauck, Wes Hinsley, Natsuko Imai, Benjamin Jeffrey, Edward Knock, Daniel J. Laydon, John A. Lees, Tara Mangal, Thomas A. Mellan, Gemma Nedjati-Gilani, Kris V. Parag, Margarita Pons-Salort, Manon Ragonnet-Cronin, Steven Riley, H. Juliette T. Unwin, Robert Verity, Michaela A. C. Vollmer, Erik Volz, Patrick G. T. Walker, Caroline E. Walters, Haowei Wang, Oliver J. Watson, Charles Whittaker, Lilith K. Whittles, Xiaoyue Xi, Neil M. Ferguson, Christl A. Donnelly

**Affiliations:** 1 MRC Centre for Global Infectious Disease Analysis, J-IDEA, Department of Infectious Disease Epidemiology, Imperial College London, St Mary’s Campus, London, UK; 2 School of Life Sciences, University of Sussex, Brighton, UK; 3 Department of Statistics, University of Oxford, Oxford, UK

**Keywords:** Computational models, SARS-CoV-2, Viral infection, Epidemiology

## Abstract

In response to the COVID-19 pandemic, countries have sought to control SARS-CoV-2 transmission by restricting population movement through social distancing interventions, thus reducing the number of contacts. Mobility data represent an important proxy measure of social distancing, and here, we characterise the relationship between transmission and mobility for 52 countries around the world. Transmission significantly decreased with the initial reduction in mobility in 73% of the countries analysed, but we found evidence of decoupling of transmission and mobility following the relaxation of strict control measures for 80% of countries. For the majority of countries, mobility explained a substantial proportion of the variation in transmissibility (median adjusted R-squared: 48%, interquartile range - IQR - across countries [27–77%]). Where a change in the relationship occurred, predictive ability decreased after the relaxation; from a median adjusted R-squared of 74% (IQR across countries [49–91%]) pre-relaxation, to a median adjusted R-squared of 30% (IQR across countries [12–48%]) post-relaxation. In countries with a clear relationship between mobility and transmission both before and after strict control measures were relaxed, mobility was associated with lower transmission rates after control measures were relaxed indicating that the beneficial effects of ongoing social distancing behaviours were substantial.

## Abstract (web summary)

Social distancing policies aiming to reduce COVID-19 transmission have been reflected in reductions in human mobility. Here, the authors show that reduced mobility is correlated with decreased transmission, but that this relationship weakened over time as social distancing measures were relaxed.

## Introduction

Since the declaration of COVID-19 as a Public Health Emergency of International Concern in late January 2020^1^, many countries have struggled to prevent the importation^2,3^ and subsequent local transmission of SARS-CoV-2^4^, the virus that causes COVID-19^5^. While sustained transmission has continued globally, some countries are now entering their 2^nd^ or 3^rd^ wave of the epidemic^6^.

Social-distancing, case isolation, and shielding have been widely used to limit community-level transmission of SARS-CoV-2 and protect vulnerable groups^7,8^. These interventions aim to reduce mobility and contacts within the population and thus to reduce the transmission of SARS-CoV-2, as measured by the effective reproduction number (R, the average number of secondary cases caused by a primary case). Early in the epidemic, human mobility reductions as recorded in a variety of digital data sources were shown to correlate well with decreases in COVID-19 incidence^9–11^ and social contacts^12^.

In the face of the threats posed by COVID-19, most countries rapidly implemented strict social distancing policies to suppress transmission (bringing R below 1) and thus avoid overwhelming healthcare capacity^13^. While a convincing reduction in case incidence was observed, at least temporarily, in many countries^14–18^, and in some cases a reduction in transmission has been explicitly linked to the reduction in mobility^19,20^, many countries are still experiencing continued or resurgence of widespread transmission of SARS-CoV-2^21,22^.

Understanding how well mobility data reflects population contact rates and whether that relationship is changing in countries that are transitioning, exiting or re-entering lockdown measures is important for tracking the trajectory of national epidemics and assessing the effectiveness of ongoing control measures. Here, we develop a framework to infer the relationship between mobility and the key measure of population-level transmission, the effective reproduction number. The framework is applied to 52 countries with sustained SARS-CoV-2 transmission based on two distinct country-specific automated measures of human mobility, Apple and Google mobility data.

## Results

### Temporal variation in mobility

**Fig. 1:**
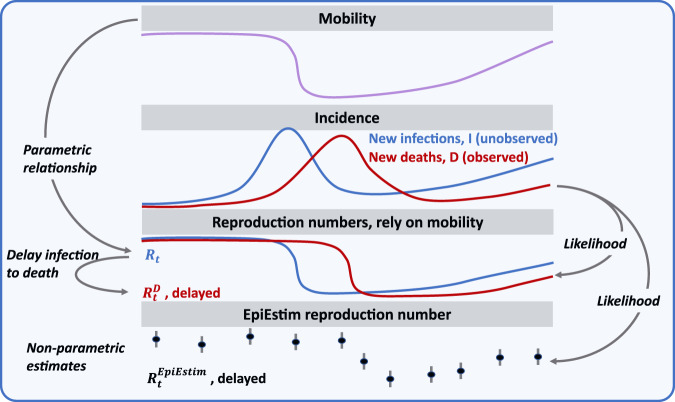
Schematic of the methodology. A parametric relationship between transmission and mobility is assumed and allows to link the effective reproduction number at time of infection (*R*_*t*,*i*_) to mobility (*m*_*t*,*i*_).We obtain the delayed effective reproduction number at time of death ($R_{t,i}^D$) as a weighted average of *R*_*t*,*i*_’s, relying on the delay between infection and death (see Methods section). Inference relies on the likelihood of observed vs predicted deaths, with predicted deaths being a function the $R_{t,i}^D$ (see Methods section). To estimates how much variations in transmission can be explained by variations in mobility, we estimate a non-parametric and delayed reproduction number relying on EpiEstim framework^23^ ($R_{t,i}^{D,EpiEstim}$) and compare it to $R_{t,i}^D$.

**Fig. 2:**
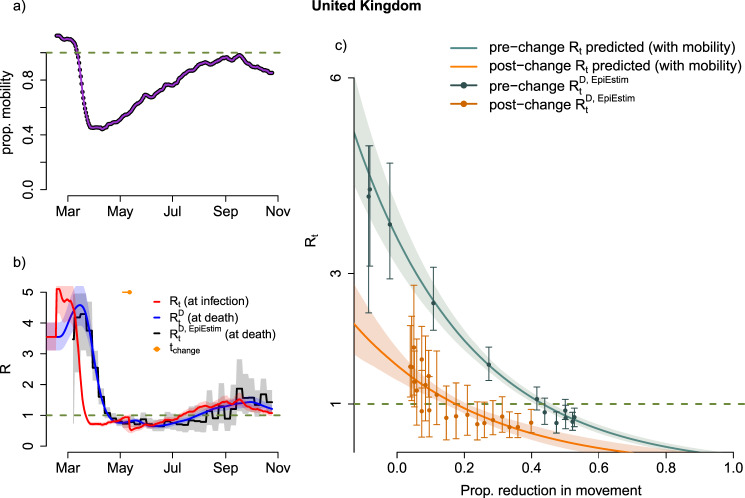
Relationship between human mobility and transmission. **a** Smoothed combined Apple-Google mobility. **b** Estimated daily effective reproduction number for infections (red, *R*_*t*,*i*_) and delayed effective reproduction at time of deaths (blue, $R_{t,i}^D$) estimated using the best-fitting model and mobility data. Effective reproduction number ($R_{t,i}^{D,EpiEstim}$) estimated from deaths data alone using a daily 7-day non-overlapping window (black). In each case shading represents the 95% credible interval. Horizontal orange dot and line show the median and 95% CrI for the timing of the change in the relationship between mobility and transmission. **c** Estimates of the reproduction number against changes in mobility using our best model (5 estimated parameters): green/red lines show the median predictions pre/post change in relationship, with shading indicating the 95% CrI. The ‘EpiEstim’ effective reproduction number using *‘*EpiEstim’-like method are shown as error bars in green /orange for approximate pre-post change in relationship with 95% CrI (bands). Results based on the Apple-Google mobility data-stream – equivalent figure using the Apple transit mobility data-stream can be found in the SI (Supplementary Fig. 5); the Apple-related figure shows a qualitatively similar fit but with a marginally better DIC (DIC reduction of 32). Equivalent figures for other countries can be found in the SI (Supplementary Figs. 4–5).

Smoothed daily estimates representing a measure of mobility relative to the highest country-specific estimate in the pre-pandemic range were obtained (see Methods section). We found a consistent pattern of a sharp reduction followed by a gradual recovery in mobility across countries and in multiple mobility data sources (Figs. 1 and 2a, for a schematic and the UK and SI for other countries).

Our analysis is based on 52 countries for which both epidemiological and mobility data were available, and which met our active transmission thresholds (see Methods section). This included 36 countries for which we had both Google and Apple mobility data and 16 countries for which we had only Google mobility data (see Supplementary Fig. 2). Apple mobility data included three data-streams: “driving”, “transit” and “walking” mobility; while Google mobility data included six data-streams: “grocery and pharmacy”, “parks”, “residential”, “retail and recreation”, “transit stations” and “workplaces”. In addition, we combined all Apple data-streams to obtain an Apple mobility measure. Similarly, we obtained a Google mobility data-stream by combining all Google specific data-streams (excluding “parks” and “residential” for Google, see Methods section and Supplementary Figs. 2–3). Finally, a combined Apple-Google mobility was also defined as a combination of both the Apple and Google mobility (see Methods section). While all analyses were performed independently for each data-stream, unless otherwise stated we present results using the combined Apple-Google mobility measure.

The median mobility across the 52 countries reached its minimum on the 11th of March 2020, with a reduction of 63% from baseline. Mobility then recovered, with an estimated median reduction in mobility on the 25th of October 2020 reaching 14% from baseline. We observed substantial variations in mobility patterns across countries (see SI) with the interquartile range (IQR) of the reduction in mobility on the 11th of March and 25th of October 2020 reaching [51; 67]% and [7; 22]% respectively (Supplementary Figs. 1–2).

The 10 countries with the smallest changes saw mobility reduction within a range of 37 to 51% from baseline (smallest to largest changes observed in Moldova, Afghanistan, Switzerland, Ecuador, Paraguay, Sweden, Ukraine, Panama, Dominican Republic and Denmark). The 10 countries with the largest changes saw mobility reduction within a range of 72 to 83% from baseline (smallest to largest changes observed in Honduras, Poland, Costa Rica, Italy, Guatemala, Peru, Philippines, Argentina, France, Bolivia).

### Correlation between mobility and transmissibility

We evaluated the correlation between mobility and transmissibility by fitting two models to the country-specific time-series of COVID-19 deaths. Both models were derived from the renewal equation^23,24^, where daily reported deaths are linked to deaths in the past and the level of transmissibility characterised by a time-varying reproduction number (see Methods section).

The main model assumes that the time-varying effective reproduction number is a function of the basic reproduction number, *R*_0,*i*_, and mobility following:

1 $$log( {R_{t,i}} ) = log( {R_{0,i}} ) - \beta _i( {1 - m_{t,i}} )$$

where *m*_*t*,*i*_ represents the mobility in one of the 52 countries, *β*_*i*_ characterise how transmission is linked to mobility. As we fit the models to reported deaths, we then related the effective reproduction number at time of infection, *R*_*t*,*i*_, to the delayed effective reproduction number at time of deaths, $R_{t,i}^D$, via the delay between infection and deaths (see Figs. 1–2 and Methods section).

The alternative model assumed that the relationship between transmission and mobility changed over time, with two successive values of *R*_0,*i*_’s and of *β*_*i*_’s estimated alongside the time of the change in relationship. The best model was chosen based on a difference in DIC (Deviance Information Criterion)^25^ greater than 10, reflecting a substantial improvement in fit.

To evaluate the model fit, relying on the time-series of deaths only, we also estimated an ‘EpiEstim’ time-varying effective reproduction number: $R_{t,i}^{D,EpiEstim}$ using the ‘EpiEstim’ framework from^23^ (see Methods section). Both $R_{t,i}^D$ and $R_{t,i}^{D,EpiEstim}$ are comparable as they reflect a delayed transmission level, i.e., transmission level at time of death rather than at time of infection. While the delayed $R_{t,i}^D$ (and $R_{t,i}$ reflecting transmission level at infection) can be viewed as parametric estimates as they rely explicitly on mobility, the delayed $R_{t,i}^{D,EpiEstim}$ can be viewed as non-parametric estimates as they rely solely on the observed pattern of deaths. As such, estimating $R_{t,i}^{D,EpiEstim}$ (i) imply inferring more parameters (i.e., one per week), (ii) is likely to better track changes in transmission, but (iii) does not give an explicit understanding of the drivers of transmission. Therefore, by comparing the parametric and non-parametric estimates ($R_{t,i}^D$ and $R_{t,i}^{D,EpiEstim}$respectively), we can quantify how much of variations in transmission can be explained by variations in mobility (see Fig. 1, for a schematic of the methods).

All models assumed a negative binomial distribution of deaths to account for over-dispersion in the data (see Methods section and SI).

For the UK (as well as other countries, see SI.2-5), a sharp decline in mobility (Fig. 2a) was correlated with a sharp decline in the estimated effective reproduction number for infections $R_{t,i}$ (Fig. 2b, red), which, after accounting for the infection-to-death delay, is later reflected in a sharp decline in the estimated reproduction number for deaths $R_{t,i}^D$ (Fig. 2b, blue). The temporal trends in $R_{t,i}^D$ are well correlated to those in the ‘observed’ effective reproduction number for deaths, $R_{t,i}^{D,EpiEstim}$, as estimated by the ‘EpiEstim’-like method (Fig. 2b, grey).

From around the second half of May 2020; we see a substantial change in the relationship between mobility and transmissibility, with a gradual decoupling or dampening of the relationship. As mobility gradually increased, the transmissibility still increased but more slowly than expected from the previously inferred relationship.

**Fig. 3:**
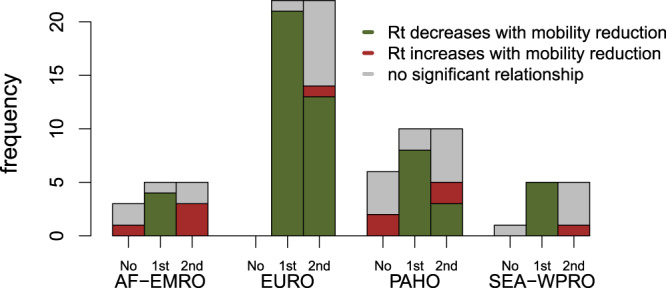
Summary of relationship between mobility and transmissibility. Frequency of countries for which we found a significantly decreasing, increasing, or no clear relationship between mobility and transmissibility (green, red and grey bars respectively). For each WHO region, we further divided countries for which no change in relationship was inferred (labelled “No”), and for those where a change in relationship was significant, we plot separately the nature of the relationship during the first and second time periods (labelled “1st” and “2nd” respectively). Some WHO regions were aggregated, with AF-EMRO corresponding to the African and Eastern Mediterranean Regions, EURO corresponding to the European Region, PAHO corresponding to the Pan-American Region, and SEA-WPRO corresponding to the South-East Asia and the Western Pacific Regions.

Similarly to the UK, the majority of countries saw a substantial change in the mobility-transmissibility relationship (42 out of 52 countries, see Fig. 3 and Supplementary Fig. 4). For each country and period (where relevant, two periods were considered), we determined whether a decrease in mobility was significantly associated with decreased transmissibility (95% CrI of the parameter *β*_*i*_ not including 0). Overall, for 57% of country-periods, the relationship was as expected and qualitatively similar to the relationship observed in the UK. This increased to 90% of countries when considering only the 1st period prior to the change in the relationship. Overall, for the remaining 43% of country-periods, the relationship was either non-significant (32%) or reversed (11%).

By linking mobility to transmissibility, we were able to capture both the temporal trends in transmissibility and its relationship with mobility across multiple countries (Figs. 2b, 2c and Supplementary Fig. 4). In the UK, the relationship between mobility and transmissibility (Fig. 2c) is well captured by our model with 98% and 69% the variation in $R_{t,i}^{D,EpiEstim}$ explained by the model pre and post the change in the relationship, respectively (adjusted R-squared of $R_{t,i}^{D,EpiEstim}$ against $R_{t,i}^D$). Such predictive ability varied substantially across country-periods (Table SI.1), with an estimated median adjusted R-squared of 48% across country-periods (IQR across country-periods [27–77]%). The predictive ability increased when a change in the relationship was inferred, with median adjusted R-squared reaching 74% (95 percentile [49–91]%) and 30% (95 percentile [12–48]%) pre- and post-change in the relationship. Finally, a further increase was observed when restricting to the first period and having experienced a large epidemic (more than 5000 cumulative deaths), adjusted R-squared: 81% (95 percentile [68–92]%).

### Mobility thresholds

**Fig. 4:**
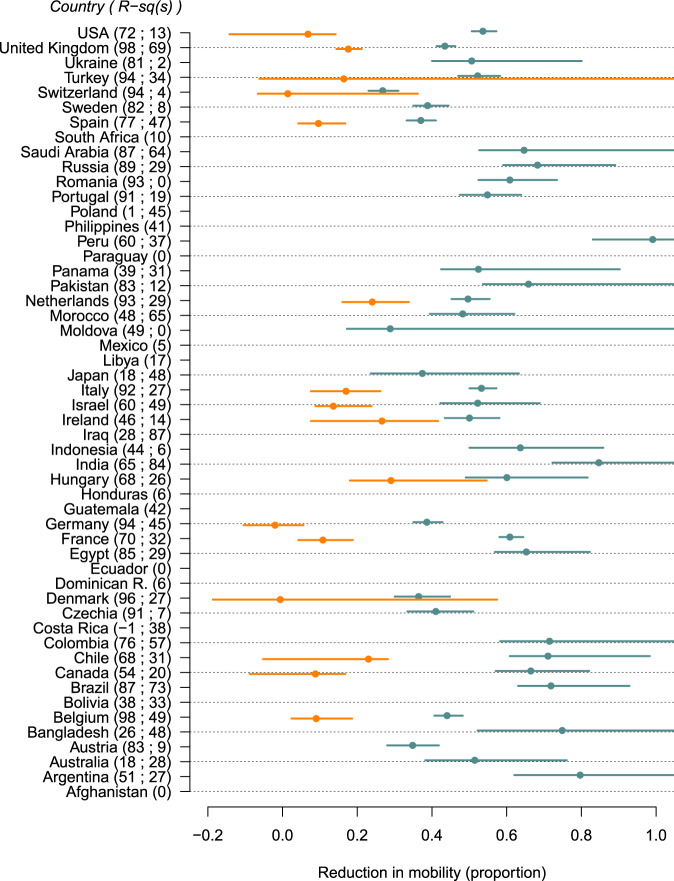
Country-specific mobility thresholds to interrupt transmission. Interruption of transmission occurs when R < 1, thresholds presented are based on the Apple-Google mobility data-stream. Thresholds are only defined when transmissibility significantly decreases with a reduction in mobility. Countries with no threshold are still shown. Points indicate the median and horizontal bars the 95% CrI. Upper 95%CrI limits of the mobility going above 1 indicate that the upper limits remain unidentifiable. Turquoise and orange thresholds correspond to the pre- and post-change in the mobility-transmissibility relationship respectively. The y-axis shows specific countries and, next to their names, the predictive abilities of the model in that country (i.e., adjusted R-squared of $R_{t,i}^{D,obs}$ against $R_{t,i}^D$; two R-squared values indicate a change in relationship was inferred).

Where possible, we estimated mobility thresholds defined as the reduction in mobility necessary to bring the reproduction number below the critical threshold of 1 (Fig. 4).

We estimated that in the UK, initially a reduction of 43% (95% CrI: 41–46%) of Apple-Google mobility would be necessary to reduce the reproduction number below 1. After the ‘dampening’ of the relationship due to other social distancing behaviours, a lower reduction of 18% (95% CrI: 14–21%) would be sufficient to reduce the reproduction number below 1.

Given these thresholds, and based on an estimated mobility reduction in the UK of 15% as of October 25^th^, 2020, we predict that the epidemic in the UK was not under control as of October 25th, 2020 (Fig. 2b, *R*_*t*,*i*_ in red above 1) although this is uncertain given that 15% is within the 95% credible interval of our estimated threshold post change.

On October 25th in the UK, we estimate that the reproduction number for new infections, *R*_*t*,*i*_, was 1.07 (95% CrI: 0.99–1.17). Furthermore, the reproduction numbers estimated from deaths, $R_{t,i}^D$, was estimated at 1.21 (95% CrI: 1.10–1.33) reflecting higher past mobility which had recently started to decrease (Fig. 2a).

We found substantial heterogeneity between countries in estimating this mobility threshold (Fig. 4). The median mobility reduction threshold across the countries considered (estimated as the median of country-specific medians, where estimated) decreased from 51% to 16% after the change in the mobility-transmissibility relationship corresponding to the relaxation of strict control measures. This reflected the dampening of the relationship between mobility and transmissibility, therefore suggesting that recently, less drastic reductions in mobility were necessary to achieve control.

As of October 25th, a mobility threshold could be clearly defined for 16 countries (95%CrI of the most recent parameter *β*_*i*_ not including 0). Among those, the observed reduction in mobility was greater than the estimated upper 95% CrI thresholds in one country (Canada), indicating an epidemic under control (Fig. 3 and Supplementary Fig. 4, and Supplementary Table 1). In three countries (Germany, Hungary and Turkey), the reductions in mobility were lower than the estimated lower 95% CrI thresholds, indicating ongoing epidemics. For the remaining 12 countries (Belgium, Chile, Denmark, France, Ireland, Israel, Italy, Netherlands, Spain, Switzerland, UK, USA), the latest mobility estimates overlapped with the latest mobility thresholds estimated, indicating uncertainty in the level of transmission control.

The mobility threshold estimates were robust to assumptions about the serial interval distribution (Supplementary Table 1). In addition (Supplementary Figs. 6–7), across countries, the estimated mobility thresholds were not correlated with the estimated basic reproduction numbers and estimated parameters were robust to excluding the very early dynamic from the likelihood (i.e., excluding the period before 100 cumulative deaths were reported).

### Data-streams used to parameterise the mobility-transmissibility relationship

The results presented above relate to the combined Apple-Google data-stream. However, our model was also fitted independently for each of the 11 other data-streams defined (i.e., 3 Apple data-streams, the combined Apple data-stream, 6 Google data-streams, and the combined Google data-stream).

**Fig. 5:**
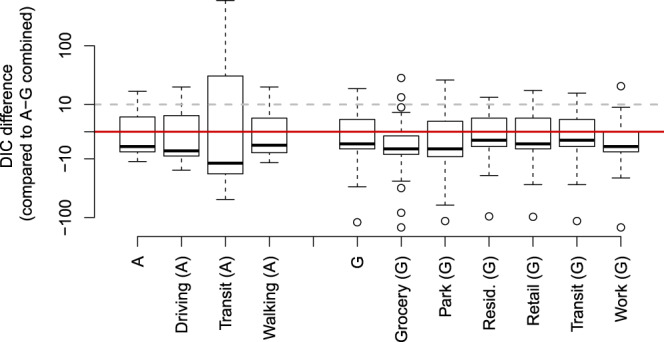
Comparison of fit between the individual data-streams and the Apple-Google data-stream used in the main results. Fit is assessed as the difference in DIC, a positive value (above the red horizontal line) indicates the individual data-stream could be favoured over the Apple-Google data-stream. The boxplot shows medians, interquartile ranges, ranges and outliers. Above the threshold of 10 highlighted with the dashed grey line, the individual data-stream would have substantially improved the fit of the model. The y-axis is on a “signed” square root scale.

Generally, the Apple-Google data-stream performed better than the other mobility data-streams (Fig. 5). Out of the 52 countries considered, in 8 countries, one or more individual data-streams performed better (DIC differences above 10). In six instances the “Transit” data-stream from Apple performed best (Brazil, France, Mexico, Philippines, UK and USA), while in the other two instances, the ‘Walking’ and ‘Driving’ data-streams from Apple performed best (Italy and Spain respectively). For those 8 countries, while the fit was quantitatively improved (i.e., substantial decrease in DIC), qualitatively, it did not dramatically change the relationship observed with the combined Apple-Google data-stream (see Supplementary Fig. 5). However, using the ‘Transit’ data-stream in Mexico made a change in the mobility-transmissibility relationship becoming identifiable, with the expected dampening of the relationship becoming apparent.

## Discussion

We found consistent evidence that automated measures of mobility correlate well with transmission intensity of SARS-CoV-2 over time in several countries. The relationship holds for 12 mobility data-streams based on Apple and Google mobility data and was robust to assumptions about the likelihood and serial interval distribution. We found strong evidence that the relationship between mobility and transmissibility changed over time, typically, a dampening indicating that smaller reductions in mobility can result in epidemic control likely due to other social distancing behaviours. As mobility data increasingly become available in real-time (currently updated across countries with 2-to-4 and 7-to-10-days delay for Apple and Google mobility data, respectively), future epidemiological analysis may increasingly rely on this type of data.

We conclude that for 52 countries having experienced, or still experiencing, substantial active SARS-CoV-2 transmission, there was a strong link between mobility measures and transmissibility, supporting the implementation of population-wide social distancing interventions to control the epidemic. Encouragingly, in the majority of countries, we found clear evidence of a recent dampening of the relationship between transmission and mobility, suggesting alternative control strategies have been successfully implemented and significantly decreased transmission. This, however, was insufficient to prevent a second wave of infection in many countries as mobility, and thus contact rates, gradually increased.

Mobility measures seem to reflect the level of contact and therefore level of transmission well. As such measures could be made available in real-time, mobility data could become an important aspect of forecasting efforts. An earlier version (May 2020) of this analysis explored such aspects^26^, but the change in relationship demonstrated here means that more needs to be understood about additional drivers of transmission before reliable forecasting based on mobility can be achieved.

Our framework allows us to estimate country-specific mobility thresholds to control transmission: if the reduction in mobility reaches a certain level, we predict that SARS-CoV-2 infection incidence will decline, if all other factors that impact on transmissibility stay unchanged. Although individual and combined Apple and Google mobility measures differ, and therefore so do the mobility threshold estimates, the link between transmissibility and each mobility measure was clear.

The heterogeneity in estimated mobility thresholds between countries likely reflects socio-cultural differences and/or the differences in the interventions each country has implemented. While we were able to characterise between-country heterogeneity, within-country heterogeneity is likely to also exist but were not considered here, for country-specific analyses of transmission see: for Brazil^21^, Italy^20^, and the USA^19^.

Previous studies, prior to the pandemic, have shown how the proximity and interpersonal distances maintained between people while interacting vary substantially between countries, likely due to cultural differences^27^, and this could influence baseline national levels of SARS-CoV-2 transmission. Similarly, it is likely that awareness of SARS-CoV-2 transmission will affect those interpersonal distances differently between countries, leading to heterogeneities in the relative reductions in mobility required to achieve COVID-19 control.

In addition, the COVID-19 public health responses are highly variable between countries. In particular, the levels of contact-tracing and testing vary considerably. South Korea, having previously experienced a large MERS coronavirus outbreak^28^, implemented an aggressive strategy of tracing (and testing) early on^29^, allowing rapid control of the epidemic. South Korea was not included in this analysis as reported deaths remained relatively low, falling short of our threshold for inclusion.

It follows that country-specific mobility thresholds are likely not constant but can vary over space and time. As a country intensifies its contact-tracing efforts, the mobility threshold would likely decrease (i.e., a smaller reduction would be required). Our initial analysis indicated that in the few countries where population-wide social distancing and case isolation had been successfully implemented, the margin to lift mobility restrictions was very small if everything else remains the same^26^. However, as social distancing behaviours remained and alternative strategies such as more complete contact-tracing^30^ were implemented, we demonstrated a dampening of the mobility-transmissibility relationship, which meant the relaxation of mobility restrictions could be more substantial without risking the success achieved.

As many countries are re-imposing social-distancing policies, our analysis illustrates that sustainable relaxation of population-wide social-distancing measures should be undertaken very carefully and replaced with equally effective control measures, such as thorough contact-tracing^30,31^. If further waves of infection are to be avoided, more stringent control measures should be rapidly implemented. While the encouraging early vaccine trial results offers a glimpse of hope, with vaccines likely to play a significant role in controlling SARS-CoV-2; the global scale of the pandemic means that alternative control strategies should remain a priority until large-scale vaccine production and delivery is feasible.

## Methods

### Data

Data on deaths due to COVID-19 by country were sourced from the WHO COVID dashboard^6^ and the European Centres for Disease Control (ECDC)^4^, including daily death counts reported by each country’s official surveillance system up to October 25th, 2020. Our analysis is based on countries for which at least 70 deaths were observed weekly for at least 4 consecutive weeks. This criterion was chosen to ensure that the countries included showed evidence of substantial active transmission.

Mobility data were sourced from Apple^32^ and Google^33^. These data reflect the movement of people with an Apple or Android device using mapping apps. For the Apple data, the measure of mobility is reported for three data-streams: “driving”, “transit” and “walking” mobility. For the Google data, the measure of mobility is reported for six data-streams: “grocery and pharmacy”, “parks”, “residential”, “retail and recreation”, “transit stations” and “workplaces”. All measures estimate relative daily mobility for each country and are quantified relative to the maximum mobility measured prior to the pandemic WHO declaration. Apple and Google mobility data were available from January 13th and February 15th, 2020, respectively, up to the last day that deaths were analysed (October 25th).

Our analysis is based on 52 countries for which we had epidemiological data (meeting our active transmission thresholds) and some mobility data. This included 36 countries for which we had both Google and Apple mobility data; and 16 countries for which we had only Google mobility data (see Table S1).

### Processing mobility data

The various mobility data-streams (i.e., driving, walking and transit movement for Apple and the six data-streams for Google) showed both short- and long-term variability in movement levels. For each data-stream, we smoothed weekly patterns by using the 7-days rolling averaged mobility. When mobility measures were missing, linear interpolation of the 7-days rolling averaged mobility were used (see Supplementary Fig. 2). Each processed data-stream was used independently in the analysis. In addition, we defined 3 additional data-streams: the “Apple” data-stream as the mean across all three Apple data-streams; the “Google” data-stream as the mean across “grocery and pharmacy”, “retail and recreation”, “transit stations” and “workplaces” (excluding “parks” and “residential” on the basis of inconsistent trends, see Supplementary Fig. 2); the “Apple-Google” data-stream as the mean across the newly defined “Apple” and “Google” data-streams defined above. We therefore obtain a single daily measure of relative mobility by country *m*_*t*,*i*_ for up to 12 data-streams (Fig. 1a).

### Estimating transmissibility, R, using mobility data

We define the effective reproduction number on day *t*, *R*_*t*,*i*_, which reflects the level of transmissibility in country *i* on that day. We assume *R*_*t*,*i*_ is linked to relative mobility on that day via Eq. (1) (see above), where *R*_0,*i*_ is the basic reproduction number in country *i* and *m*_*t,i*_ is the relative mobility in country *i* on day *t*. When mobility is at its peak (100%), transmissibility is characterised by the basic reproduction number. Reduced mobility leads to reductions in the effective reproduction number (when *β*_*i*_ is positive). As the smoothed mobility is relative to the maximum observed during the pre-pandemic period, the estimates of the basic reproduction numbers can be thought of as pre-pandemic, similarly to defining the reproduction number of a vector-borne disease as transmissibility during a period with a given vectorial transmission.

In this framework, due to the delay between infection and deaths, the effective reproduction number experienced by those dying on day *t* in country *i*, $R_{t,i}^D$, is a weighted average of the effective reproduction number on day *t*, *R*_*t*,*i*_:

2 $$R_{t,i}^D = \mathop {\sum}\limits_{s = 0}^t {R_{s,i}h\left( {t - s} \right)}$$

assuming that the infection-to-death interval follows a gamma distribution, *h*, with mean 18.8 days and standard deviation of 8.46 days^16^ (see SI for details).

We relate the observed reported deaths on day *t* in country *i* to the basic reproduction number (*R*_0,*i*_) and the parameter (*β*_*i*_) linking transmissibility to mobility (*m*_*t*,*i*_) using the renewal equation^23,24^:

3 $$D_{t,i}\sim NB\left( {R_{t,i}^D\mathop {\sum}\limits_{s = 0}^t {\left[ {D_{s,i}w_{t - s}} \right],\;\delta } } \right)$$

where *D*_*t*,*i*_ is the reported deaths on day *t* in country *i*, and *w* is the serial interval (i.e., a serial interval for deaths defined as the time between deaths of the infector and infectee) assumed to be gamma distributed with mean of 6.48 days and standard deviation 3.83 days^13^. Here we assume that the number of reported deaths follow a negative binomial distribution (such that the variance in the observed numbers of deaths is greater than or equal to the expected number of deaths) with over-dispersion *δ*.

As the framework outlined above is an extension of the framework developed in^23^, estimates of transmissibility obtained are robust to under-reporting of deaths but are affected by variation in levels of reporting. This justifies our choice of estimating transmissibility based on reported deaths (more likely to be reported at a consistent rate) rather than reported cases (which vary dramatically with testing capacity). This choice makes the analysis more challenging as we must account for variations in mobility that instantaneously affect transmissibility, which is reflected later when deaths are observed.

Once the relationship between mobility and *R*_*t*,*i*_ is estimated, we can evaluate the distribution of *R*_*t*,*i*_ for any level of mobility. Using a fine grid of mobility, we obtained estimates of corresponding *R*_*t*,*i*_, and this allowed us to estimate the distribution of the reduction of mobility when *R*_*t*,*i*_ = 1. This mobility threshold can be interpreted as the reduction in measured mobility that would be necessary in order to achieve control (R < 1), given the other behaviours of the population over the period under study (e.g., country-specific ways that people are interacting with each other and any country-specific additional control measures such as testing and contact tracing).

### Change in the relationship between mobility and transmission

As countries continue to seek a way to ease social distancing measures, alternative public health control strategies are being considered and implemented, such as increased testing and contact tracing. Furthermore, while restrictions on travel are being relaxed, often recommendations for social distancing remain in force. We would therefore expect some decoupling of transmission and mobility, leading to a weakening of the correlation between mobility and underlying contact rates (and therefore transmission). The effect of ongoing effective controls which are decoupled from mobility would translate into a reduction of *R*_0,*i*_, resulting in a reinterpretation of *R*_0,*i*_, (and possibly a change in *β*_*i*_), i.e., if the virus had been originally introduced while those measures were in place, baseline transmission would have been lower.

We therefore evaluate an alternative model where a change in relationship occurred after a country-specific estimated time point (*T*_*c*,*i*_):

4 $$log( {R_{t,i}} ) = \left\{ {\begin{array}{*{20}{c}} {log( {R_{0,i,1}} ) - \beta _{i,1}( {1 - m_{t,i}} )} & {t {\,}\le {\,}T_{c,i}} \\ {log( {R_{0,i,2}} ) - \beta _{i,2}( {1 - m_{t,i}} )} & {t {\,}> {\,}T_{c,i}} \end{array}} \right.$$

The framework to link *R*_*t*,*i*_ to $R_{t,i}^D$ and ultimately to the observed deaths $D_{t,i}$ remains as above. With this alternative model, we can estimate two mobility thresholds that can be interpreted as the reduction in measured mobility that would be necessary in order to achieve control pre and post- changes in the mobility-transmission relationship.

### Implementation and caveats

We estimated the joint posterior distribution of *R*_0,*i*_’s and *β*_*i*_’s using a Markov Chain Monte Carlo procedure with a Metropolis-Hasting algorithm^34^. Posterior distributions for *R*_*t*,*i*_ and $R_{t,i}^D$ can be directly obtained from the above. To ensure our parameter estimates were data-driven, we used uninformative prior distributions for *R*_0,*i*_’s (uniform in the range [0; 5]) and, *β*_*i*_’s (uniform in the range [−100; 100]).

As there are likely to be large heterogeneities in first the transmissibility between individuals and second the reporting of deaths, we assume a negative binomial likelihood by default, which allows us to estimate an over-dispersion parameter, *δ*. We used an exponential prior distribution for *δ* with a mean of 1 (equivalent to a geometric distribution). The model was also fitted using an alternative serial interval of deaths with mean 4.8 and standard deviation 2.7 days^35^. Temporal changes in epidemiological situation and in particular implementation of control strategies, can impact the serial interval by for instance shortening the ‘effective’ infectious period^36^. Assuming a time varying serial interval could affect the transmission estimates in non-trivial ways, however, in the absence of global data on serial intervals over time, an analysis which would account for time varying serial interval could not be performed.

We evaluated the correlation between estimated mobility thresholds and basic reproduction number across countries to ensure the variation in the estimated thresholds was not driven by the variation in estimated basic reproduction number (SI). Finally, as the reporting of deaths might have changed during the country-specific early phase of the epidemic, we re-estimated the mobility-transmission relationship discarding from the likelihood all days previous to the two consecutive weeks reporting each at least 10 deaths (the criteria for sustained epidemic, see SI).

For each country, the best model (with or without change in the mobility-transmission relationship) was chosen using the Deviance Information Criterion (DIC)^25^. Each mobility data-stream was used independently to estimate the parameters’ joint posterior distribution, and its fit evaluated again on the basis of its DIC.

### Evaluating model fit

We assessed whether the simple model outlined above (two or five parameters per country, *R*_0,*i*_’s and *β*_*i*_’s, and the time of the change in relationship if it occurs) captured the trends in the effective reproduction number. Independent of the mobility data, we estimated the effective reproduction number based on well-established methodology^24^ and the associated R package ‘EpiEstim’^23^. Using a Bayesian framework, the method estimates the effective reproduction number based on daily death counts:

5 $$D_{t,i}\sim P\left( {R_{t,i}^{D,EpiEstim}\mathop {\sum}\limits_{s = 0}^t {\left[ {D_{s,i}\;w_{t - s}} \right]} } \right)$$

with $R_{t,i}^{D,EpiEstim}$ the delayed effective reproduction number (i.e., reflecting transmission level at time of deaths). Weekly estimates of $R_{t,i}^{D,EpiEstim}$ were obtained assuming constant transmissibility for 7 days. The estimated $R_{t,i}^{D,EpiEstim}$’s from EpiEstim^23^ assume a Poisson distribution of reported deaths. We implemented a negative binomial model, which is equivalent to EpiEstim in the limit when there is no over-dispersion. This is critical as allowing for over-dispersion is likely to change the $R_{t,i}^{D,EpiEstim}$ estimate, especially when reported deaths are low.

For each country, we could then compare $R_{t,i}^D$ and $R_{t,i}^{D,EpiEstim}$. While $R_{t,i}^D$ relies on estimating 2 or 5 parameters (*R*_0*,i*_’s, and *β*_*i*_’s and a potential change in the relationship with mobility), $R_{t,i}^{D,EpiEstim}$ relies on estimating as many parameters as there are number of weeks in the time-series of deaths.

As well as comparing the estimated effective reproduction numbers over time, we compared the relationship between $R_{t,i}^D$ and $R_{t,i}^{D,EpiEstim}$ and mobility.

To do so, we linked death-related reproduction numbers to the earlier mobility patterns, *m*_*t,i*_, when those dying were infected. We defined an effective mobility, $m_{i,t}^{eff}$, at time t that characterises the mobility at the time of infection of those who died at time *t*:

6 $$R_{t,i}^D = R_{0,i}\;e^{ - \beta _i\left( {1 - m_{i,t}^{eff}} \right)}$$

Thus,

7 $$R_{t,i}^D = \mathop {\sum}\limits_{s = 0}^t {R_{s,i}\;h_{t - s}} = \mathop {\sum}\limits_{s = 0}^t {R_{0,i}\;e^{ - \beta _i\;\left( {1 - m_{i,s}} \right)}} \;h\left( {t - s} \right)$$

(where *h(t − s)* is the infection-to-death interval distribution). Therefore, the effective mobility is:

8 $$m_{i,t}^{eff} = 1 + \frac{1}{{\beta _i}}\; log \left( {\mathop {\sum}\limits_{s = 0}^t {e^{ - \beta _i\;\left( {1 - m_{i,s}} \right)}} \;h\left( {t - s} \right)} \right)$$

We can now plot $R_{t,i}^D$ and $R_{t,i}^{D,EpiEstim}$ against the effective mobility at the time of infection.

Interestingly, estimating the effective mobility experienced by those dying on day *t* relies on assumptions about the functional relationship between mobility and *R*_*t*_. Intuitively, assuming that the effective mobility is equal to the past mobility weighted by the infection-to-death interval is equivalent to assuming a linear relationship between mobility and the reproduction number. Assuming $R_{t,i} = R_{0,i} - \beta _i\;( {1 - m_{t,i}} )$, then, following the same logic as above, we have: $m_{i,t}^{eff} = 1 - ( {\mathop {\sum}\nolimits_{s = 0}^t {( {1 - m_{i,s}} )h( {t - s} )} } )$.

Once the relationship between mobility and *R*_*t*,*i*_ is characterised, we can evaluate the posterior distribution for *R*_*t*,*i*_ for any mobility including when *R*_*t*,*i*_ = 1.

### Data ethics

All data used consist of nationally aggregated and anonymized records already available publicly. Terms of use for mobility data has been granted to use such data in the context of the COVID-19 pandemic (https://www.google.com/covid19/mobility, https://covid19.apple.com/mobility). No ethical approval was required for the work presented.

### Reporting summary

Further information on research design is available in the Nature Research Reporting Summary linked to this article.

## Supplementary information

Reporting Summary

Supplementary Information

## Data Availability

The datasets on deaths from WHO and ECDC dashboard consists of aggregated COVID-19-confirmed case and death counts publicly released (https://covid19.who.int, https://opendata.ecdc.europa.eu/covid19). For the mobility data, Apple’s and Google’s mobility data consists of aggregated, anonymized sets of data from users who have chosen to turn on the location history setting.

## Code availability

All analyses were performed using the statistical software R (version 3.5.1) and all the code used is available on GitHub (https://github.com/pnouvellet/Mobility.2.0).
