# Supplementary material for: Reduction in mobility and COVID-19 transmission
Source: Nat Commun. 2021 Feb 17;12:1090. doi: 10.1038/s41467-021-21358-2 (PMC7889876; doi:10.1038/s41467-021-21358-2)
Supplement: Supplementary file 2 — Supplementary Information [file 41467_2021_21358_MOESM2_ESM.pdf]

Supplementary Information for

Reduction in mobility and COVID-19 transmission

Pierre Nouvellet*[1,2]; Sangeeta Bhatia[1]; Anne Cori[1]; Kylie E C Ainslie[1]; Marc Baguelin[1]; Samir Bhatt[1]; Adhiratha Boonyasiri[1]; Nicholas F Brazeau[1]; Lorenzo Cattarino[1]; Laura V Cooper[1]; Helen Coupland[1]; Zulma M Cucunuba[1]; Gina Cuomo-Dannenburg[1]; Amy Dighe[1]; Bimandra A Djaafara[1]; Ilaria Dorigatti[1]; Oliver D Eales[1]; Sabine L van Elsland[1]; Fabricia F Nascimento[1]; Richard G FitzJohn[1]; Katy A M Gaythorpe[1]; Lily Geidelberg[1]; William D Green[1]; Arran Hamlet[1]; Katharina Hauck[1]; Wes Hinsley[1]; Natsuko Imai[1]; Benjamin Jeffrey[1]; Edward Knock[1]; Daniel J Laydon[1]; John A Lees[1]; Tara Mangal[1]; Thomas A Mellan[1]; Gemma Nedjati-Gilani[1]; Kris V Parag[1]; Margarita Pons-Salort[1]; Manon Ragonnet-Cronin[1]; Steven Riley[1]; H Juliette T Unwin[1]; Robert Verity[1]; Michaela A C Vollmer[1]; Erik Volz[1]; Patrick G T Walker[1]; Caroline E Walters, Ph.D.[1]; Haowei Wang[1]; Oliver J Watson[1]; Charles Whittaker[1]; Lilith K Whittles[1]; Xiaoyue Xi[1]; Neil M. Ferguson[1]; Christl A. Donnelly[1,3].

Affiliations:

1. MRC Centre for Global Infectious Disease Analysis, J-IDEA; Department of Infectious Disease Epidemiology, Imperial College London

2. School of Life Sciences, University of Sussex,

3. Department of Statistics, University of Oxford

Corresponding author: Pierre Nouvellet, pierre.nouvellet@sussex.ac.uk ; Christl A Donnelly, christl.donnelly@stats.ox.ac.uk and c.donnelly@imperial.ac.uk

Contents

Supplementary note 1: Summary of epidemiological and mobility data per country 3

Correlation in mobility estimates 17

Supplementary note 2: Mobility thresholds by countries 18

Supplementary note 3: Improved fit using individual data stream in selected countries....45

Supplementary note 4: Mobility thresholds by countries 48

Supplementary note 5: Correlation between R_0 and the mobility threshold....52

Supplementary note 6: Sensitivity of estimated parameters to early epidemic dynamics....53

Supplementary note 7: Sensitivity of estimated mobility thresholds to the serial interval distribution 54

Supplementary note 8: Modelling over-dispersion 58

Supplementary note 9: Linking current transmission to transmission measured at deaths....61

Supplementary note 10: Additional information for the inference 63

Supplementary note 1: Summary of epidemiological and mobility data per country

Overall, mobility saw a sharp decline followed by a gradual recovery, with large variability across countries.

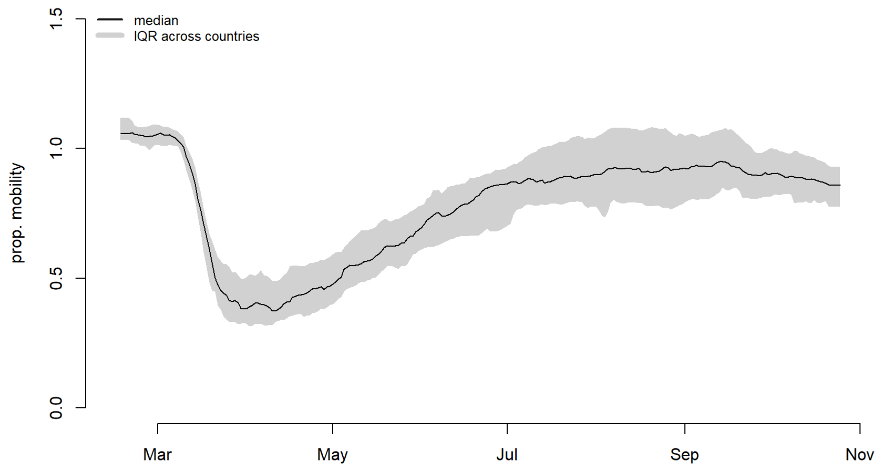

Supplementary Figure 1: Temporal variations in Apple-Google mobility trends, median and interquartile range (IQR) across the 52 countries included in the analysis.

The plots below summarise the incidence of deaths, and mobility data available in each country.

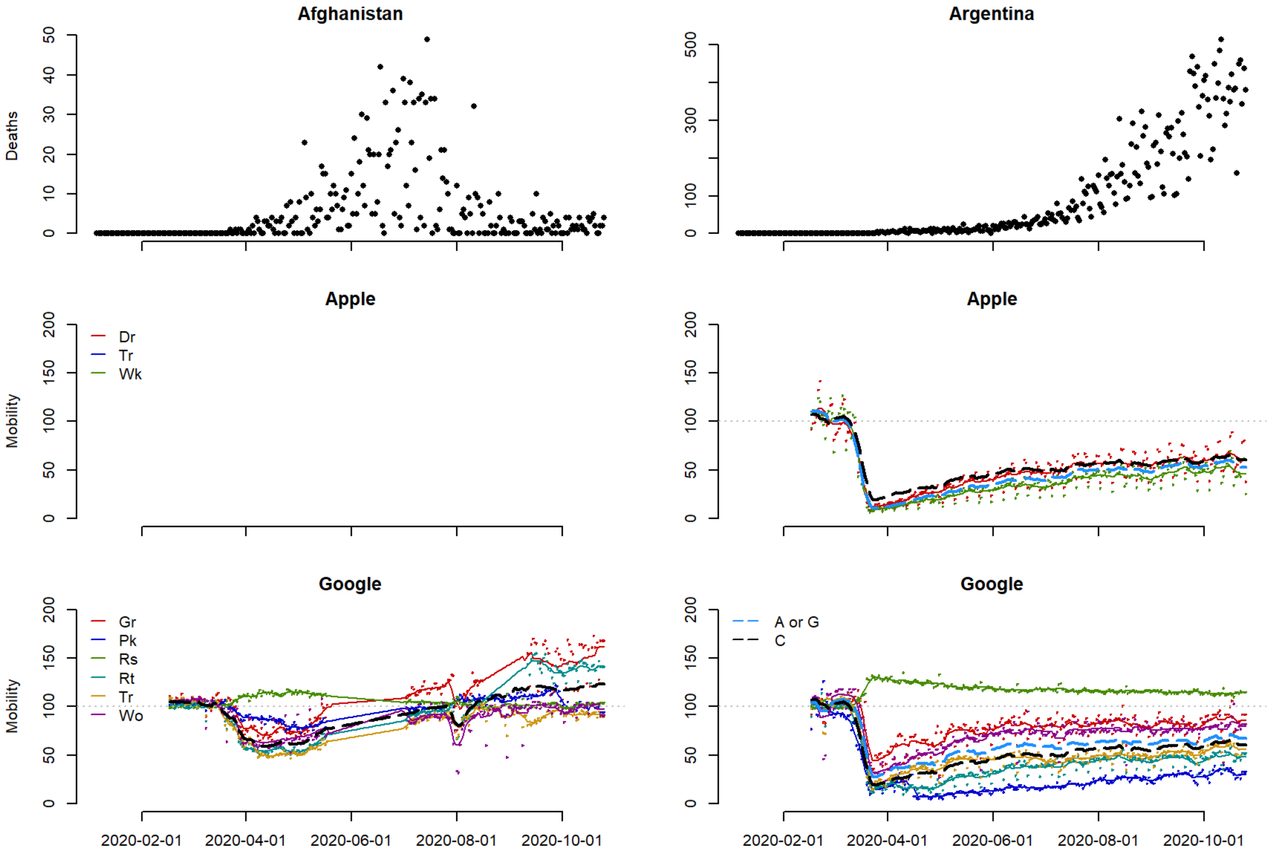

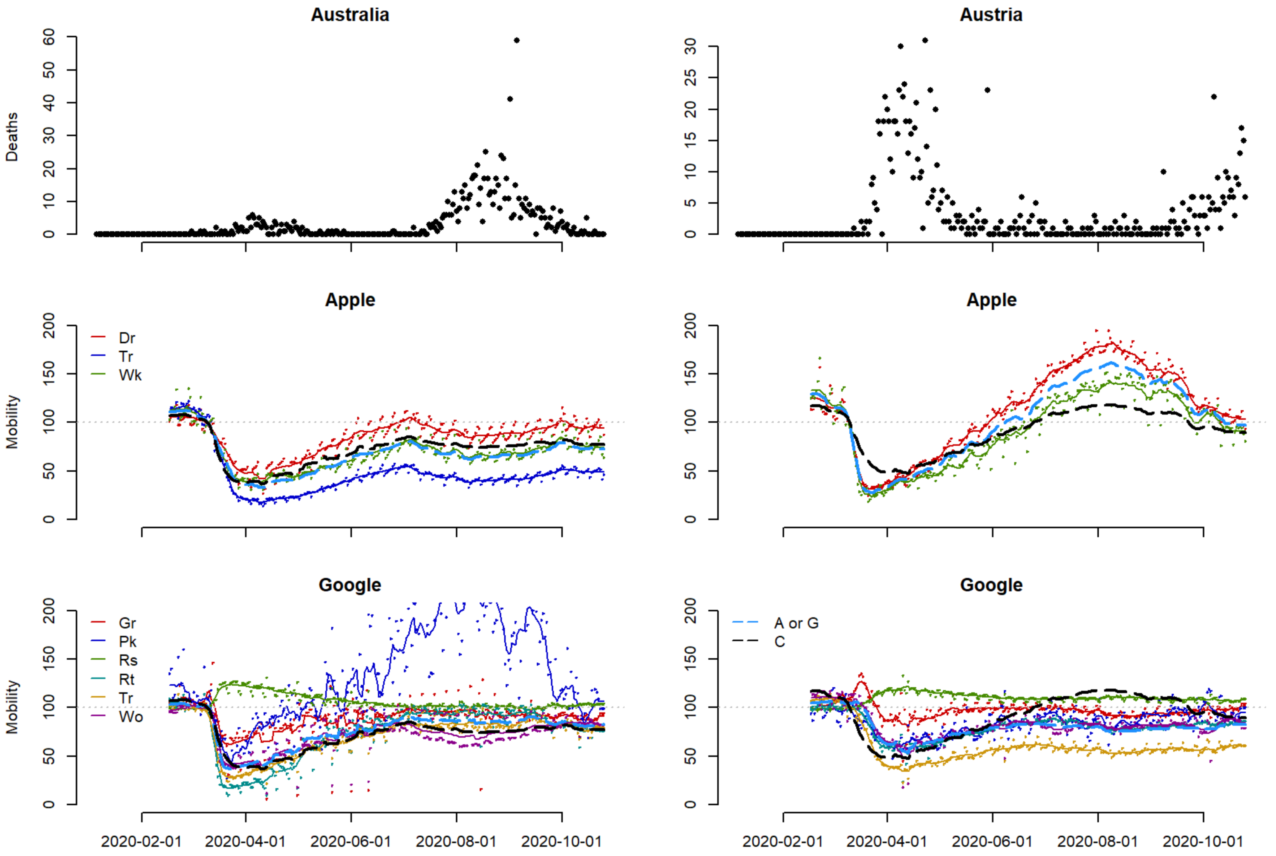
Australia
Austria
Deaths
Apple
Mobility
Dr
Tr
Wk
Google
Gr
Pk
Rs
Rt
Tr
Wo
A or G
C
2020-02-01
2020-04-01
2020-06-01
2020-08-01
2020-10-01

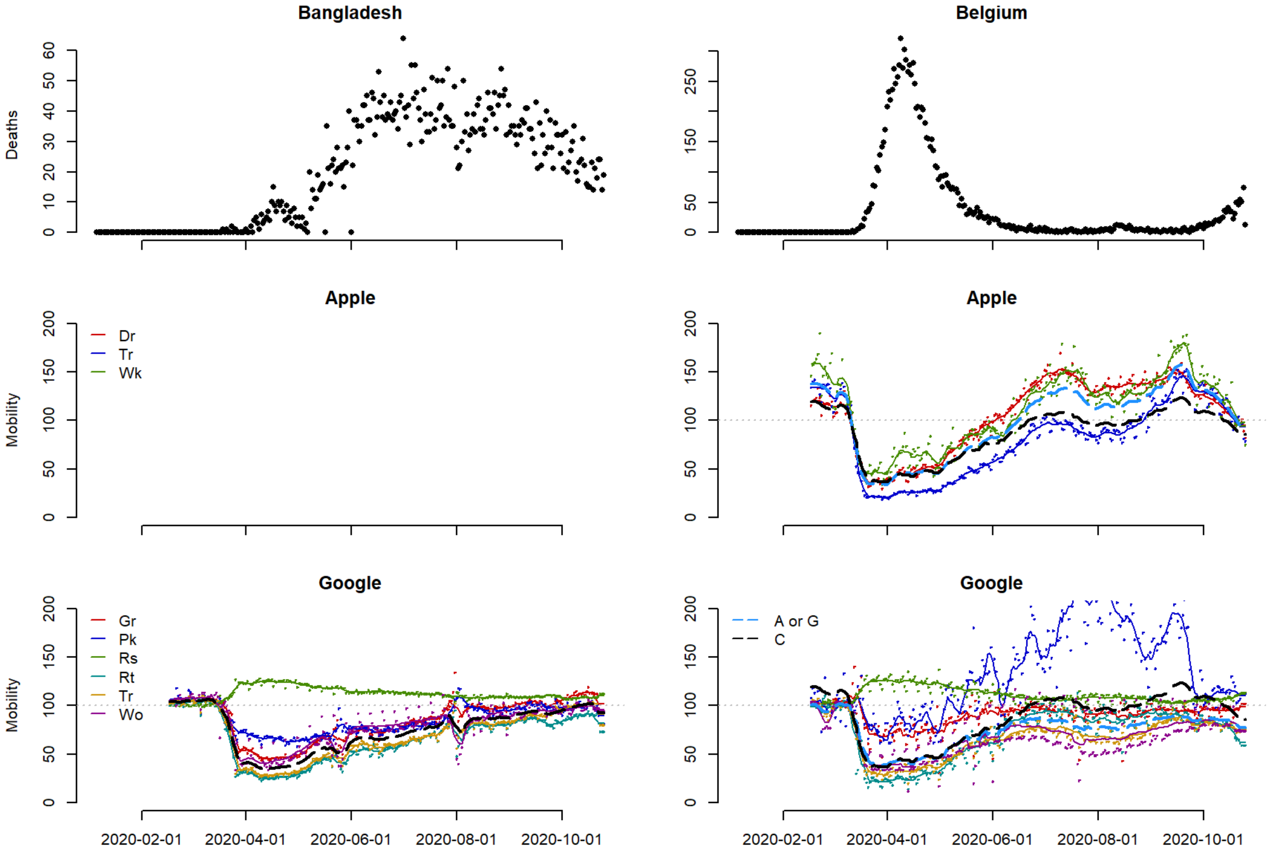
Bangladesh
Belgium
Deaths
Apple
Mobility
Dr
Tr
Wk
Google
Gr
Pk
Rs
Rt
Tr
Wo
A or G
C
2020-02-01
2020-04-01
2020-06-01
2020-08-01
2020-10-01

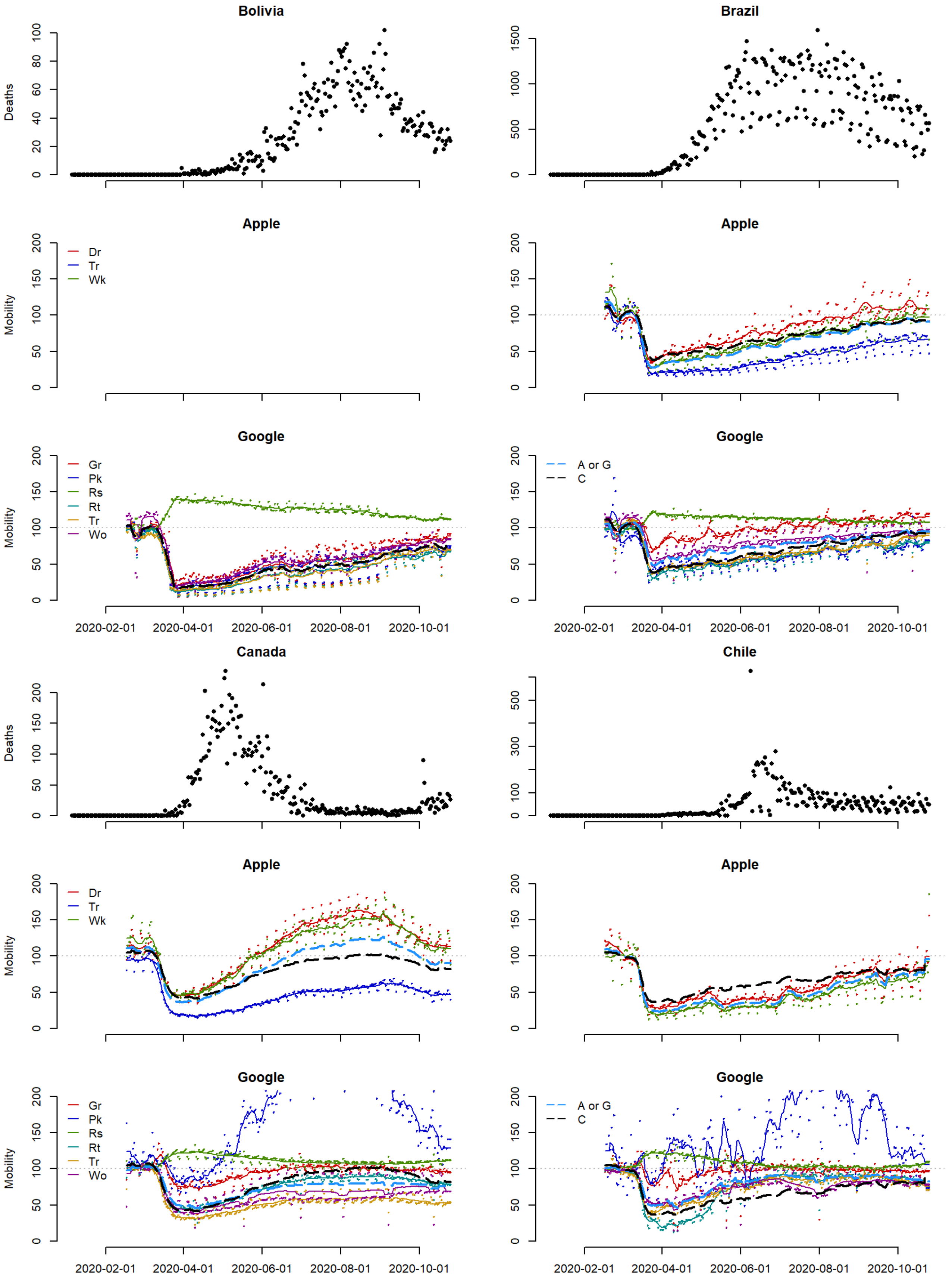
Bolivia
Brazil
Canada
Chile
Apple
Google
Deaths
Mobility
Dr
Tr
Wk
Gr
Pk
Rs
Rt
Tr
Wo
A or G
C
2020-02-01
2020-04-01
2020-06-01
2020-08-01
2020-10-01

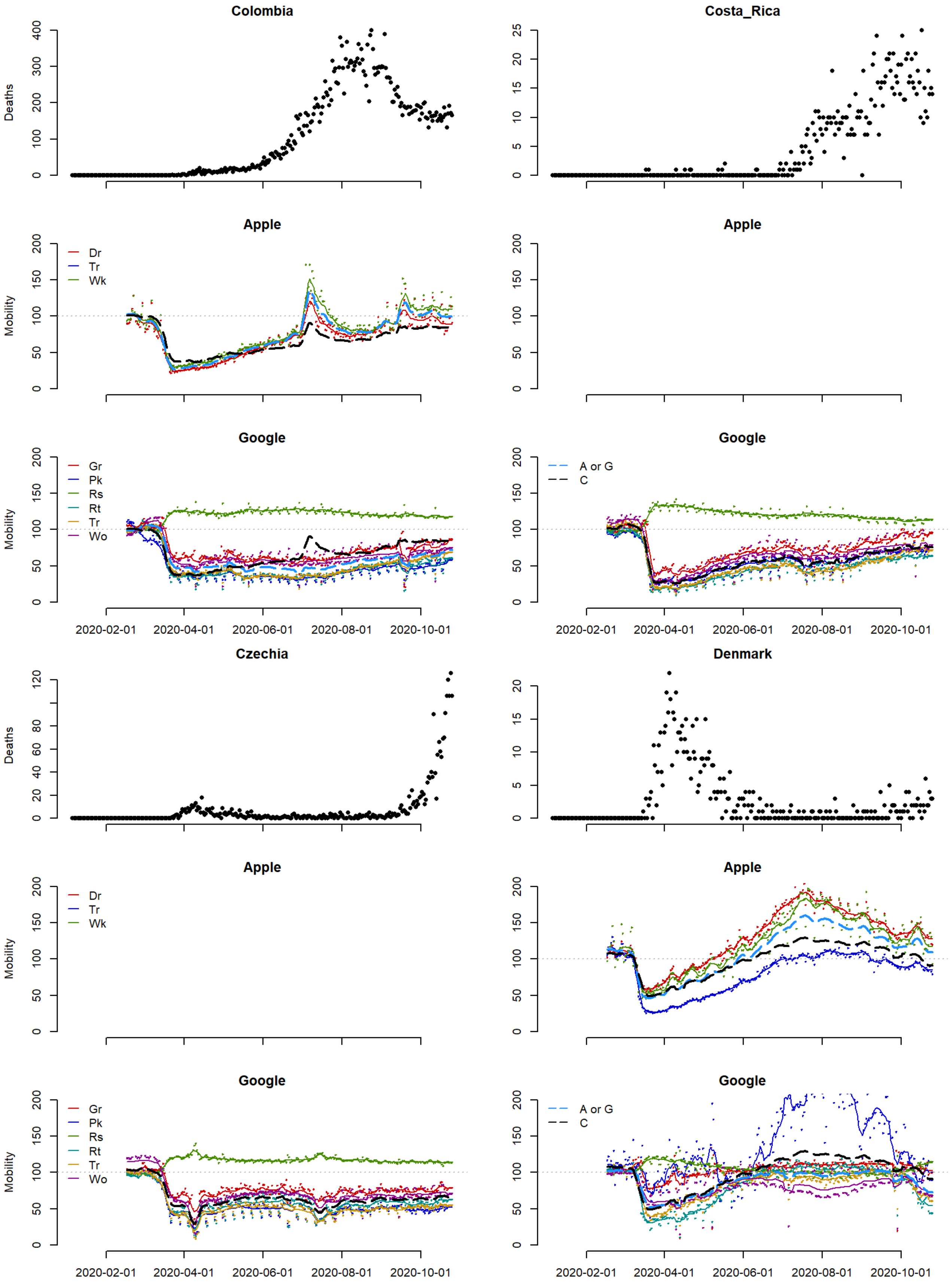
Colombia
Costa_Rica
Deaths
Apple
Dr
Tr
Wk
Mobility
Google
Gr
Pk
Rs
Rt
Tr
Wo
A or G
C
2020-02-01
2020-04-01
2020-06-01
2020-08-01
2020-10-01
Czechia
Denmark

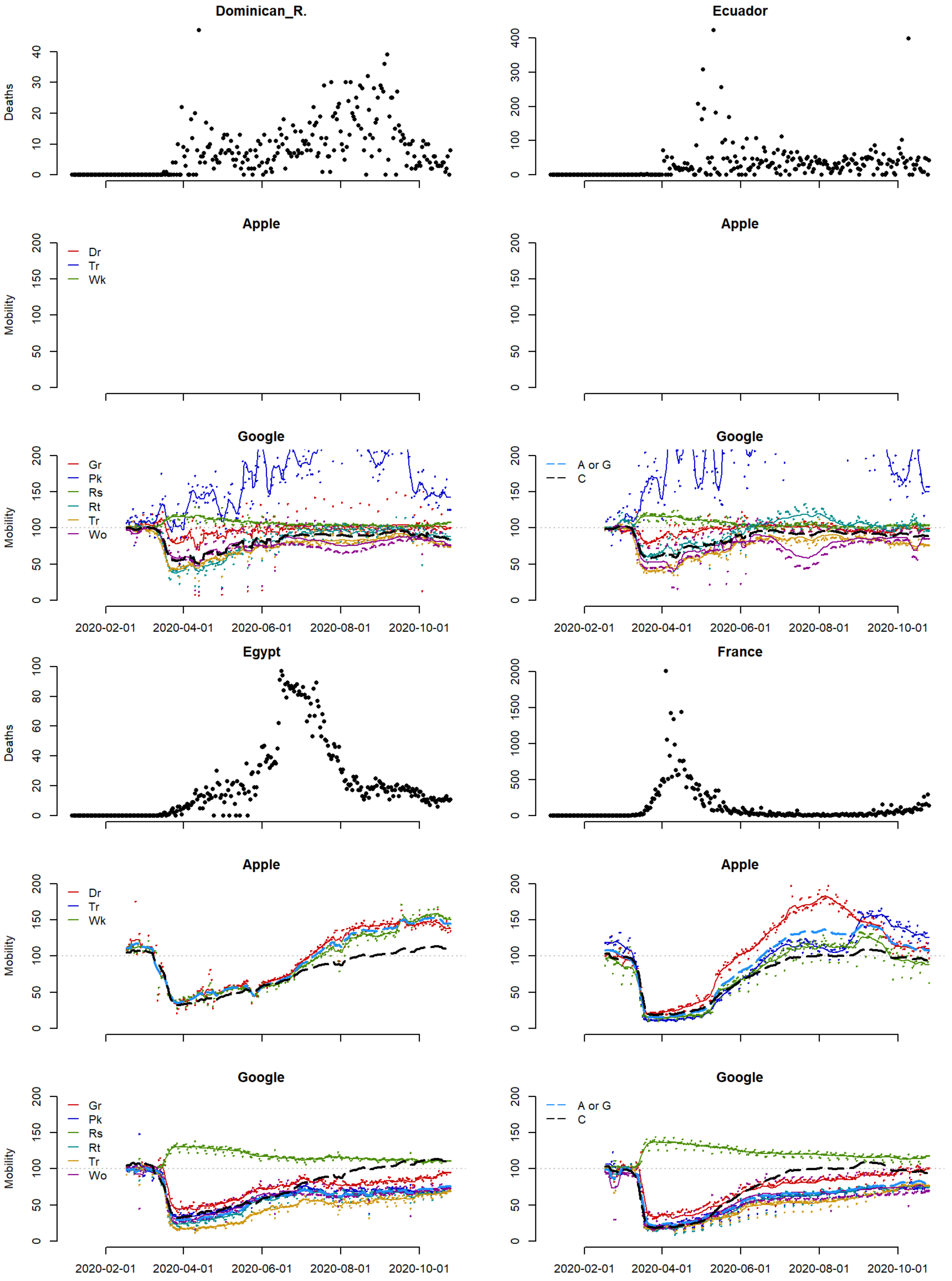
Dominican_R.
Ecuador
Deaths
Apple
Mobility
Dr
Tr
Wk
Google
Gr
Pk
Rs
Rt
Tr
Wo
A or G
C
2020-02-01
2020-04-01
2020-06-01
2020-08-01
2020-10-01
Egypt
France

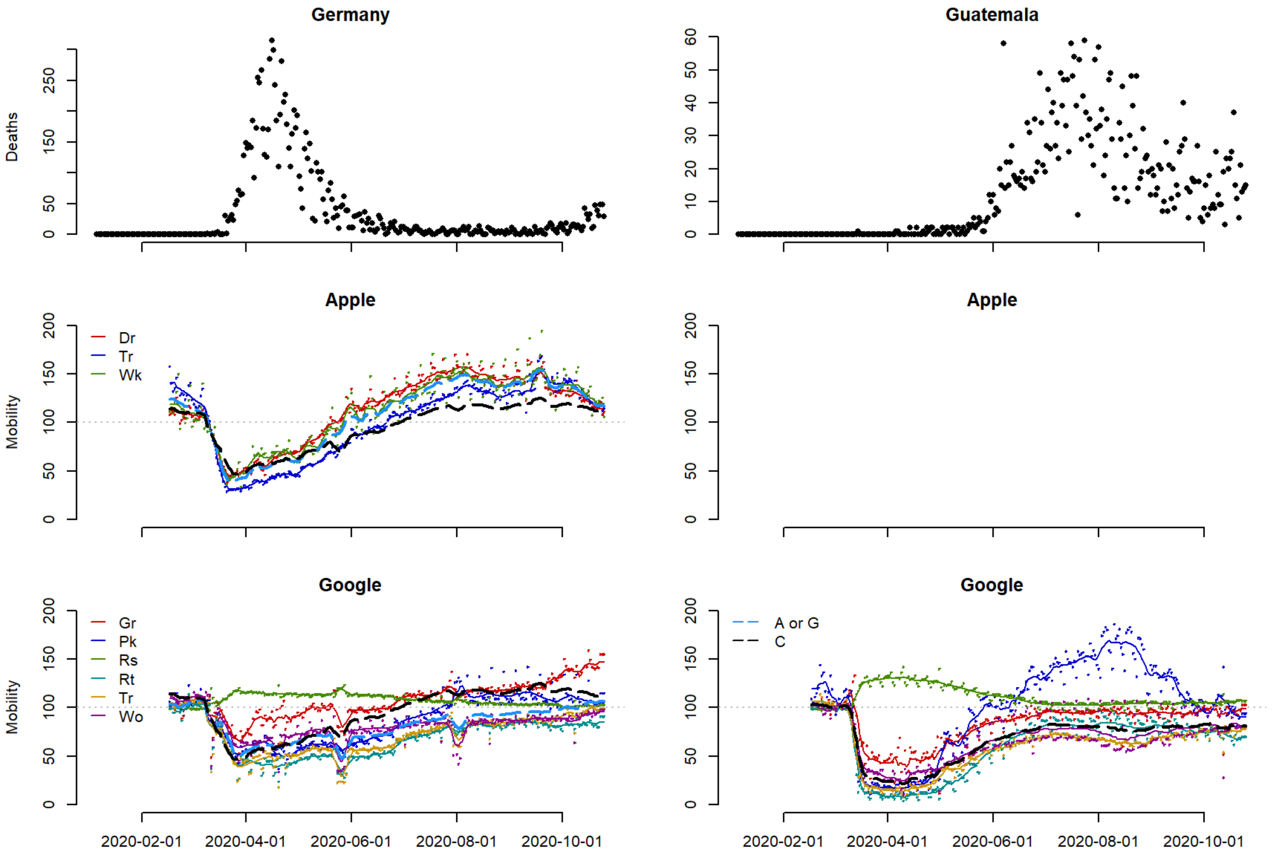
Germany
Guatemala
Deaths
Apple
Google
Mobility
Dr
Tr
Wk
Gr
Pk
Rs
Rt
Tr
Wo
A or G
C
2020-02-01
2020-04-01
2020-06-01
2020-08-01
2020-10-01

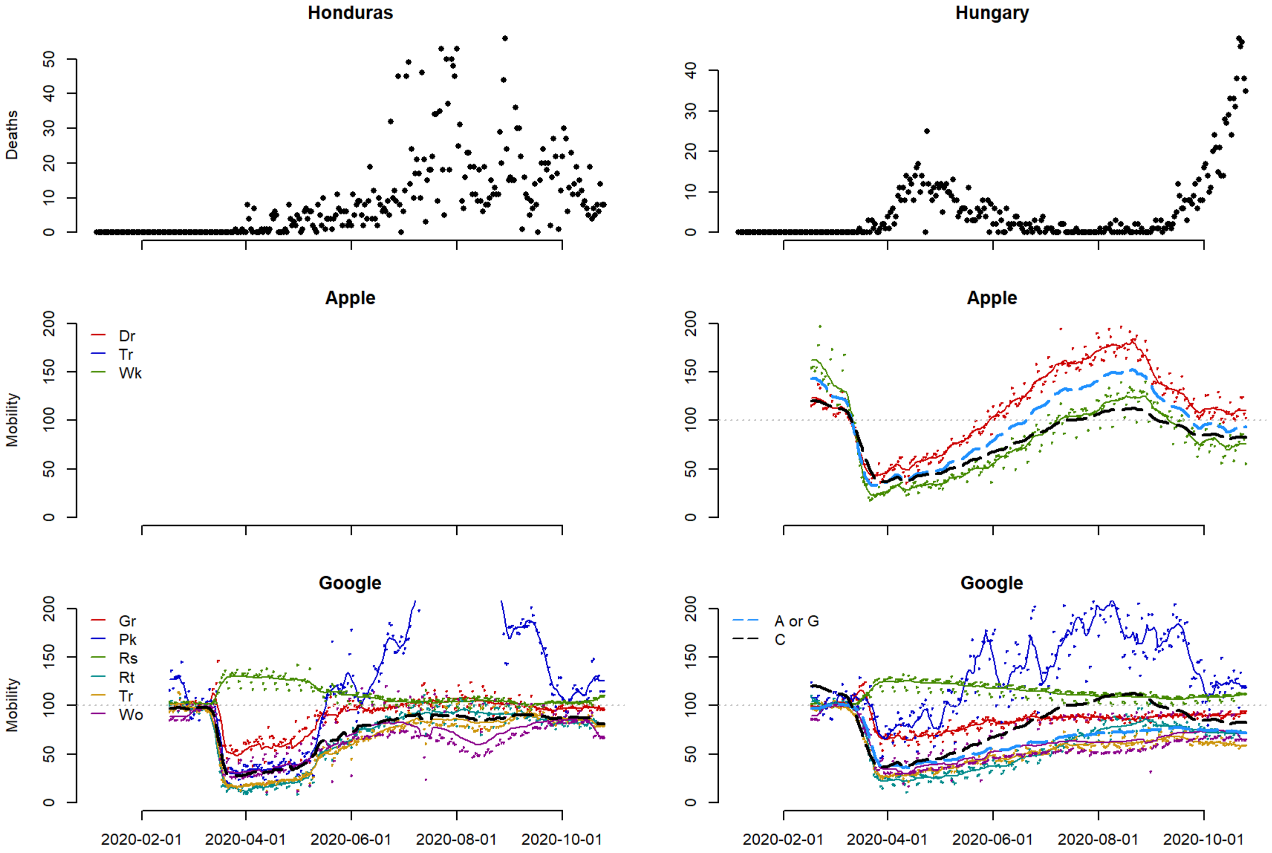
Honduras
Hungary
Deaths
Apple
Google
Mobility
Dr
Tr
Wk
Gr
Pk
Rs
Rt
Tr
Wo
A or G
C
2020-02-01
2020-04-01
2020-06-01
2020-08-01
2020-10-01

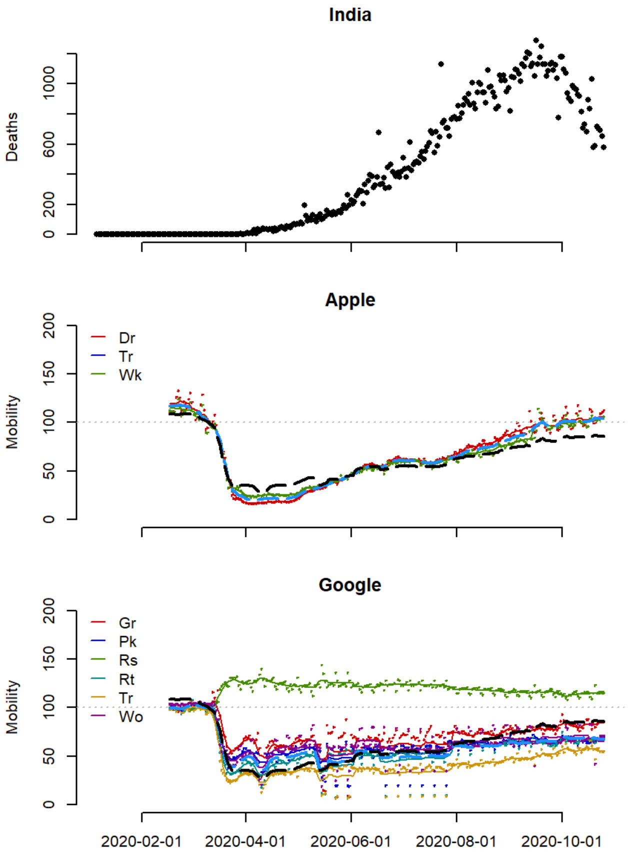
India
Deaths
0
200
600
1000
Apple
Mobility
0
50
100
150
200
Dr
Tr
Wk
Google
Gr
Pk
Rs
Rt
Tr
Wo
2020-02-01
2020-04-01
2020-06-01
2020-08-01
2020-10-01

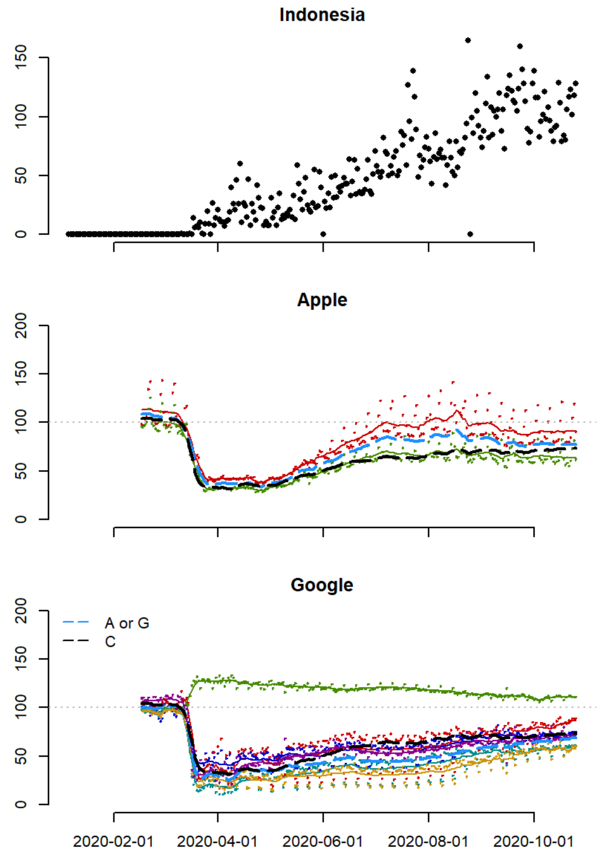
Indonesia
0
50
100
150
Apple
0
50
100
150
200
Google
A or G
C
2020-02-01
2020-04-01
2020-06-01
2020-08-01
2020-10-01

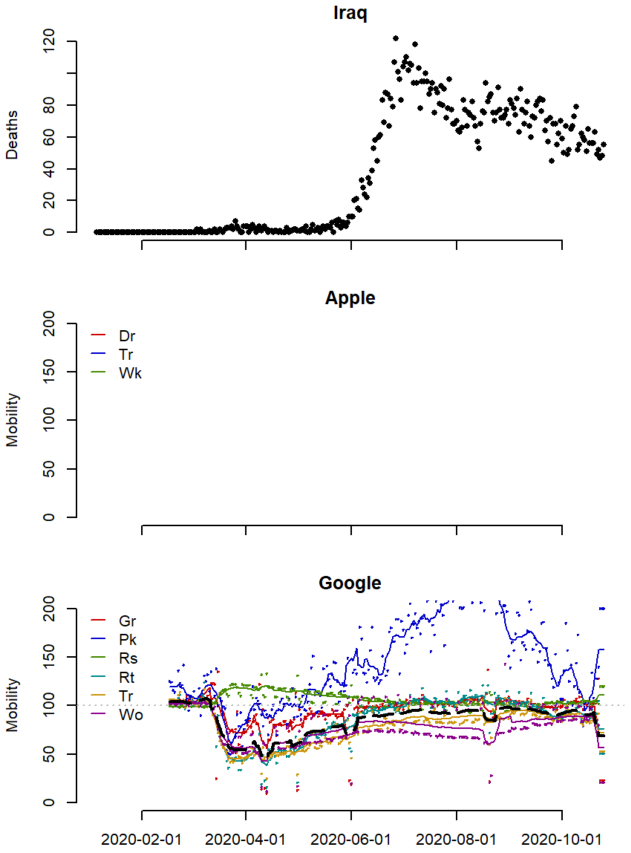
Iraq
Deaths
0
20
40
60
80
120
Apple
Mobility
0
50
100
150
200
Dr
Tr
Wk
Google
Gr
Pk
Rs
Rt
Tr
Wo
2020-02-01
2020-04-01
2020-06-01
2020-08-01
2020-10-01

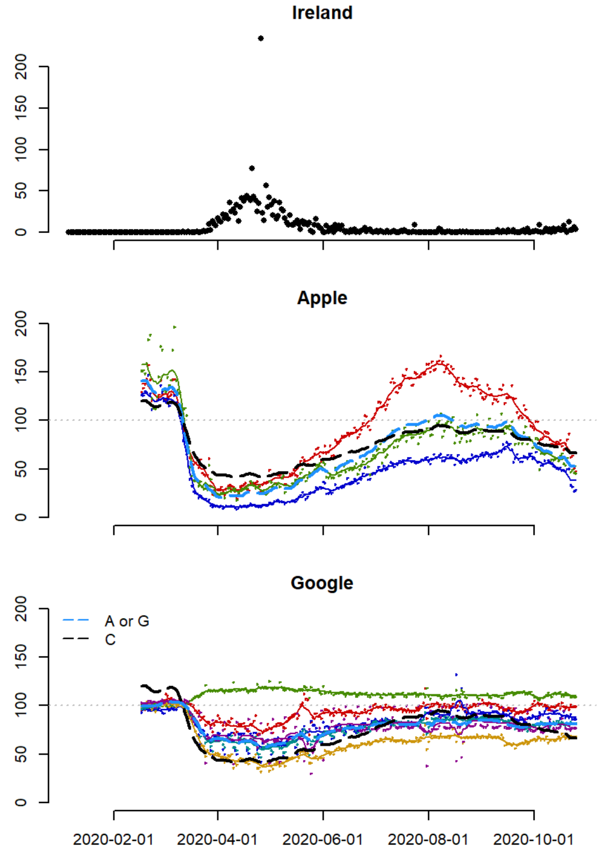
Ireland
0
50
100
150
200
Apple
Google
A or G
C
2020-02-01
2020-04-01
2020-06-01
2020-08-01
2020-10-01

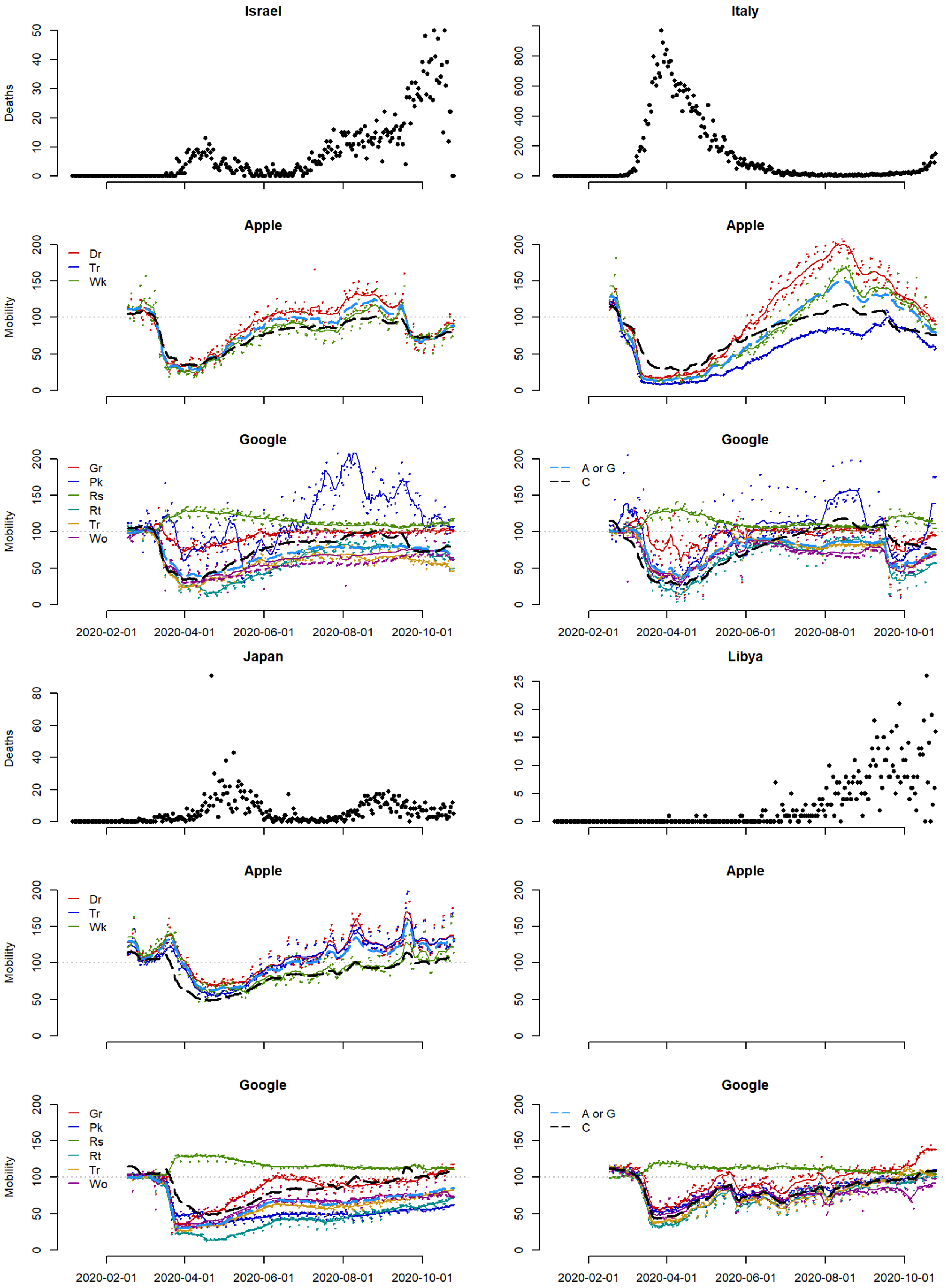
Israel
Italy
Japan
Libya
Apple
Google
Deaths
Mobility
Dr
Tr
Wk
Gr
Pk
Rs
Rt
Wo
A or G
C
2020-02-01
2020-04-01
2020-06-01
2020-08-01
2020-10-01

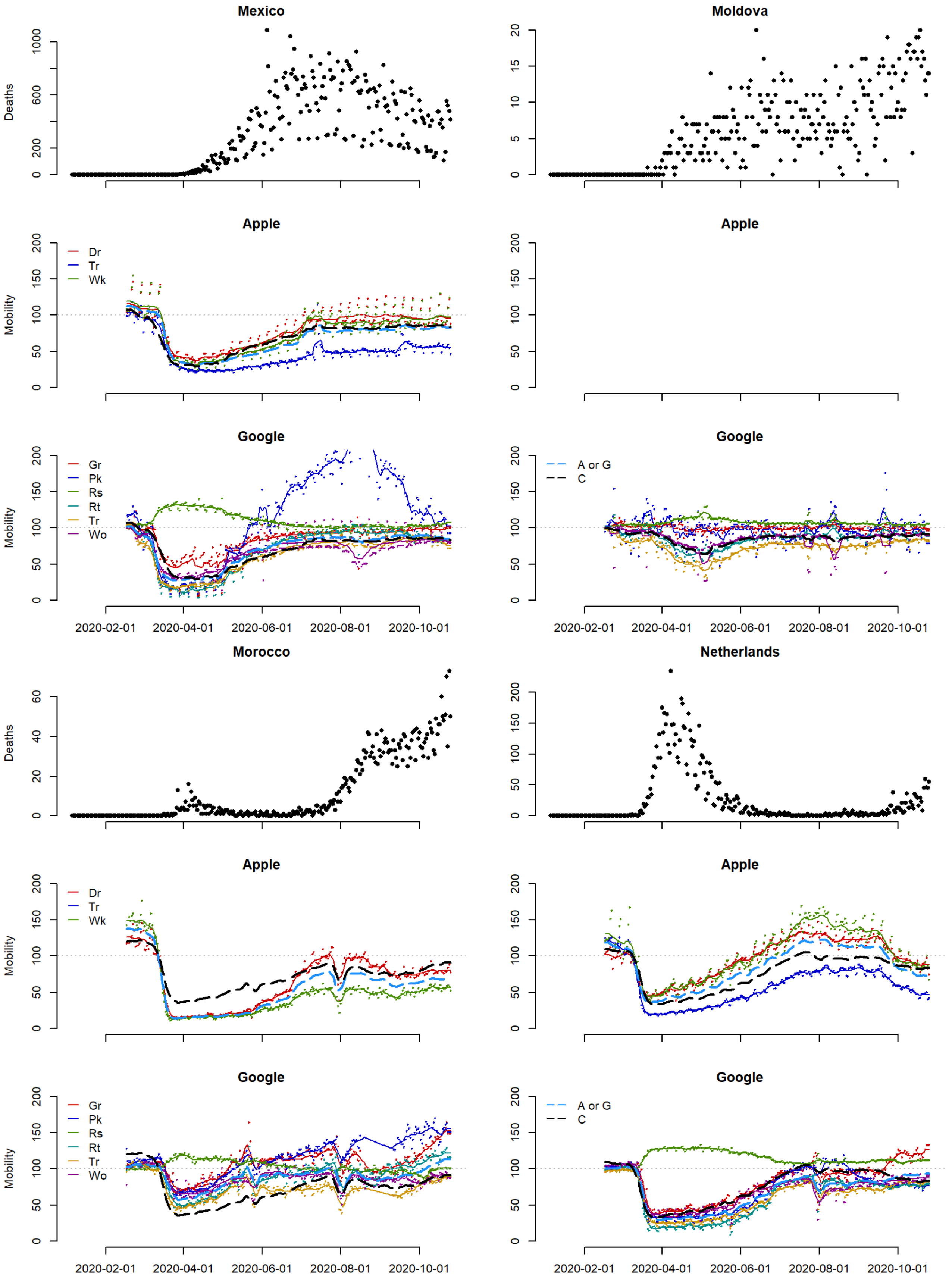
Mexico
Moldova
Deaths
Apple
Mobility
Dr
Tr
Wk
Google
Gr
Pk
Rs
Rt
Tr
Wo
A or G
C
2020-02-01
2020-04-01
2020-06-01
2020-08-01
2020-10-01
Morocco
Netherlands

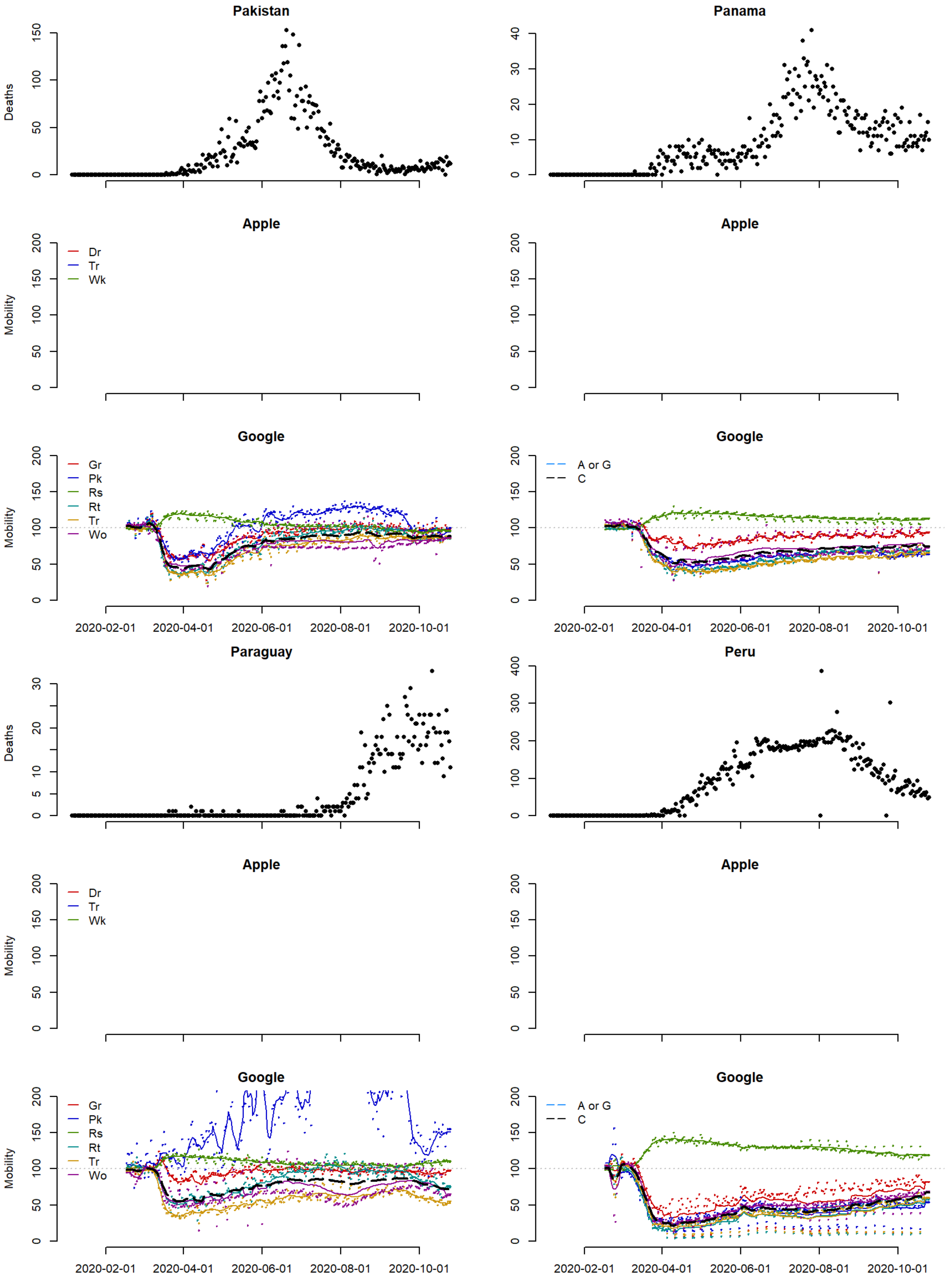
Pakistan
Panama
Deaths
Apple
Mobility
Dr
Tr
Wk
Google
Gr
Pk
Rs
Rt
Tr
Wo
A or G
C
2020-02-01
2020-04-01
2020-06-01
2020-08-01
2020-10-01
Paraguay
Peru

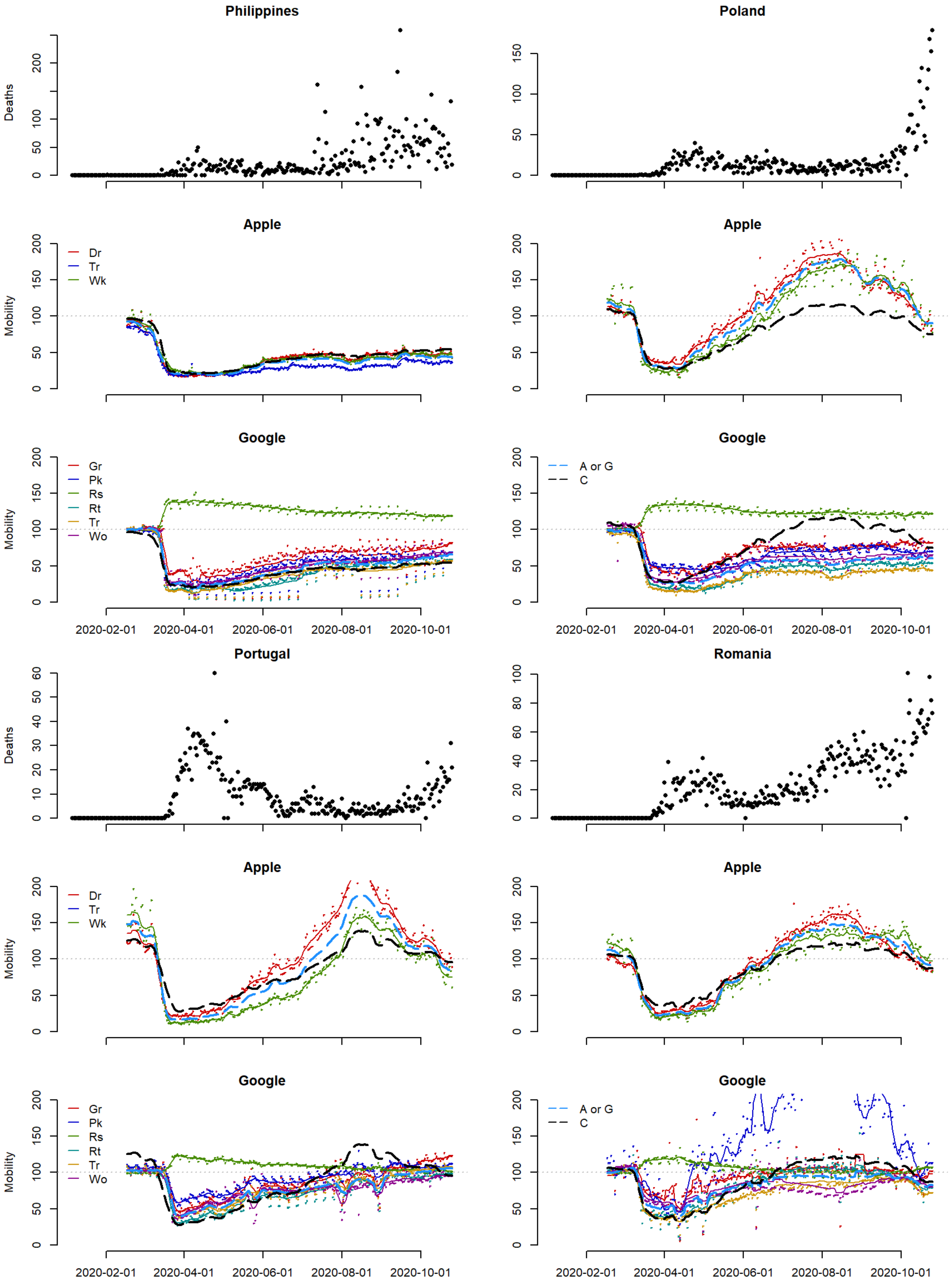
Philippines
Poland
Deaths
Apple
Mobility
Dr
Tr
Wk
Google
Gr
Pk
Rs
Rt
Tr
Wo
A or G
C
2020-02-01
2020-04-01
2020-06-01
2020-08-01
2020-10-01
Portugal
Romania

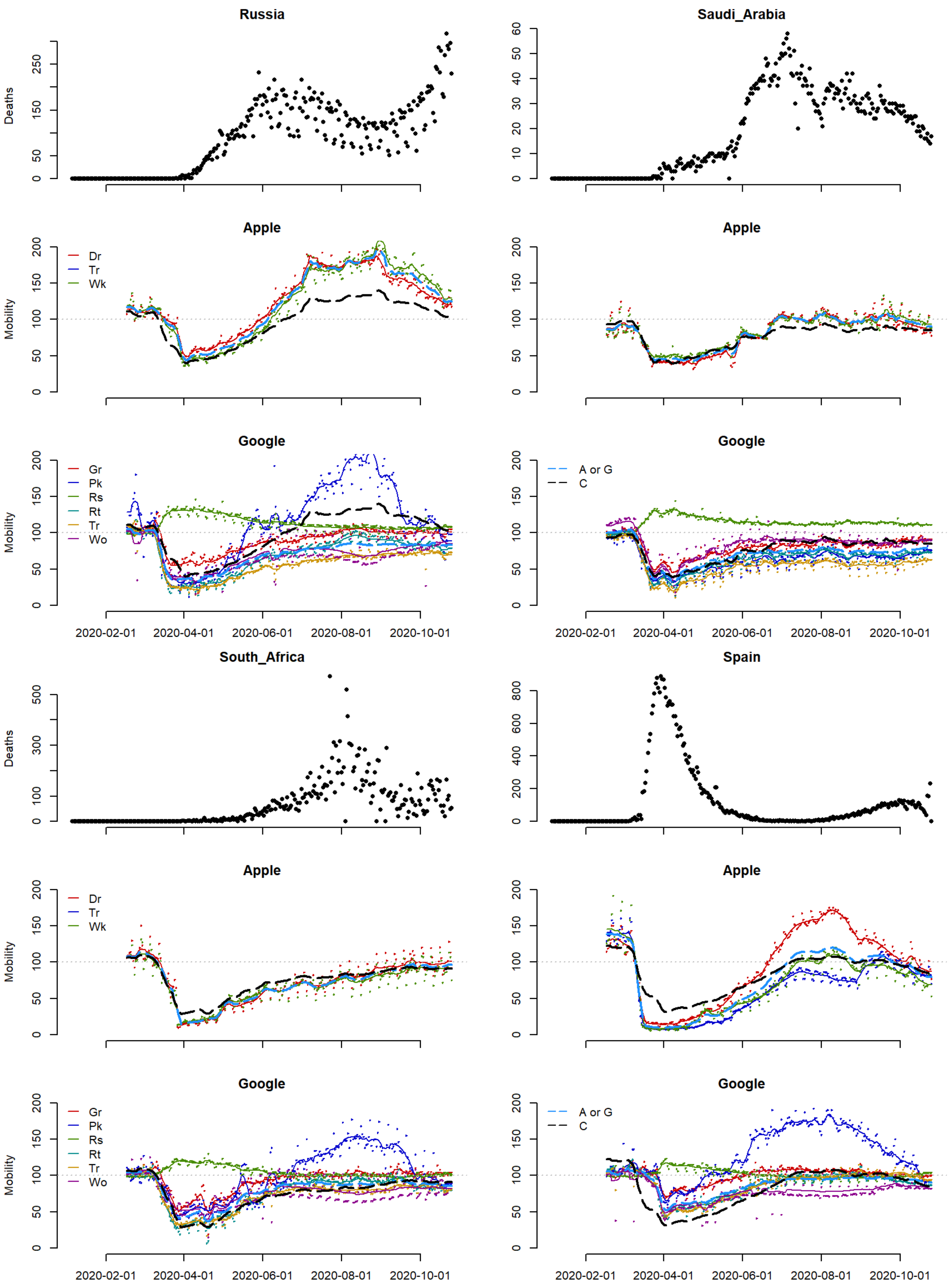
Russia
Saudi_Arabia
South_Africa
Spain
Apple
Google
Deaths
Mobility
Dr
Tr
Wk
Gr
Pk
Rs
Rt
Wo
A or G
C
2020-02-01
2020-04-01
2020-06-01
2020-08-01
2020-10-01

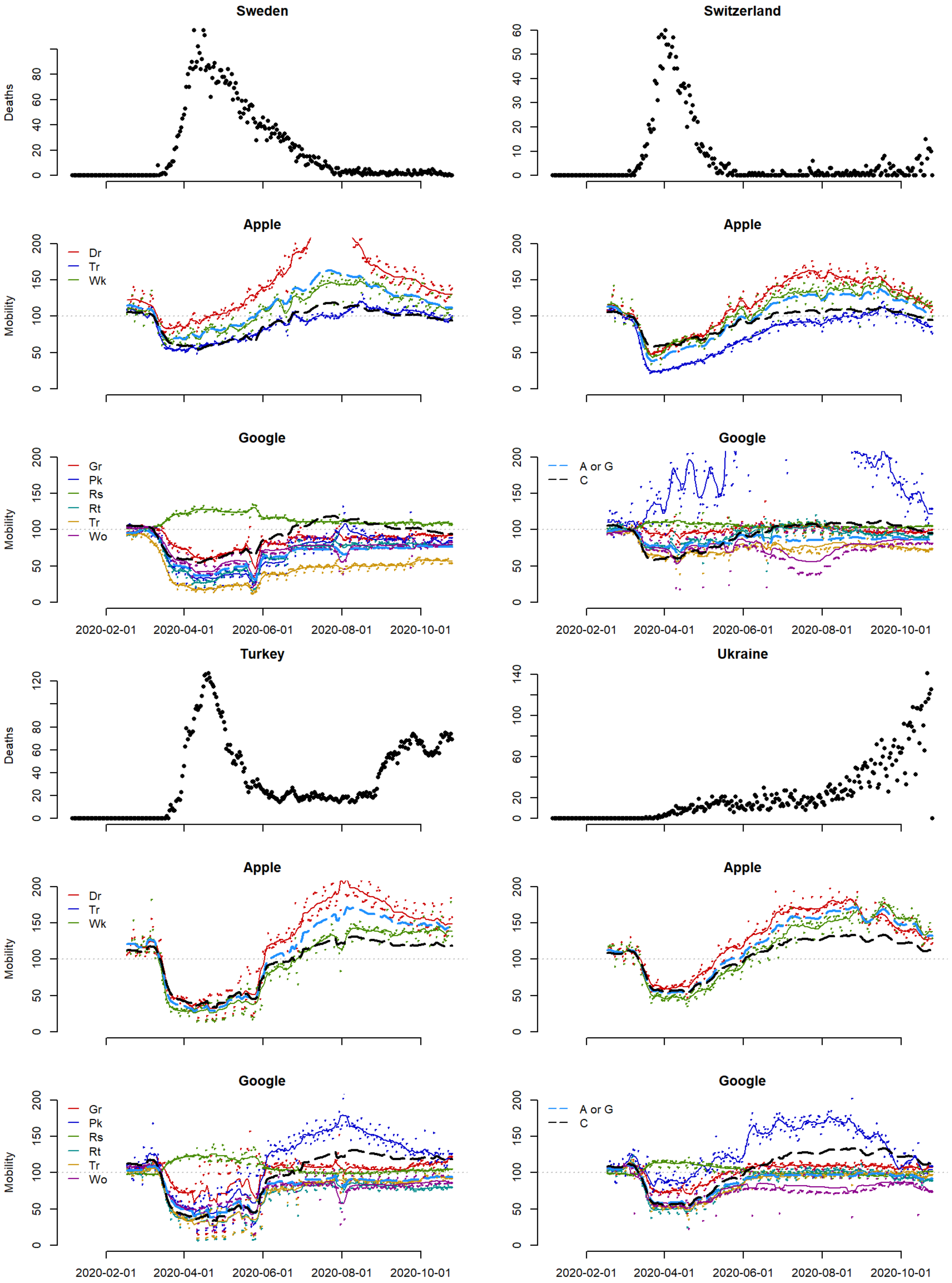
Sweden
Switzerland
Deaths
Apple
Google
Mobility
Dr
Tr
Wk
Gr
Pk
Rs
Rt
Tr
Wo
A or G
C
2020-02-01
2020-04-01
2020-06-01
2020-08-01
2020-10-01
Turkey
Ukraine

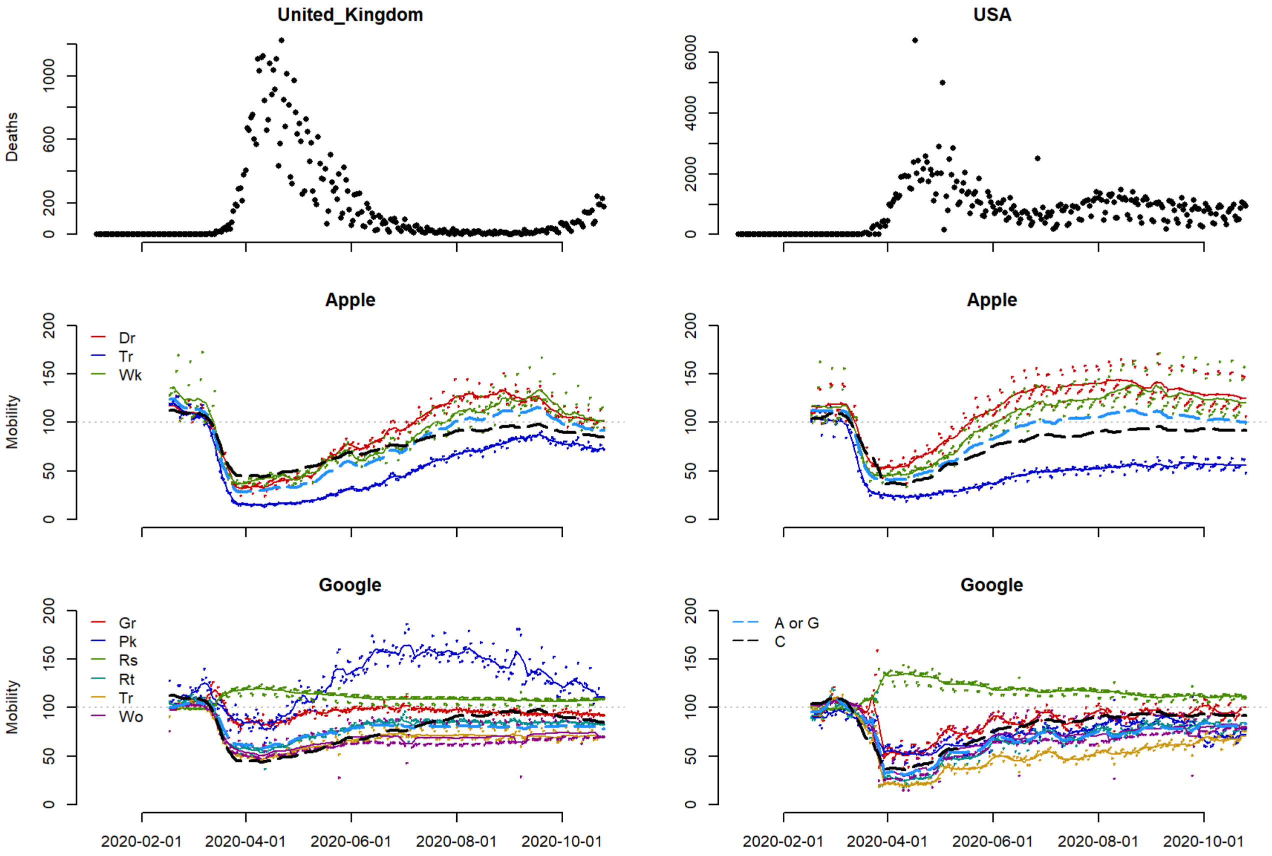

Supplementary Figure 2: Incidence of deaths and mobility data available in each country. Each stream of mobility raw data is represented with dots and with colored lines represent our smoothed mobility measure. Dashed light blue lines represent either Apple or Google (A or G) mobility measures combine all/some of the respective data streams, while the black dashed lines represent the combined (C) Apple-Google mobility estimates.

Correlation in mobility estimates

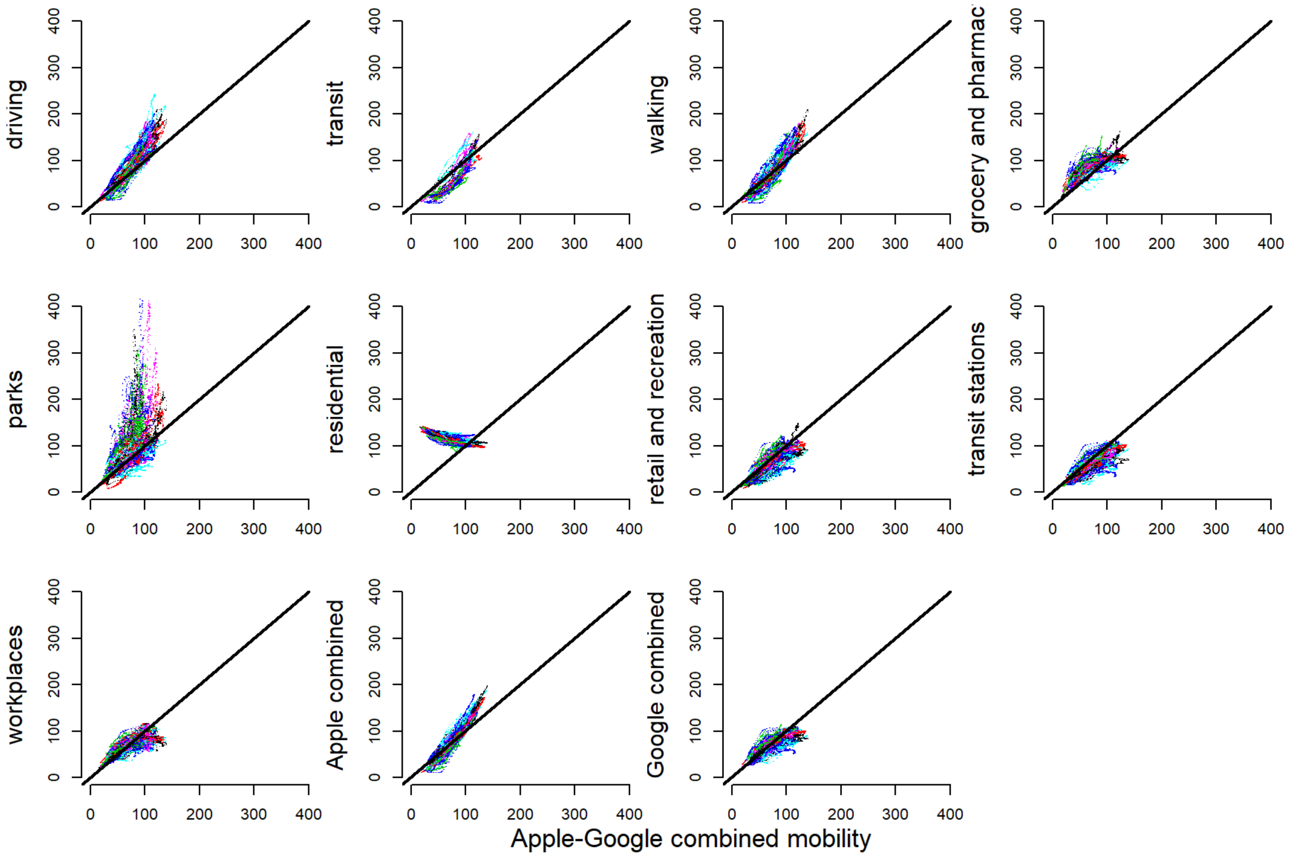

Supplementary Figure 3: Comparison of each mobility data stream against the Apple-Google data stream. The Google stream reflecting 'park' and 'residential' mobility show some clear discrepancy compared to other streams. This is likely reflecting the allowance for essential movement linked to subsistence and exercise.

Supplementary note 2: Mobility thresholds by countries

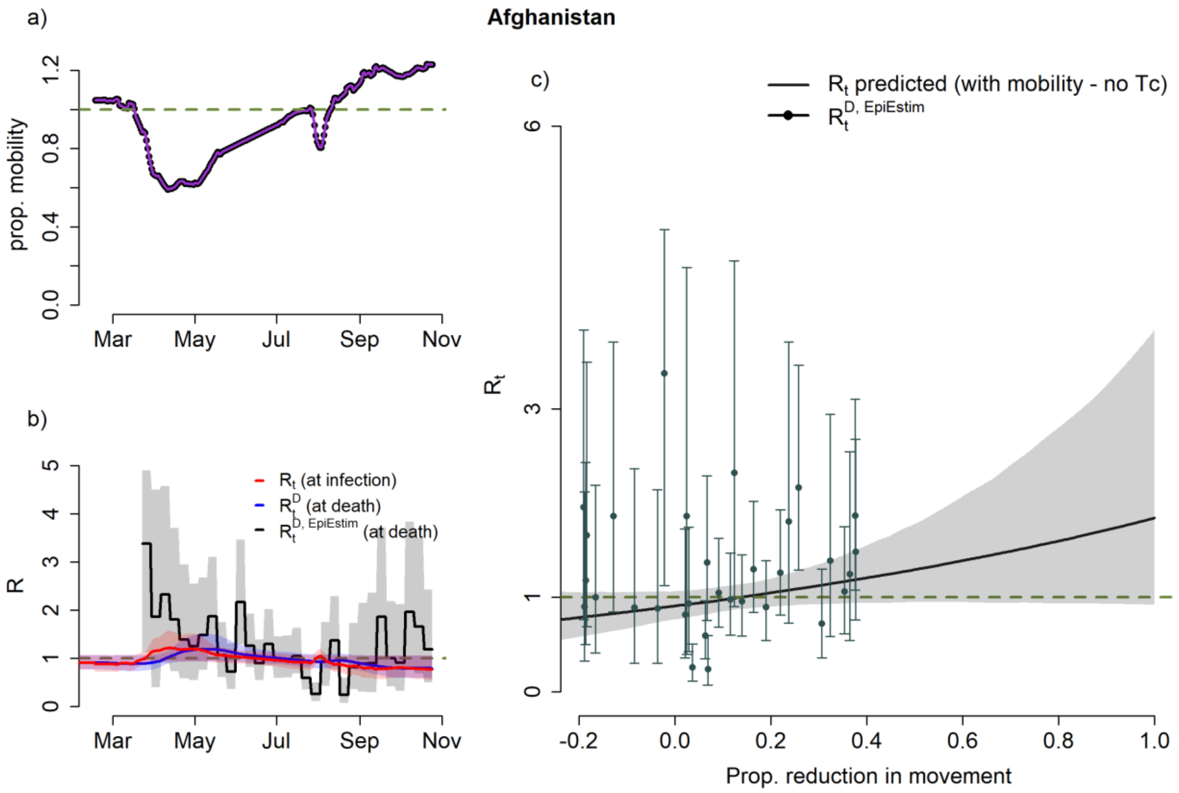

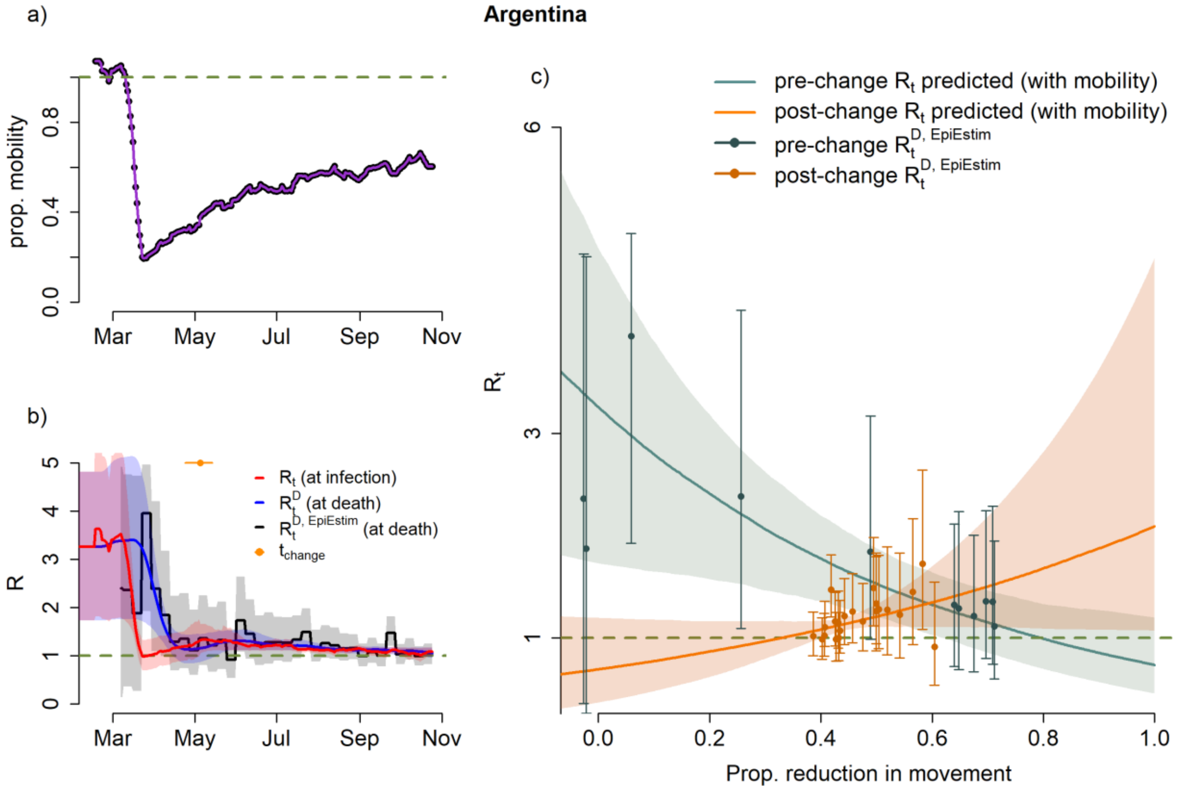

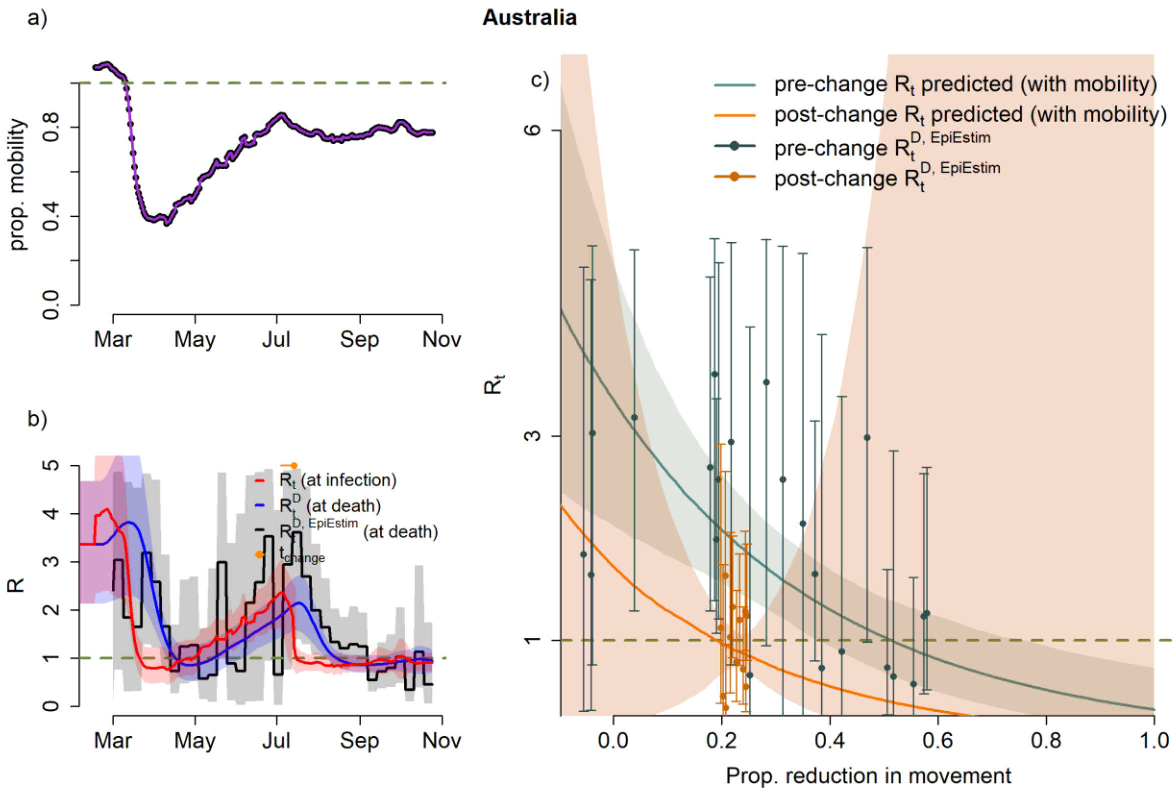
a)
Australia
prop. mobility
0.0
0.4
0.8
Mar
May
Jul
Sep
Nov
b)
R
0
1
2
3
4
5
R_t (at infection)
R_t^D (at death)
$R_t^{D, EpiEstim}$ (at death)
t_{change}
c)
R_t
1
3
6
pre-change R_t predicted (with mobility)
post-change R_t predicted (with mobility)
pre-change $R_t^{D, EpiEstim}$
post-change $R_t^{D, EpiEstim}$
0.0
0.2
0.4
0.6
0.8
1.0
Prop. reduction in movement

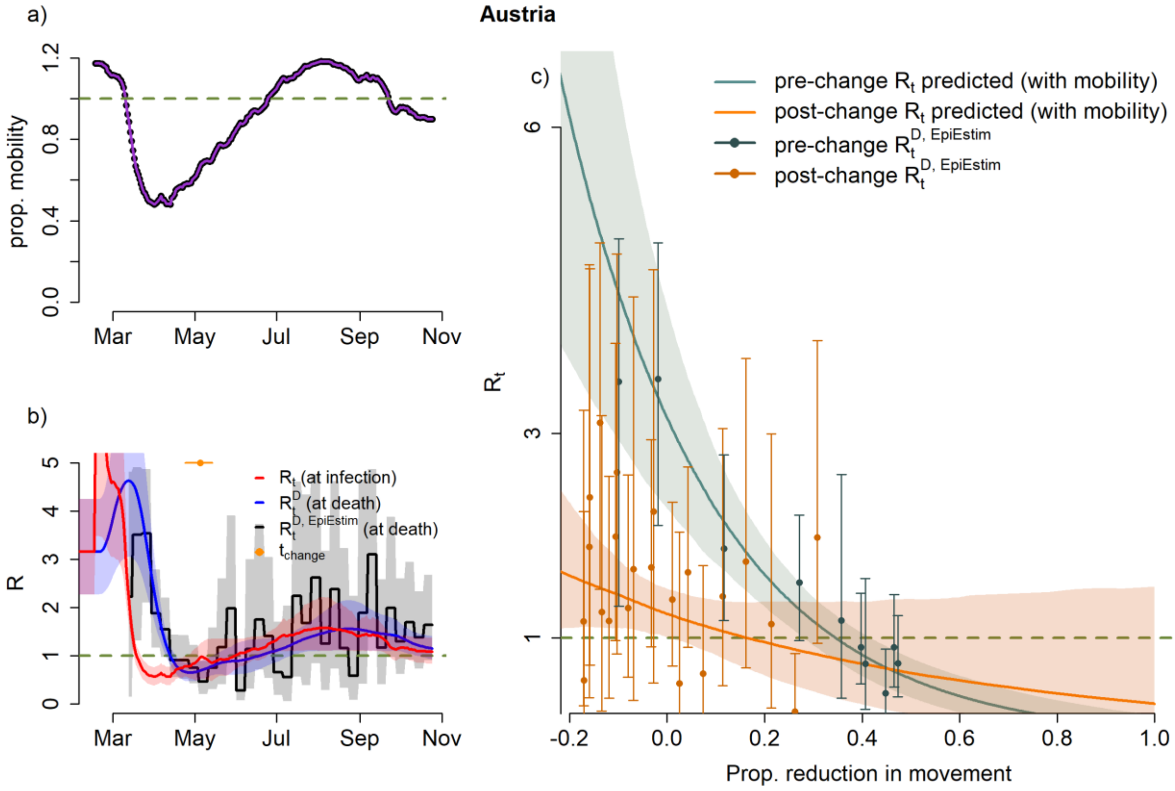
a)
Austria
prop. mobility
0.0
0.4
0.8
1.2
Mar
May
Jul
Sep
Nov
b)
R
0
1
2
3
4
5
R_t (at infection)
R_t^D (at death)
$R_t^{D, EpiEstim}$ (at death)
t_{change}
c)
R_t
1
3
6
pre-change R_t predicted (with mobility)
post-change R_t predicted (with mobility)
pre-change $R_t^{D, EpiEstim}$
post-change $R_t^{D, EpiEstim}$
-0.2
0.0
0.2
0.4
0.6
0.8
1.0
Prop. reduction in movement

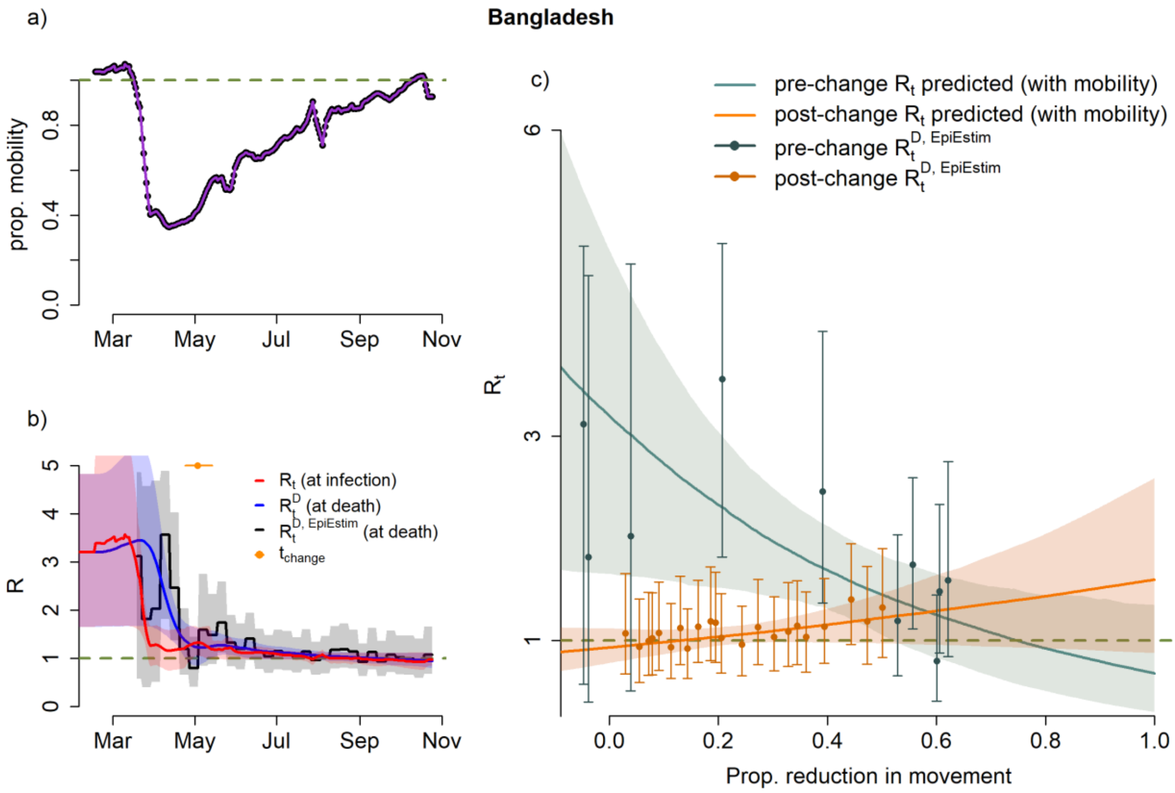
Bangladesh
a)
prop. mobility
b)
R
R_t (at infection)
R_t^D (at death)
R_t^{D, EpiEstim} (at death)
t_change
c)
pre-change R_t predicted (with mobility)
post-change R_t predicted (with mobility)
pre-change R_t^{D, EpiEstim}
post-change R_t^{D, EpiEstim}
R_t
Prop. reduction in movement

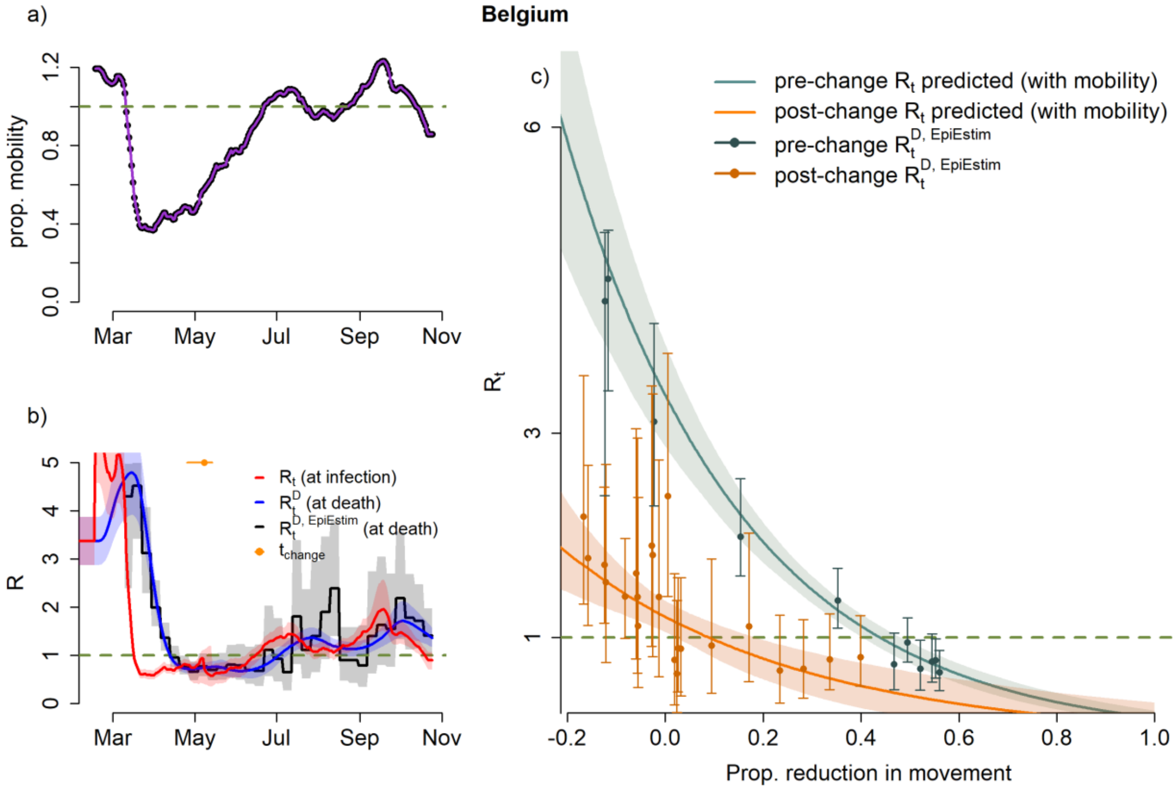
Belgium
a)
prop. mobility
b)
R
R_t (at infection)
R_t^D (at death)
R_t^{D, EpiEstim} (at death)
t_change
c)
pre-change R_t predicted (with mobility)
post-change R_t predicted (with mobility)
pre-change R_t^{D, EpiEstim}
post-change R_t^{D, EpiEstim}
R_t
Prop. reduction in movement

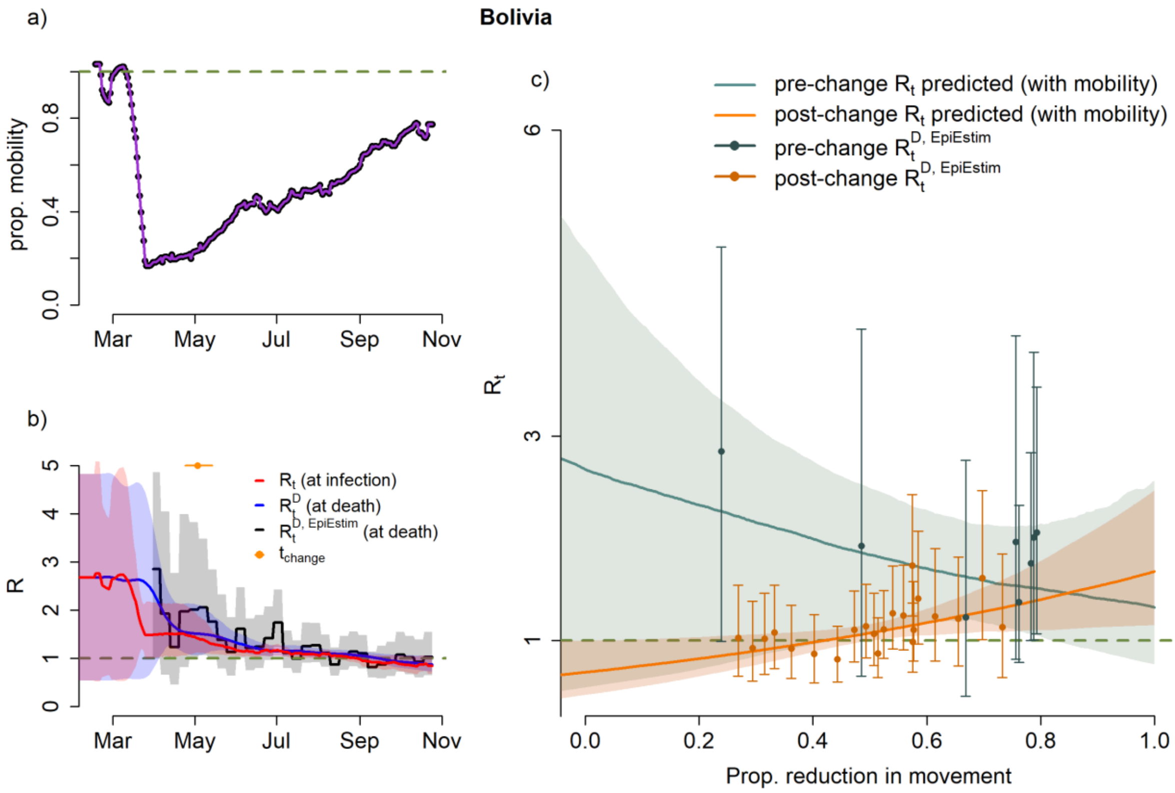
a)
Bolivia
prop. mobility
0.0
0.4
0.8
Mar
May
Jul
Sep
Nov
b)
R
0
1
2
3
4
5
R_t (at infection)
R_t^D (at death)
$R_t^{D, EpiEstim}$ (at death)
t_{change}
c)
pre-change R_t predicted (with mobility)
post-change R_t predicted (with mobility)
pre-change $R_t^{D, EpiEstim}$
post-change $R_t^{D, EpiEstim}$
R_t
1
3
6
0.0
0.2
0.4
0.6
0.8
1.0
Prop. reduction in movement

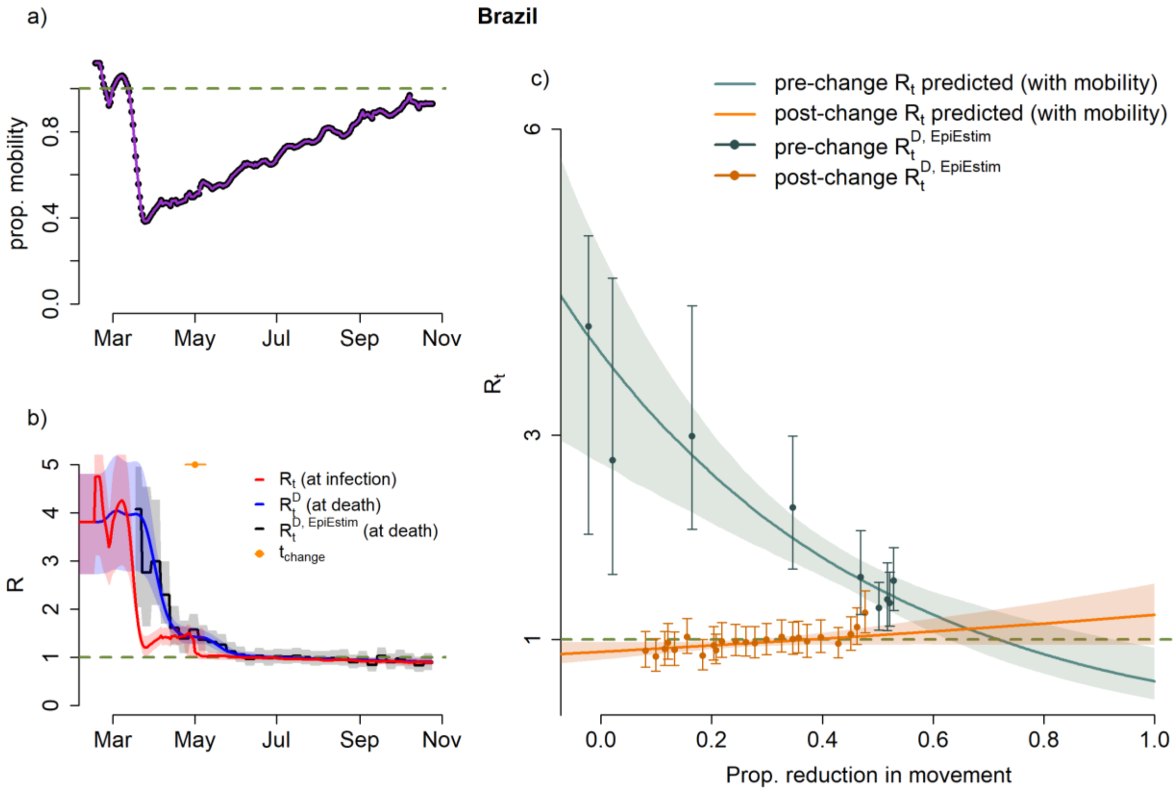
a)
Brazil
prop. mobility
0.0
0.4
0.8
Mar
May
Jul
Sep
Nov
b)
R
0
1
2
3
4
5
R_t (at infection)
R_t^D (at death)
$R_t^{D, EpiEstim}$ (at death)
t_{change}
c)
pre-change R_t predicted (with mobility)
post-change R_t predicted (with mobility)
pre-change $R_t^{D, EpiEstim}$
post-change $R_t^{D, EpiEstim}$
R_t
1
3
6
0.0
0.2
0.4
0.6
0.8
1.0
Prop. reduction in movement

Canada

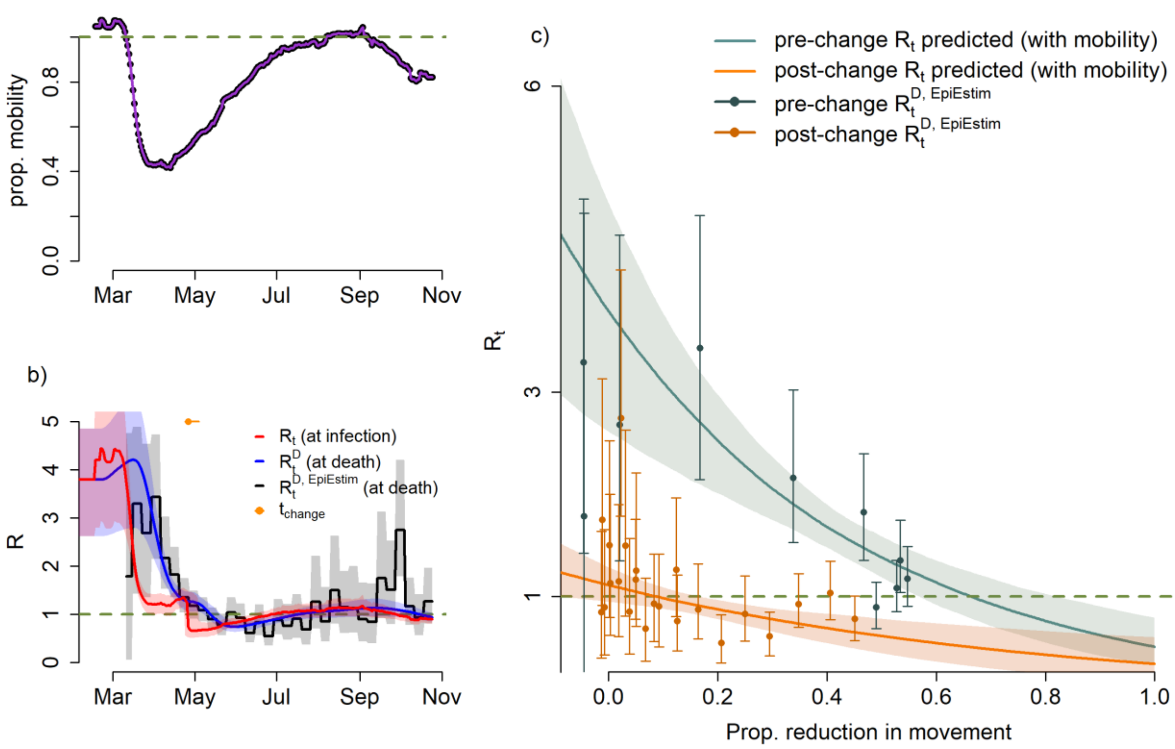

Chile

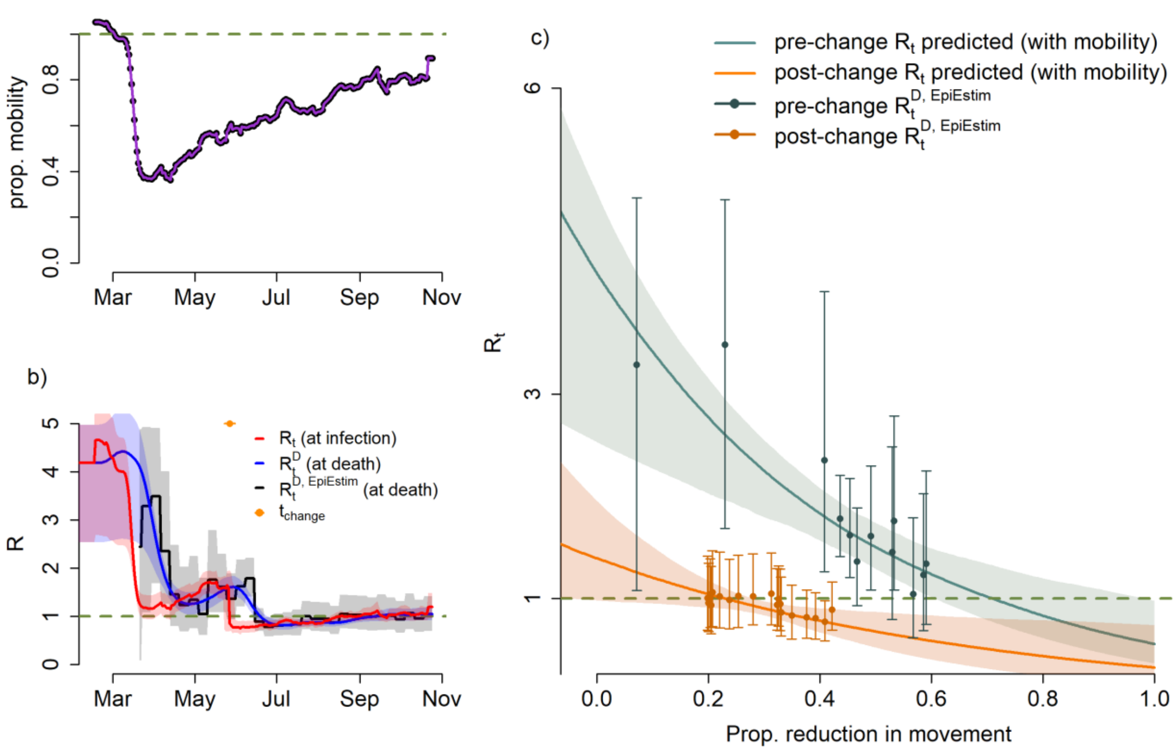

Colombia

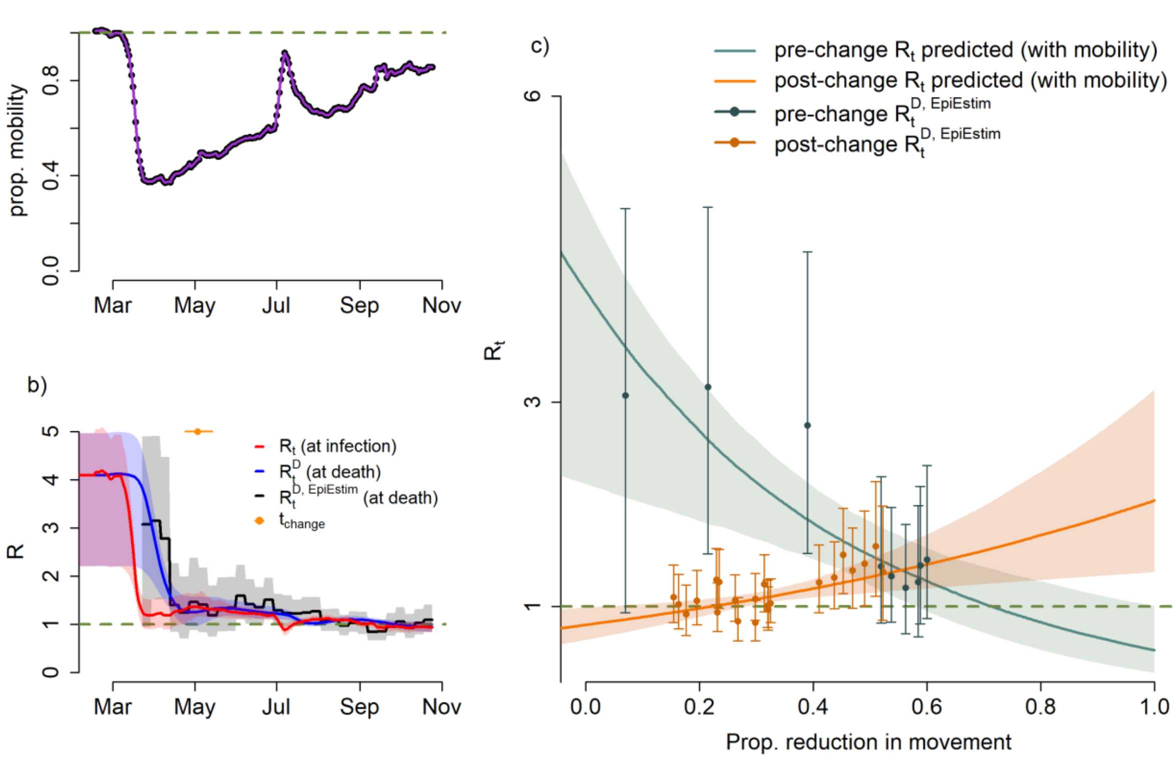

Costa Rica

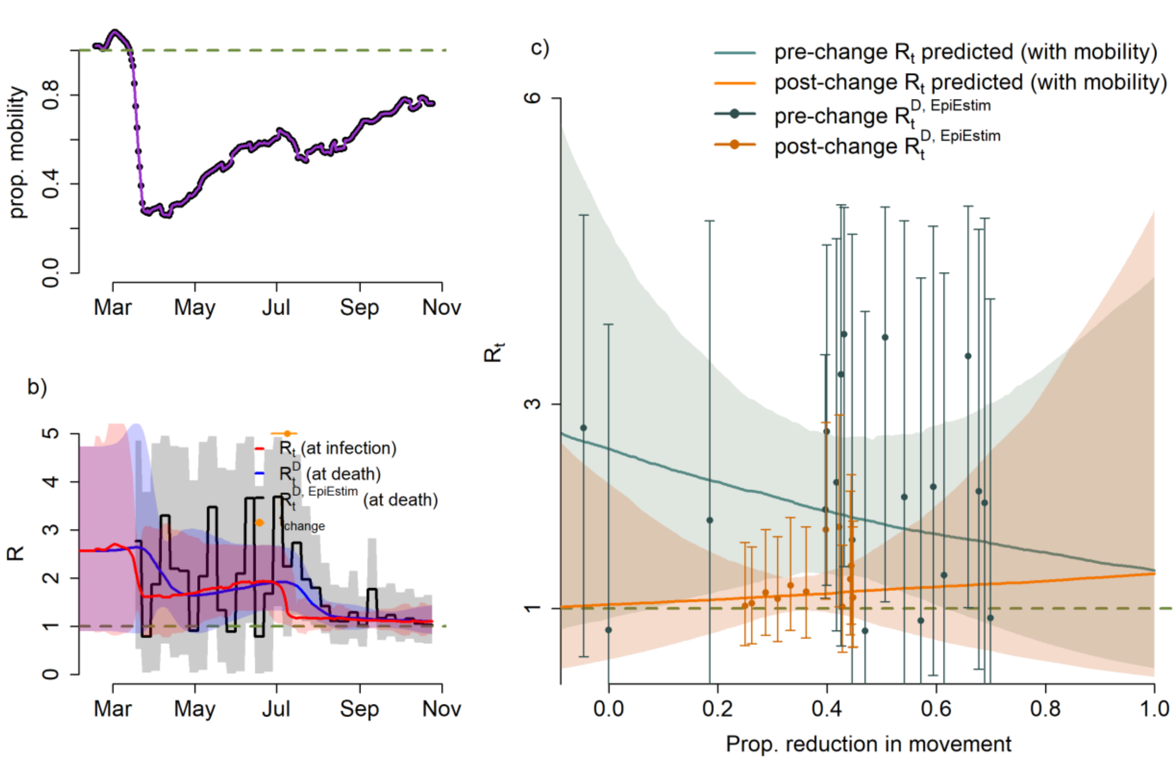

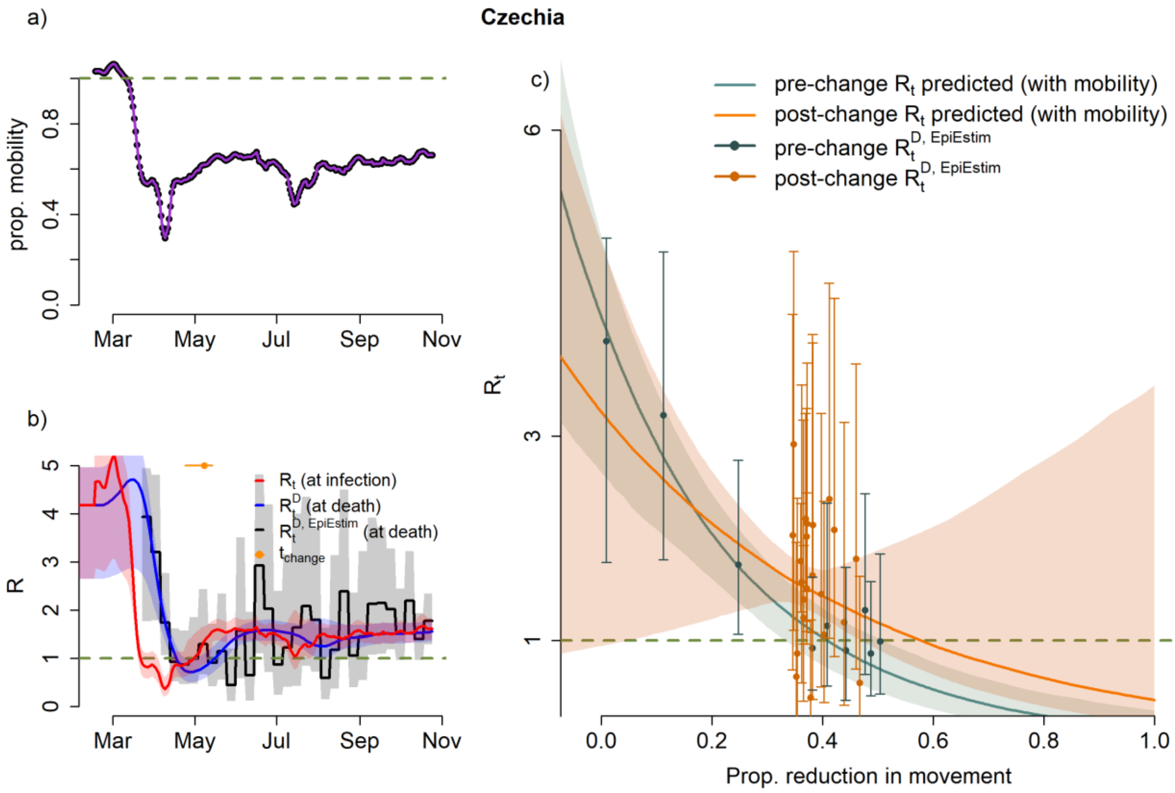
Czechia
a)
prop. mobility
0.0
0.4
0.8
Mar
May
Jul
Sep
Nov
b)
R
0
1
2
3
4
5
R_t (at infection)
R_t^D (at death)
$R_t^{D,\ EpiEstim}$ (at death)
t_{change}
c)
pre-change R_t predicted (with mobility)
post-change R_t predicted (with mobility)
pre-change $R_t^{D,\ EpiEstim}$
post-change $R_t^{D,\ EpiEstim}$
R_t
1
3
6
0.0
0.2
0.4
0.6
0.8
1.0
Prop. reduction in movement

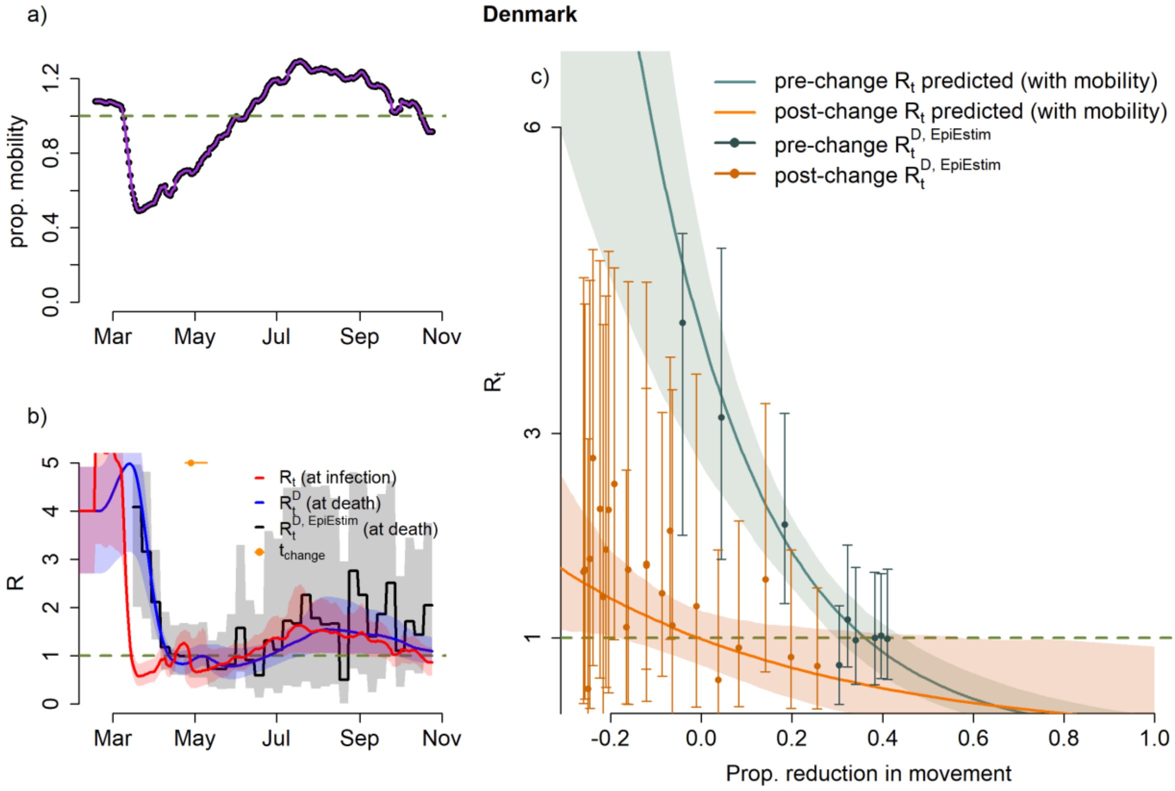
Denmark
a)
prop. mobility
0.0
0.4
0.8
1.2
Mar
May
Jul
Sep
Nov
b)
R
0
1
2
3
4
5
R_t (at infection)
R_t^D (at death)
$R_t^{D,\ EpiEstim}$ (at death)
t_{change}
c)
pre-change R_t predicted (with mobility)
post-change R_t predicted (with mobility)
pre-change $R_t^{D,\ EpiEstim}$
post-change $R_t^{D,\ EpiEstim}$
R_t
1
3
6
-0.2
0.0
0.2
0.4
0.6
0.8
1.0
Prop. reduction in movement

Dominican R.

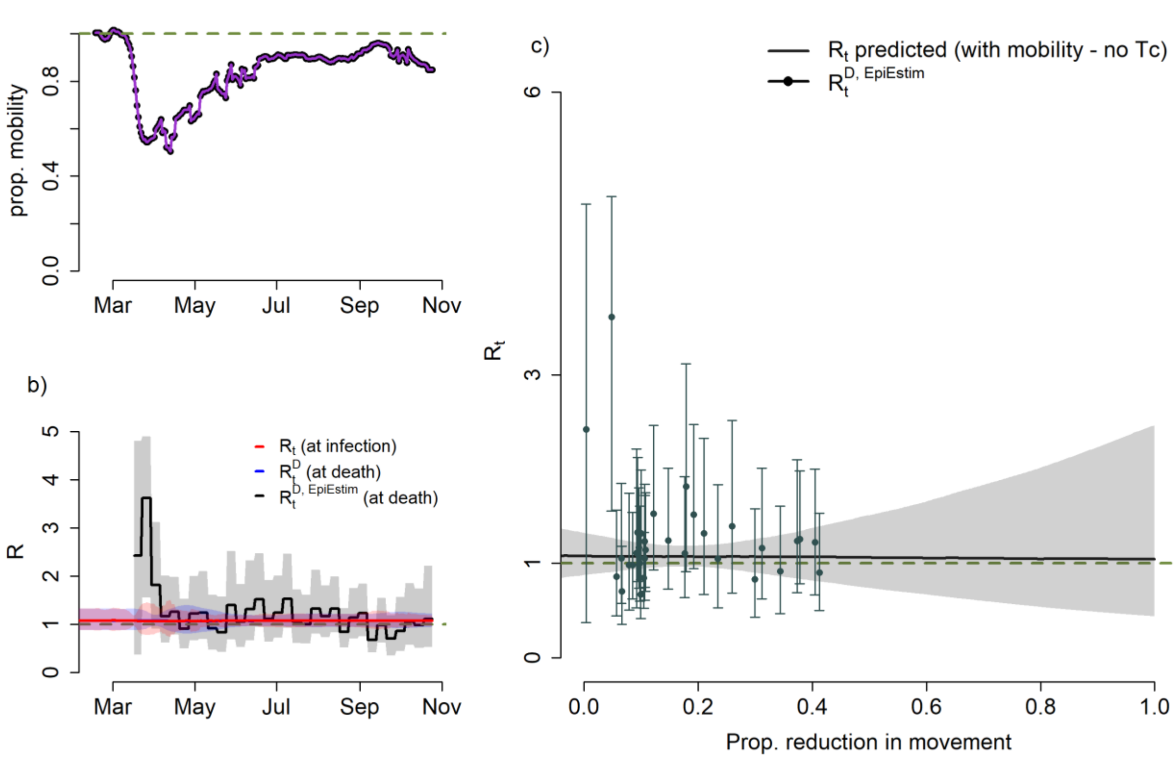

Ecuador

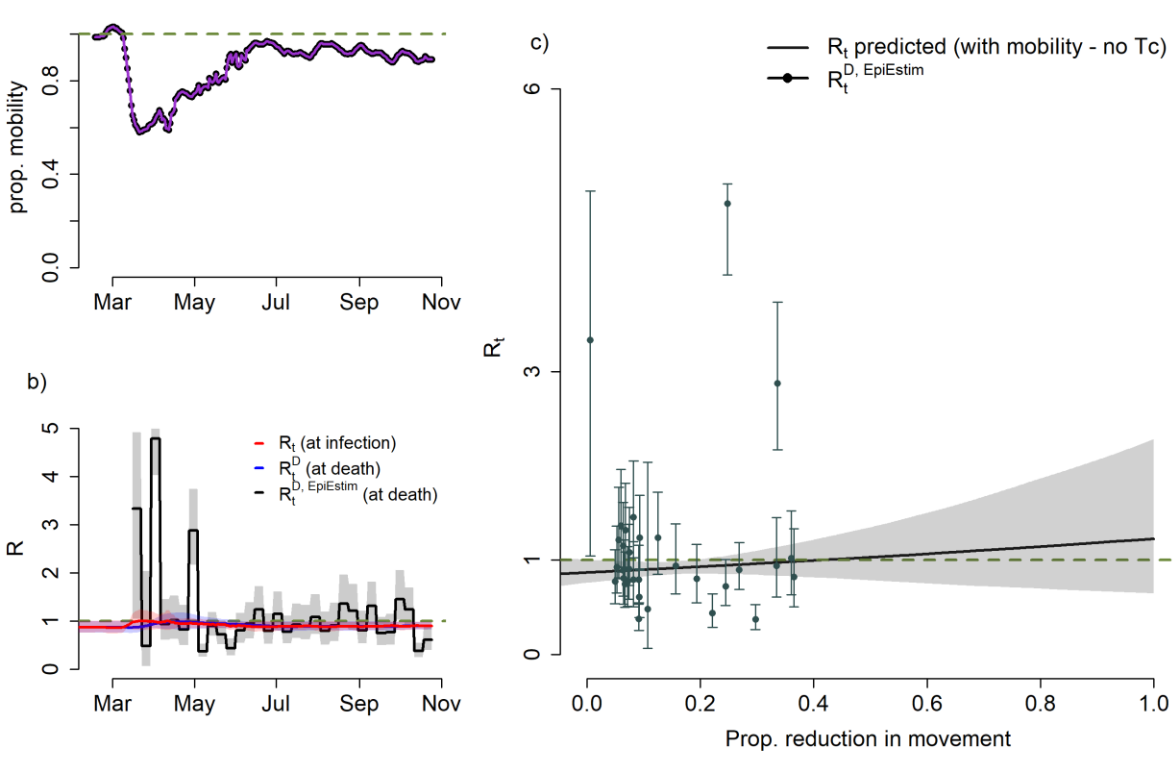

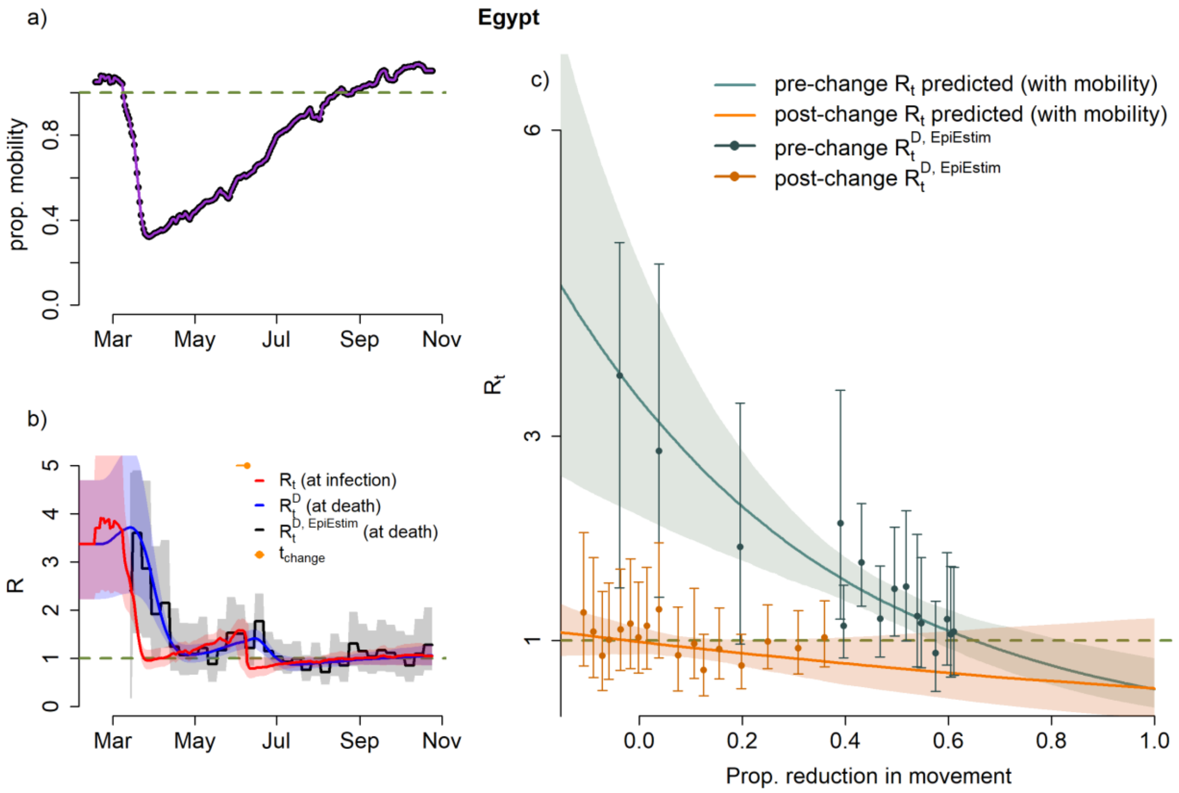
Egypt
a)
prop. mobility
0.0
0.4
0.8
Mar
May
Jul
Sep
Nov
b)
R
0
1
2
3
4
5
R_t (at infection)
R_t^D (at death)
$R_t^{D, EpiEstim}$ (at death)
t_{change}
c)
R_t
1
3
6
pre-change R_t predicted (with mobility)
post-change R_t predicted (with mobility)
pre-change $R_t^{D, EpiEstim}$
post-change $R_t^{D, EpiEstim}$
0.0
0.2
0.4
0.6
0.8
1.0
Prop. reduction in movement

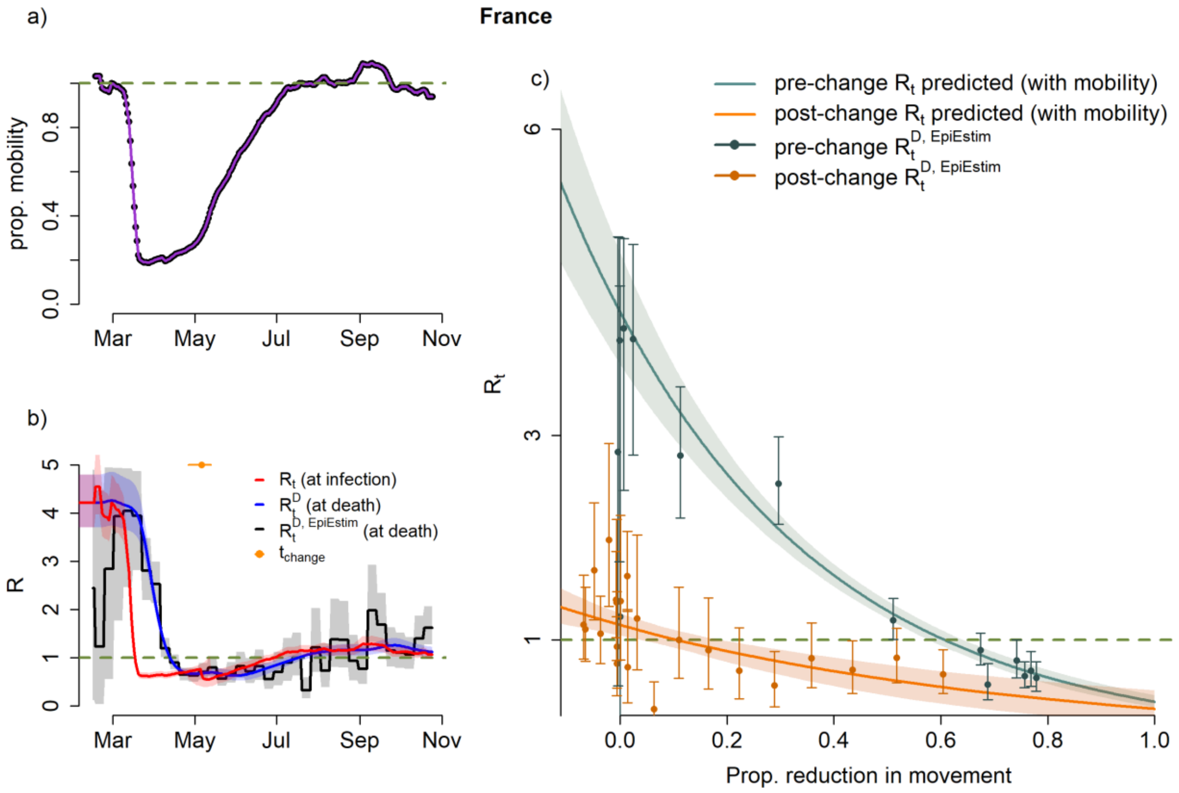
France
a)
prop. mobility
0.0
0.4
0.8
Mar
May
Jul
Sep
Nov
b)
R
0
1
2
3
4
5
R_t (at infection)
R_t^D (at death)
$R_t^{D, EpiEstim}$ (at death)
t_{change}
c)
R_t
1
3
6
pre-change R_t predicted (with mobility)
post-change R_t predicted (with mobility)
pre-change $R_t^{D, EpiEstim}$
post-change $R_t^{D, EpiEstim}$
0.0
0.2
0.4
0.6
0.8
1.0
Prop. reduction in movement

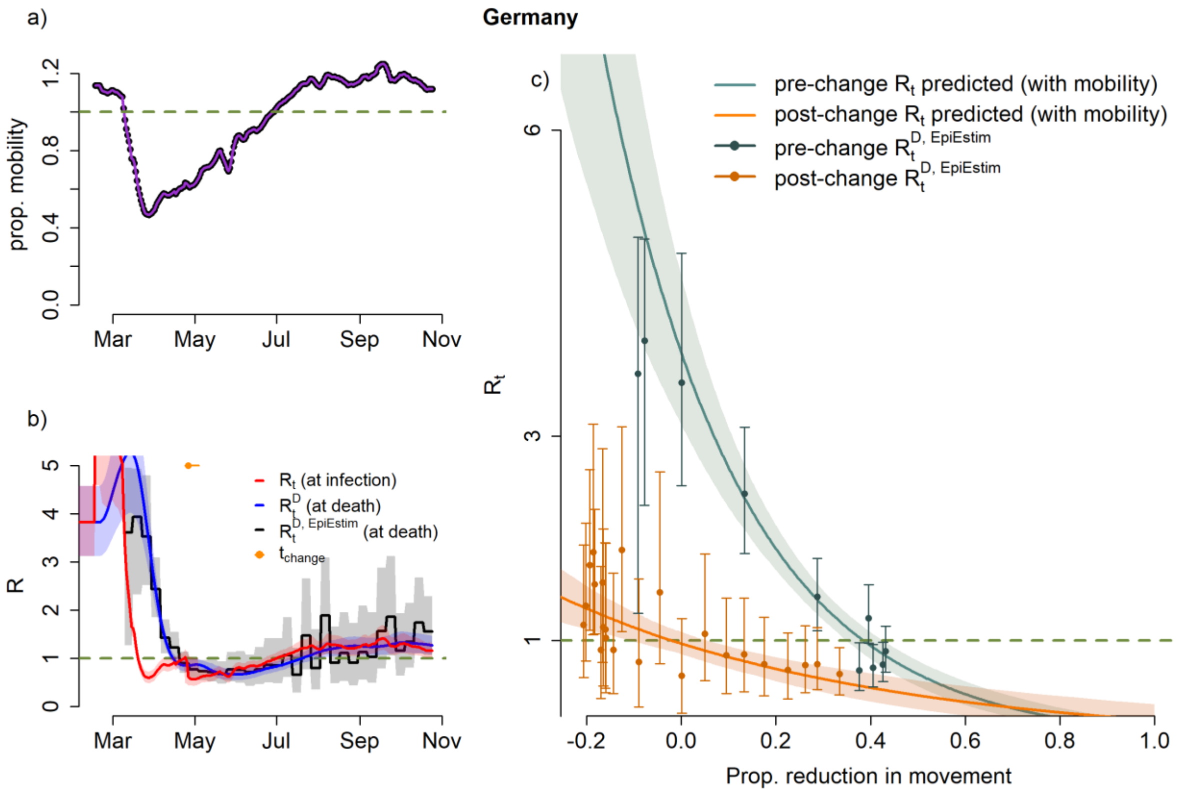
Germany
a)
prop. mobility
Mar May Jul Sep Nov
b)
R
R_t (at infection)
R_t^D (at death)
$R_t^{D,\ EpiEstim}$ (at death)
t_{change}
c)
R_t
pre-change R_t predicted (with mobility)
post-change R_t predicted (with mobility)
pre-change $R_t^{D,\ EpiEstim}$
post-change $R_t^{D,\ EpiEstim}$
Prop. reduction in movement

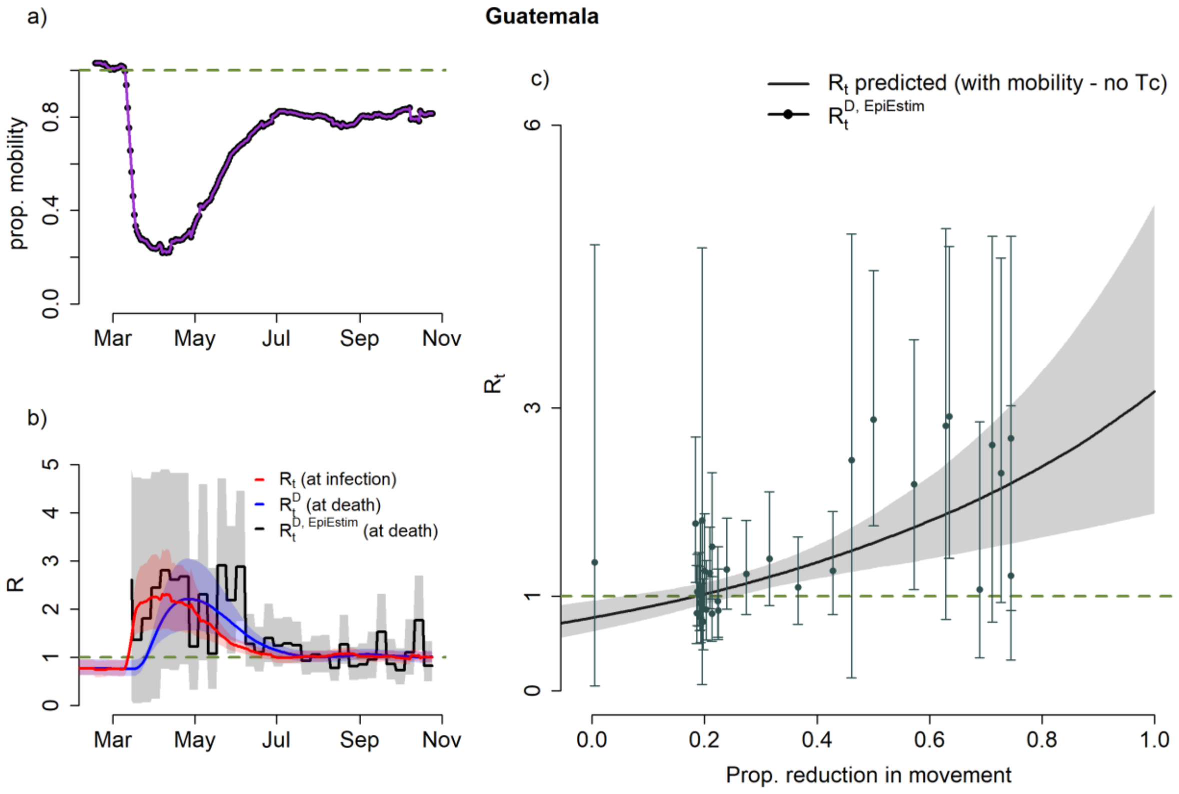
Guatemala
a)
prop. mobility
Mar May Jul Sep Nov
b)
R
R_t (at infection)
R_t^D (at death)
$R_t^{D,\ EpiEstim}$ (at death)
c)
R_t
R_t predicted (with mobility - no Tc)
$R_t^{D,\ EpiEstim}$
Prop. reduction in movement

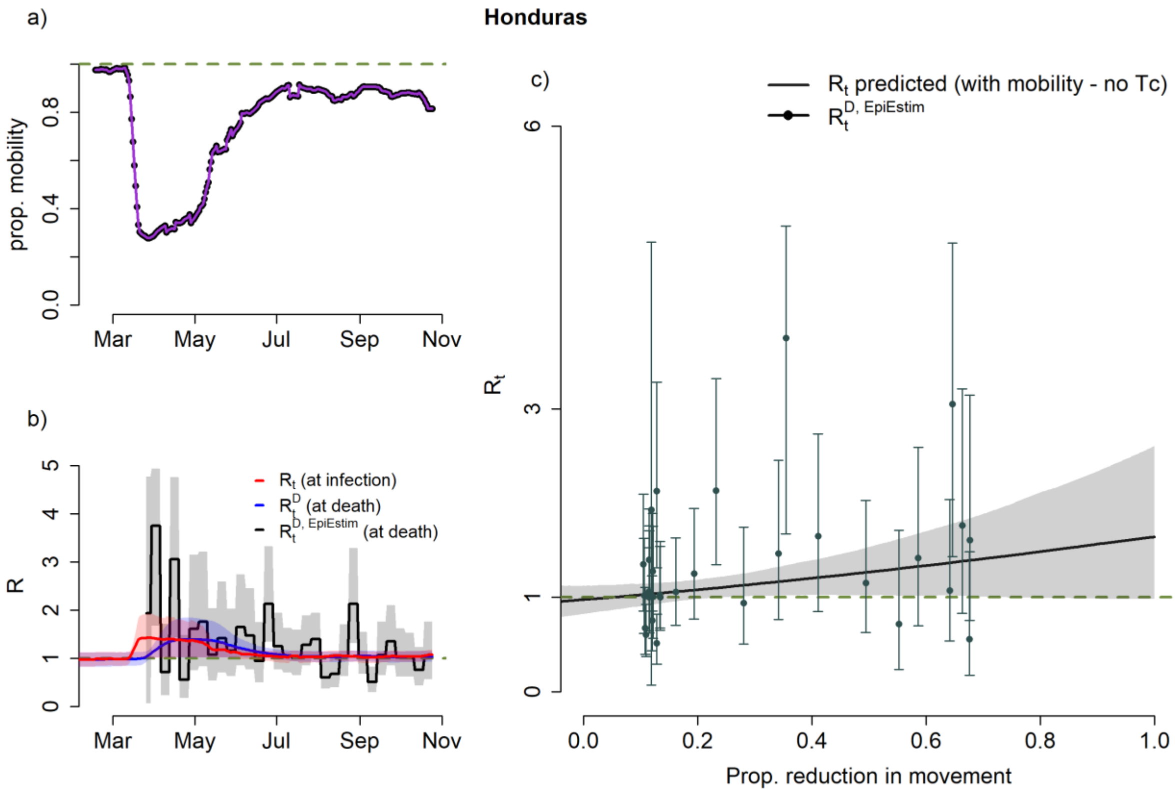
Honduras
a)
prop. mobility
Mar May Jul Sep Nov
b)
R
R_t (at infection)
R_t^D (at death)
$R_t^{D, EpiEstim}$ (at death)
c)
R_t predicted (with mobility - no Tc)
$R_t^{D, EpiEstim}$
R_t
Prop. reduction in movement

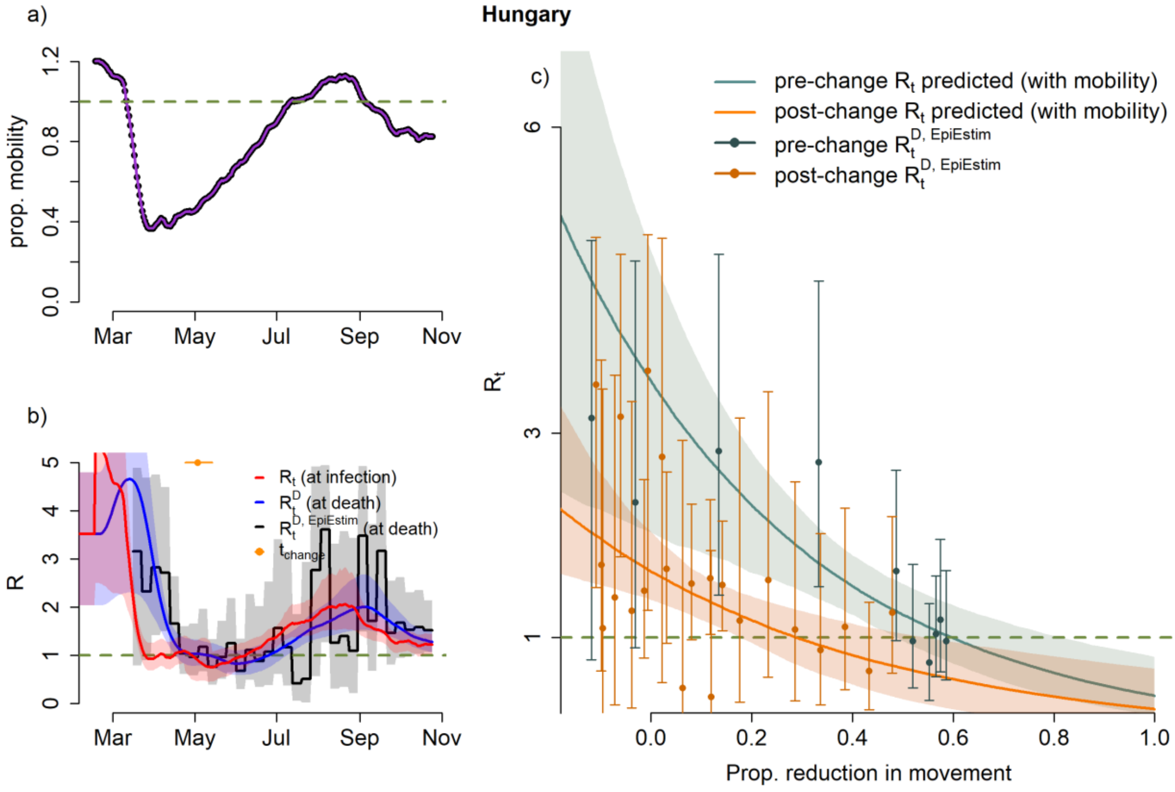
Hungary
a)
prop. mobility
Mar May Jul Sep Nov
b)
R
R_t (at infection)
R_t^D (at death)
$R_t^{D, EpiEstim}$ (at death)
t_{change}
c)
pre-change R_t predicted (with mobility)
post-change R_t predicted (with mobility)
pre-change $R_t^{D, EpiEstim}$
post-change $R_t^{D, EpiEstim}$
R_t
Prop. reduction in movement

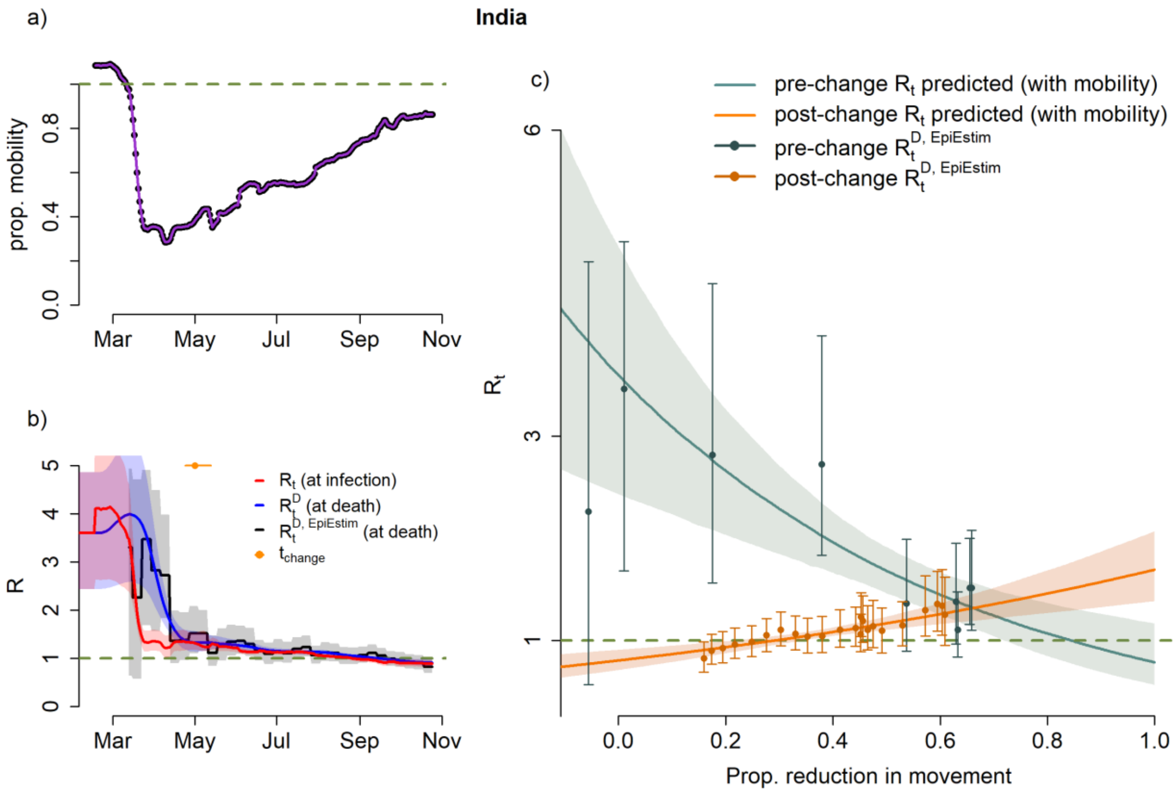
India
a)
prop. mobility
b)
R
R_t (at infection)
R_t^D (at death)
$R_t^{D, EpiEstim}$ (at death)
t_{change}
Mar
May
Jul
Sep
Nov
c)
pre-change R_t predicted (with mobility)
post-change R_t predicted (with mobility)
pre-change $R_t^{D, EpiEstim}$
post-change $R_t^{D, EpiEstim}$
R_t
Prop. reduction in movement

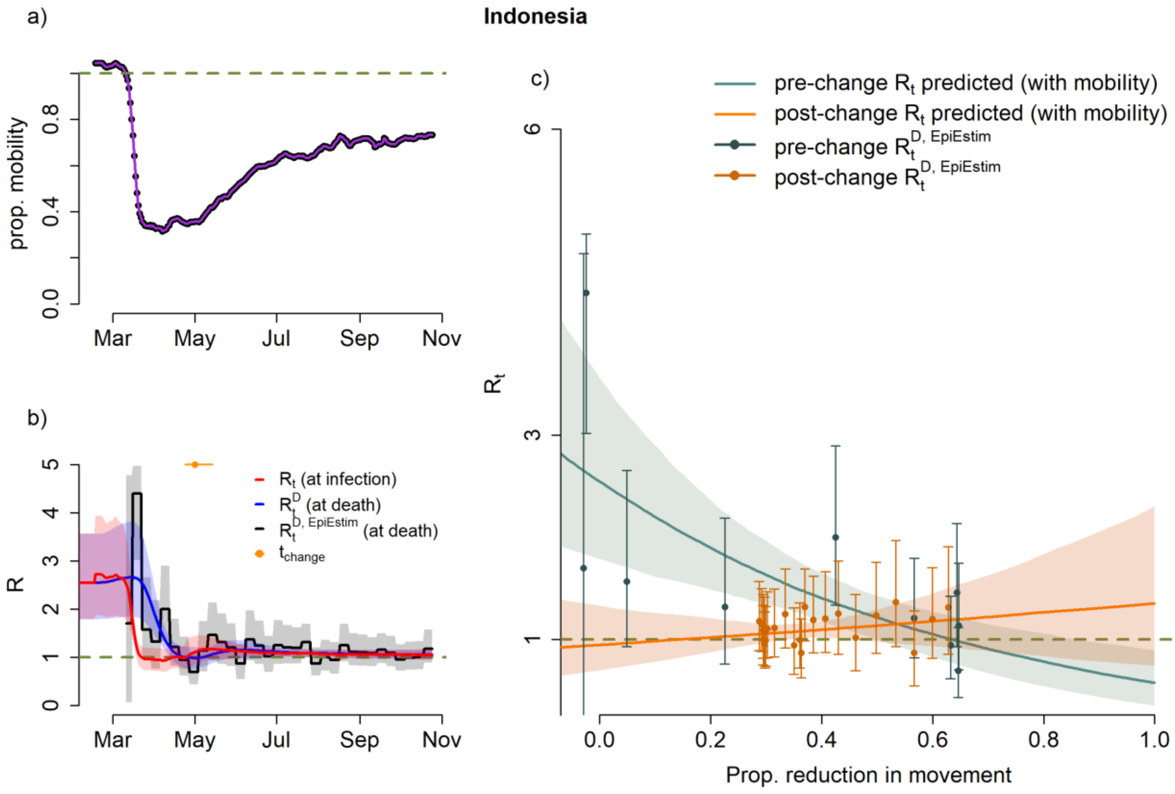
Indonesia
a)
prop. mobility
b)
R
R_t (at infection)
R_t^D (at death)
$R_t^{D, EpiEstim}$ (at death)
t_{change}
Mar
May
Jul
Sep
Nov
c)
pre-change R_t predicted (with mobility)
post-change R_t predicted (with mobility)
pre-change $R_t^{D, EpiEstim}$
post-change $R_t^{D, EpiEstim}$
R_t
Prop. reduction in movement

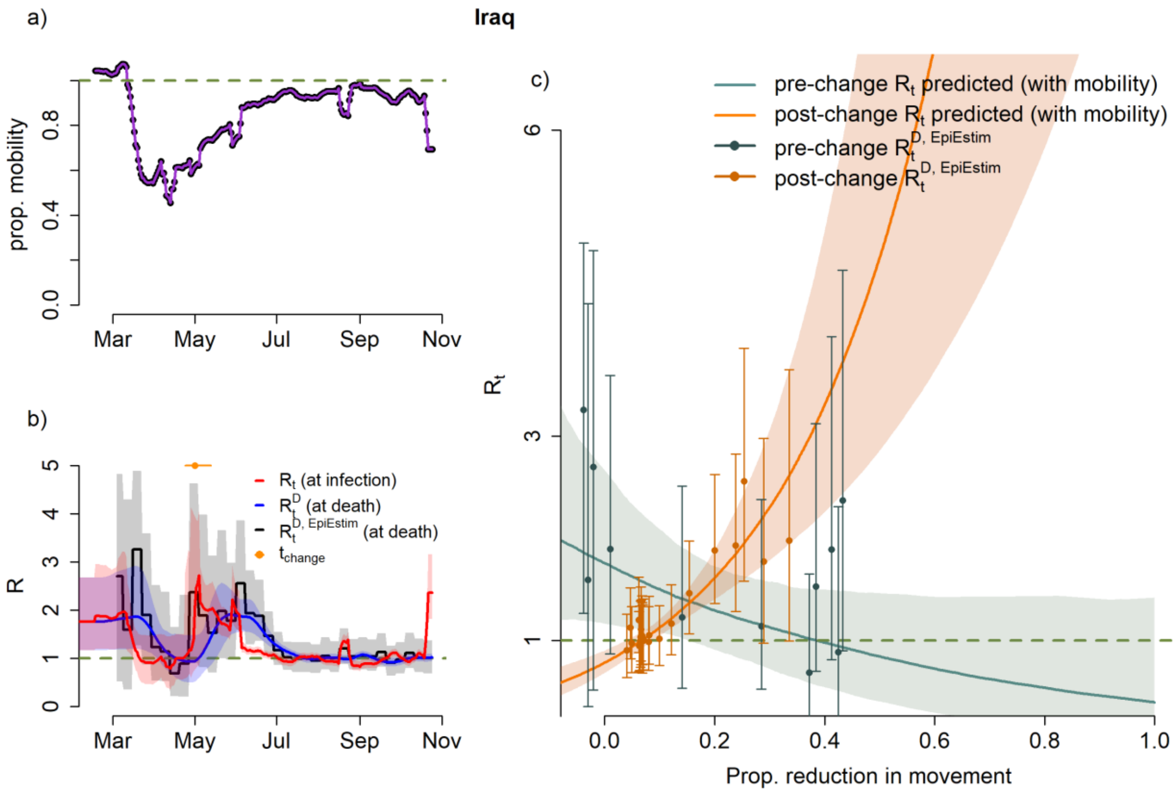
Iraq
a)
prop. mobility
0.0
0.4
0.8
Mar
May
Jul
Sep
Nov
b)
R
R_t (at infection)
R_t^D (at death)
$R_t^{D,\ EpiEstim}$ (at death)
t_{change}
c)
pre-change R_t predicted (with mobility)
post-change R_t predicted (with mobility)
pre-change $R_t^{D,\ EpiEstim}$
post-change $R_t^{D,\ EpiEstim}$
R_t
Prop. reduction in movement

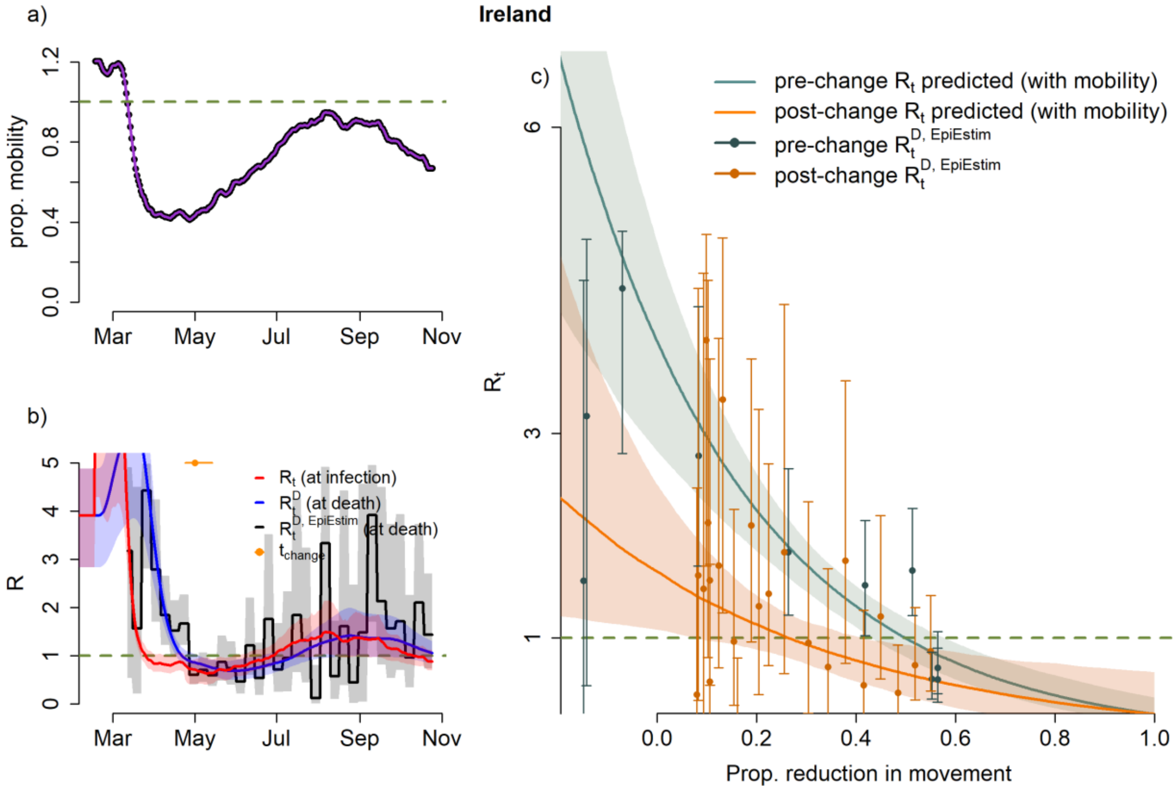
Ireland
a)
prop. mobility
0.0
0.4
0.8
1.2
Mar
May
Jul
Sep
Nov
b)
R
R_t (at infection)
R_t^D (at death)
$R_t^{D,\ EpiEstim}$ (at death)
t_{change}
c)
pre-change R_t predicted (with mobility)
post-change R_t predicted (with mobility)
pre-change $R_t^{D,\ EpiEstim}$
post-change $R_t^{D,\ EpiEstim}$
R_t
Prop. reduction in movement

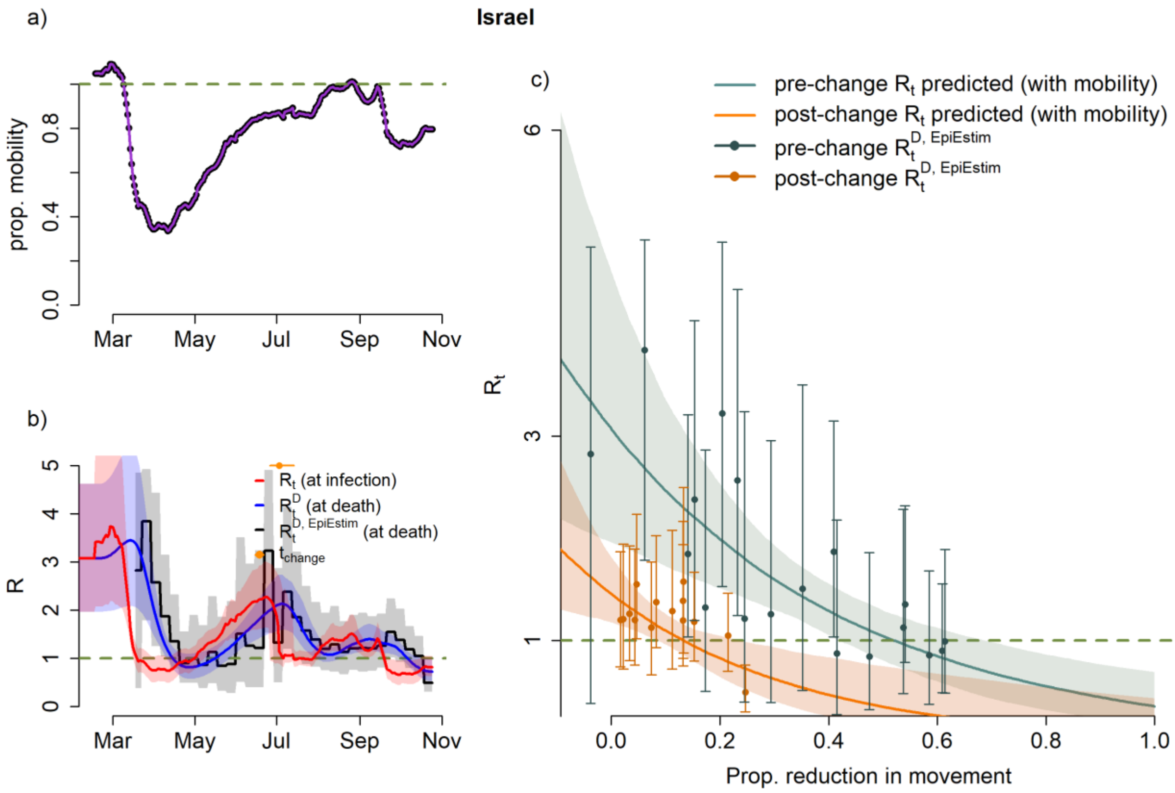
Israel
a)
prop. mobility
0.0
0.4
0.8
Mar
May
Jul
Sep
Nov
b)
R
0
1
2
3
4
5
Rt (at infection)
RtD (at death)
RtD, EpiEstim (at death)
tchange
c)
Rt
1
3
6
pre-change Rt predicted (with mobility)
post-change Rt predicted (with mobility)
pre-change RtD, EpiEstim
post-change RtD, EpiEstim
0.0
0.2
0.4
0.6
0.8
1.0
Prop. reduction in movement

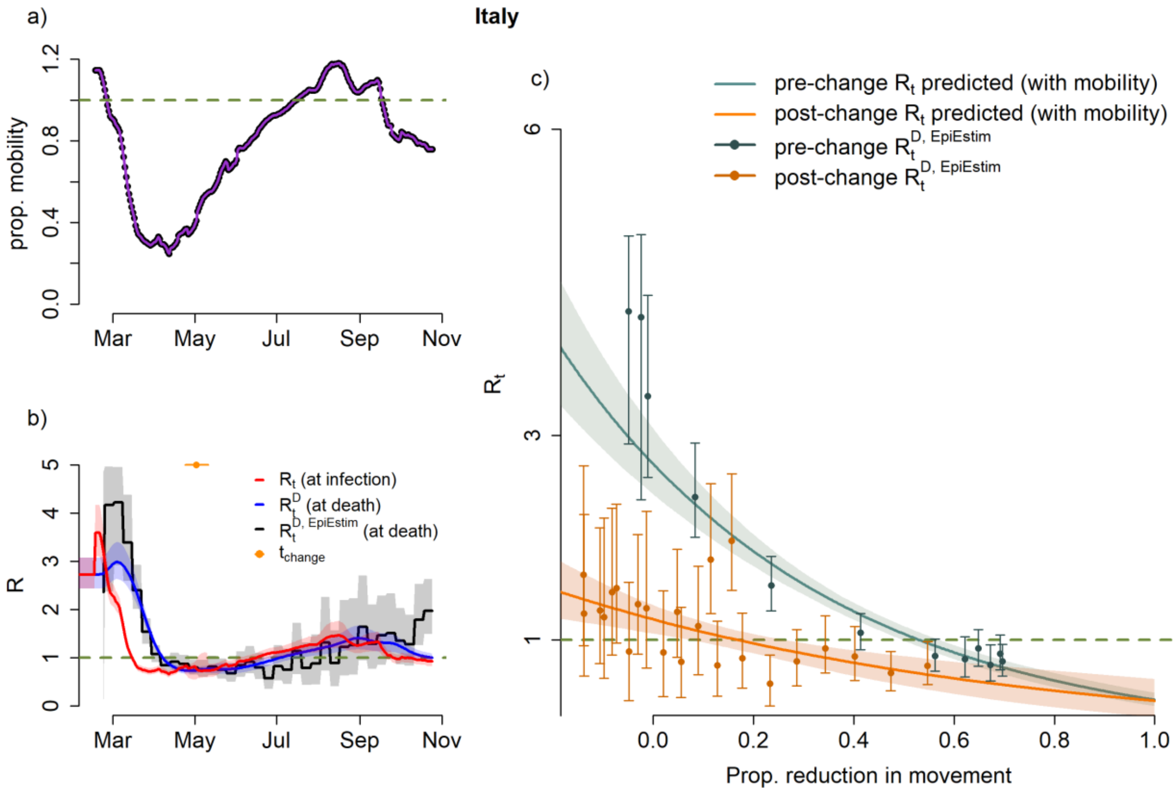
Italy
a)
prop. mobility
0.0
0.4
0.8
1.2
Mar
May
Jul
Sep
Nov
b)
R
0
1
2
3
4
5
Rt (at infection)
RtD (at death)
RtD, EpiEstim (at death)
tchange
c)
Rt
1
3
6
pre-change Rt predicted (with mobility)
post-change Rt predicted (with mobility)
pre-change RtD, EpiEstim
post-change RtD, EpiEstim
0.0
0.2
0.4
0.6
0.8
1.0
Prop. reduction in movement

Japan

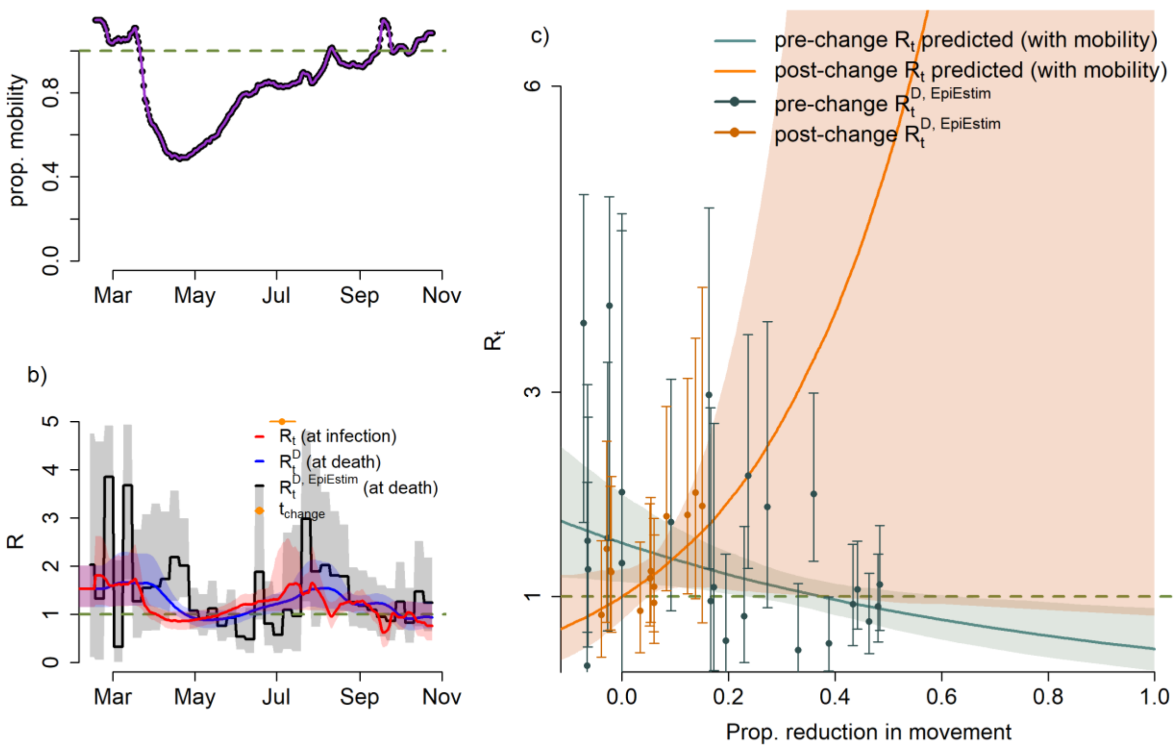

a)
prop. mobility
0.0
0.4
0.8
Mar
May
Jul
Sep
Nov
b)
R
0
1
2
3
4
5
Rt (at infection)
RtD (at death)
RtD, EpiEstim (at death)
tchange
c)
pre-change Rt predicted (with mobility)
post-change Rt predicted (with mobility)
pre-change RtD, EpiEstim
post-change RtD, EpiEstim
Rt
1
3
6
0.0
0.2
0.4
0.6
0.8
1.0
Prop. reduction in movement

Libya

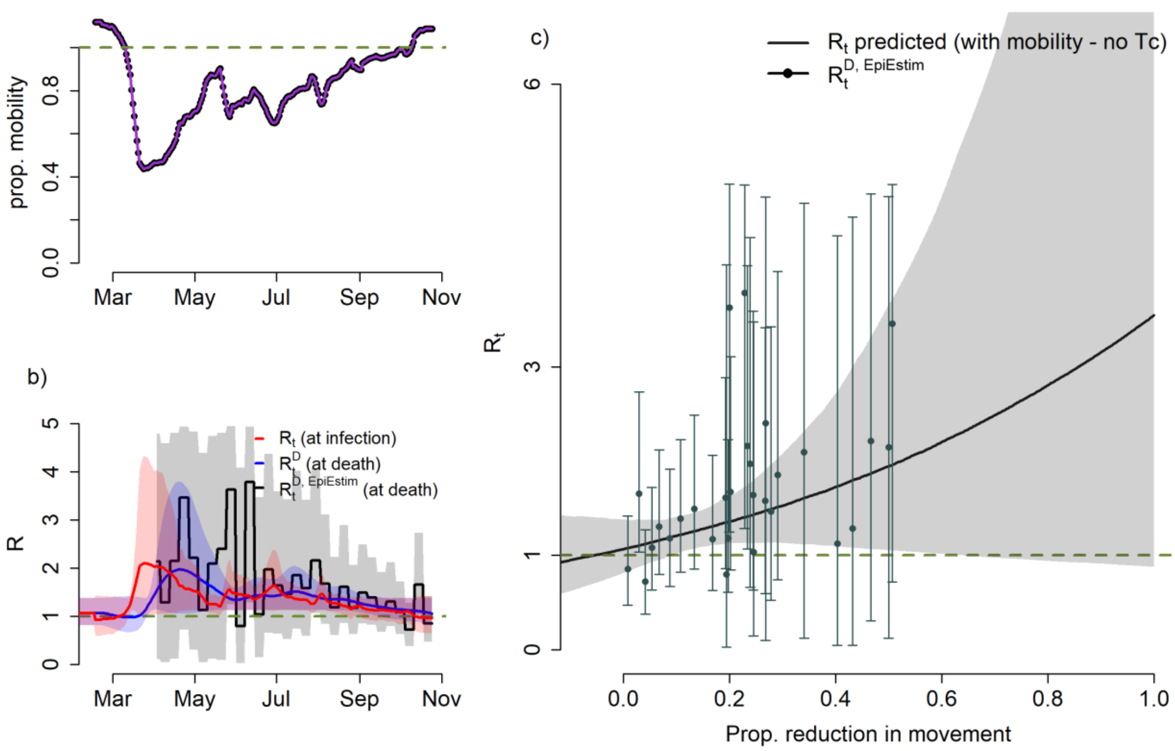

a)
prop. mobility
0.0
0.4
0.8
Mar
May
Jul
Sep
Nov
b)
R
0
1
2
3
4
5
Rt (at infection)
RtD (at death)
RtD, EpiEstim (at death)
c)
Rt predicted (with mobility - no Tc)
RtD, EpiEstim
Rt
0
1
3
6
0.0
0.2
0.4
0.6
0.8
1.0
Prop. reduction in movement

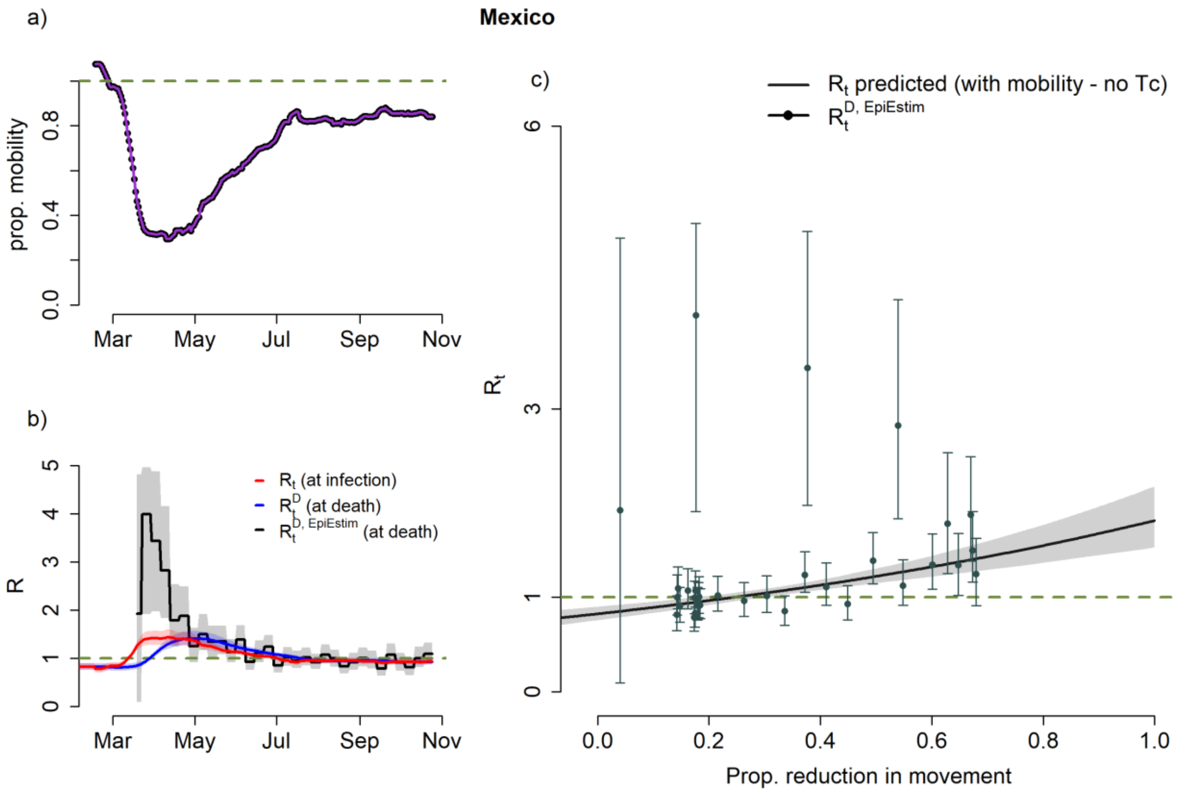

Mexico
a)
prop. mobility
0.0
0.4
0.8
Mar
May
Jul
Sep
Nov
b)
R
Rt (at infection)
RtD (at death)
RtD, EpiEstim (at death)
c)
Rt predicted (with mobility - no Tc)
RtD, EpiEstim
Rt
Prop. reduction in movement
0.0
0.2
0.4
0.6
0.8
1.0

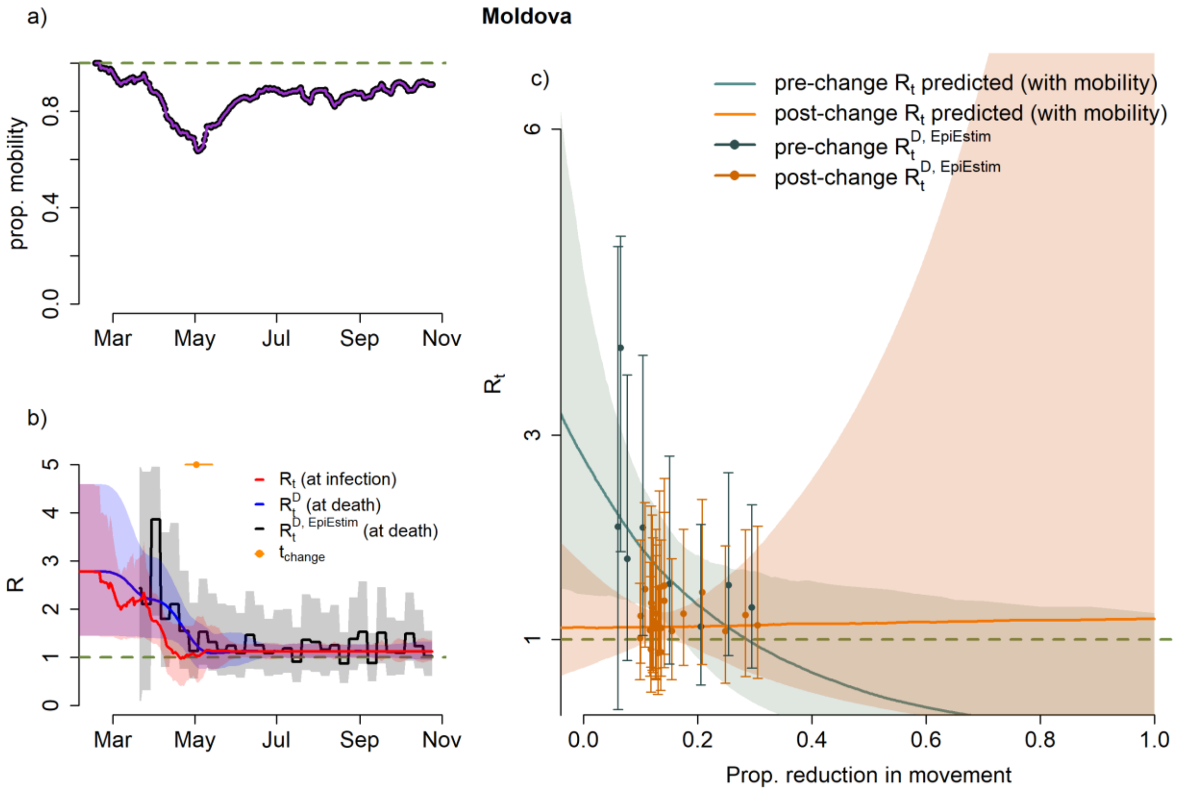

Moldova
a)
prop. mobility
0.0
0.4
0.8
Mar
May
Jul
Sep
Nov
b)
R
Rt (at infection)
RtD (at death)
RtD, EpiEstim (at death)
tchange
c)
pre-change Rt predicted (with mobility)
post-change Rt predicted (with mobility)
pre-change RtD, EpiEstim
post-change RtD, EpiEstim
Rt
Prop. reduction in movement
0.0
0.2
0.4
0.6
0.8
1.0

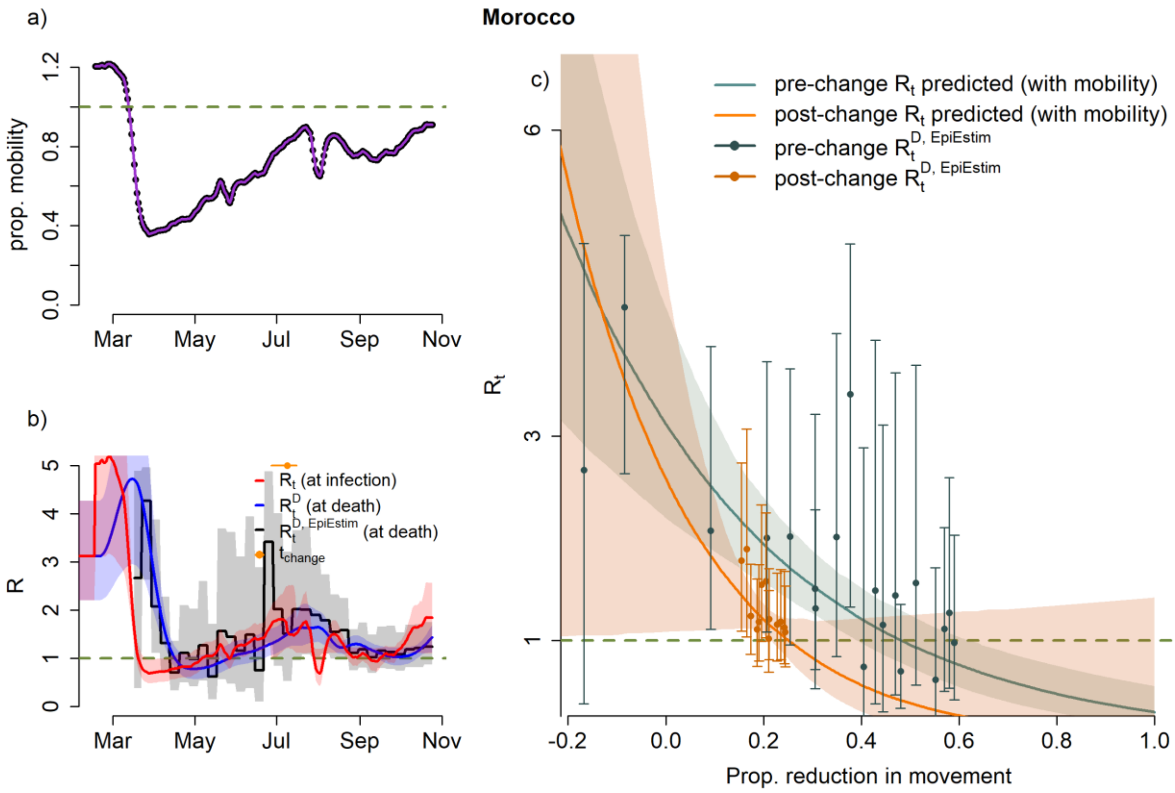
Morocco
a)
prop. mobility
0.0
0.4
0.8
1.2
Mar
May
Jul
Sep
Nov
b)
R
Rt (at infection)
RD t (at death)
RtD, EpiEstim (at death)
tchange
c)
pre-change Rt predicted (with mobility)
post-change Rt predicted (with mobility)
pre-change RtD, EpiEstim
post-change RtD, EpiEstim
Rt
Prop. reduction in movement

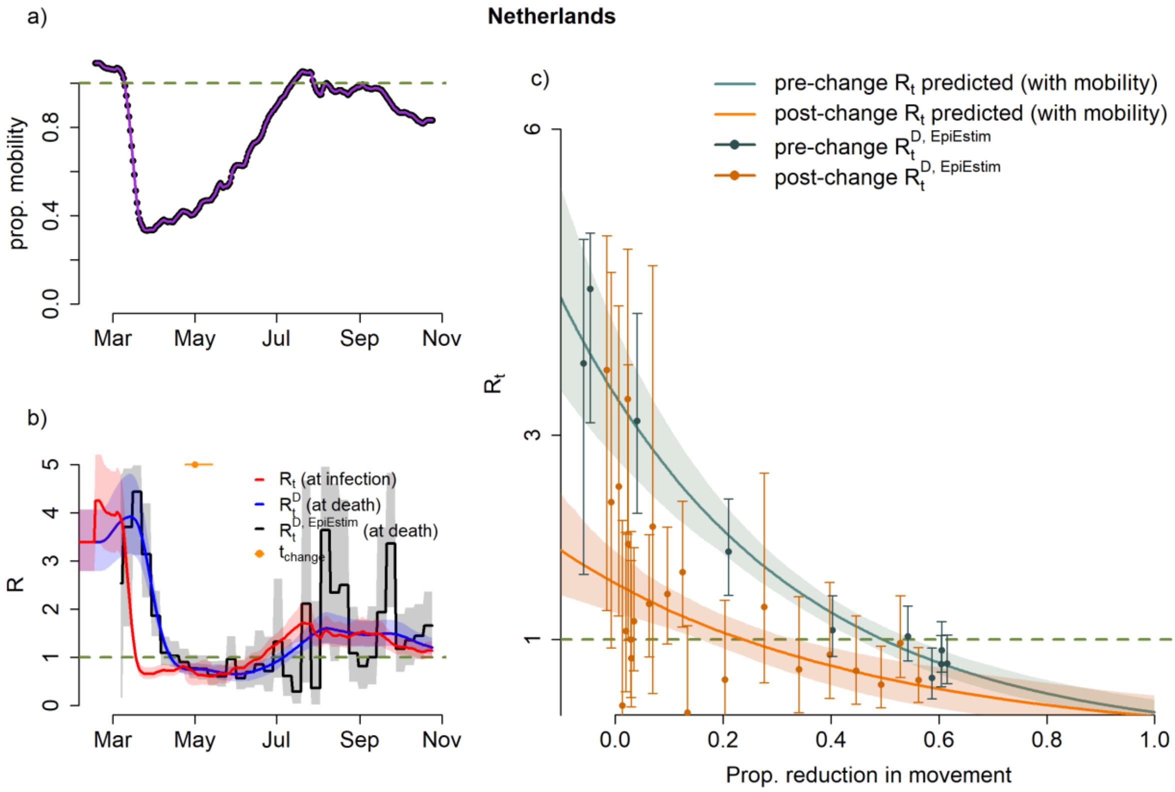
Netherlands
a)
prop. mobility
0.0
0.4
0.8
Mar
May
Jul
Sep
Nov
b)
R
Rt (at infection)
RD t (at death)
RtD, EpiEstim (at death)
tchange
c)
pre-change Rt predicted (with mobility)
post-change Rt predicted (with mobility)
pre-change RtD, EpiEstim
post-change RtD, EpiEstim
Rt
Prop. reduction in movement

Pakistan

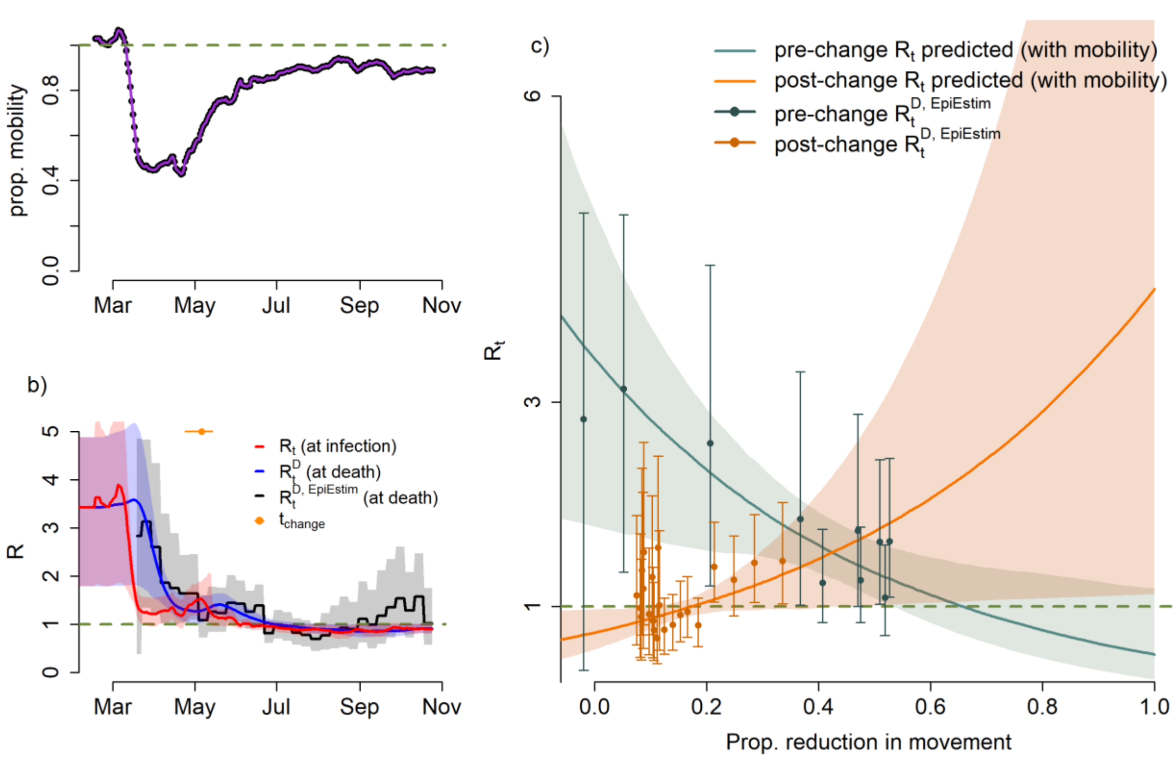

Panama

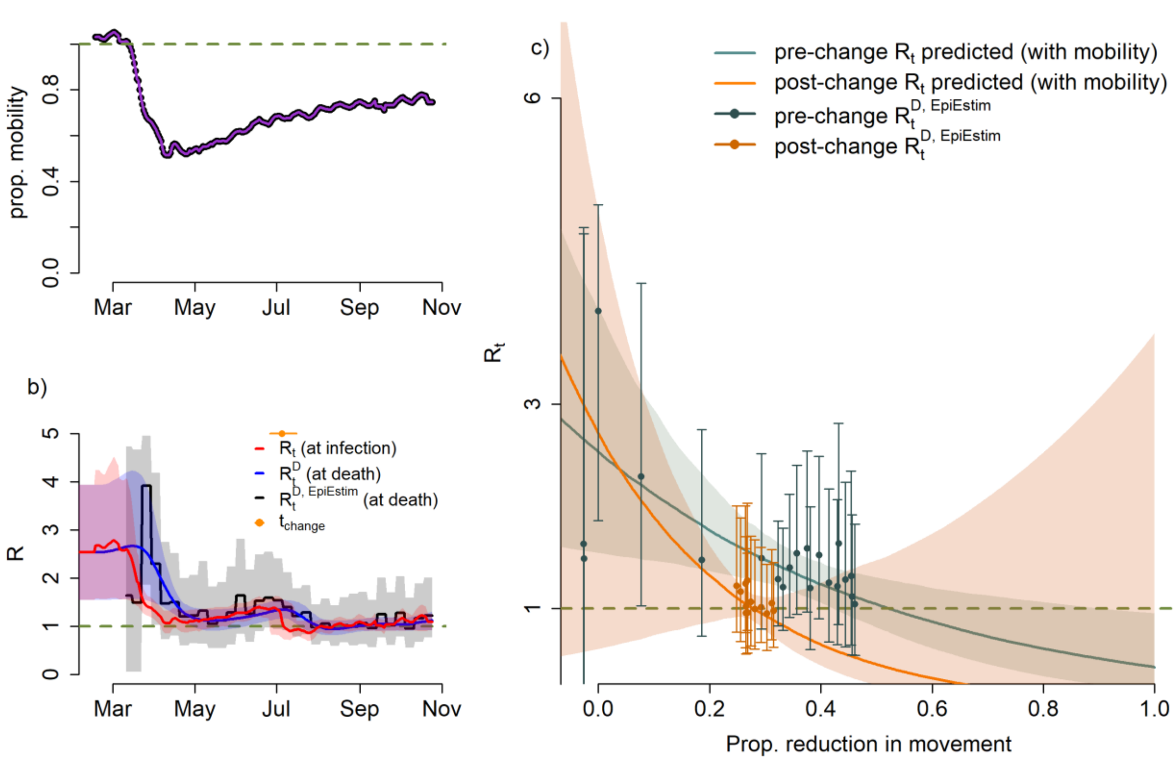

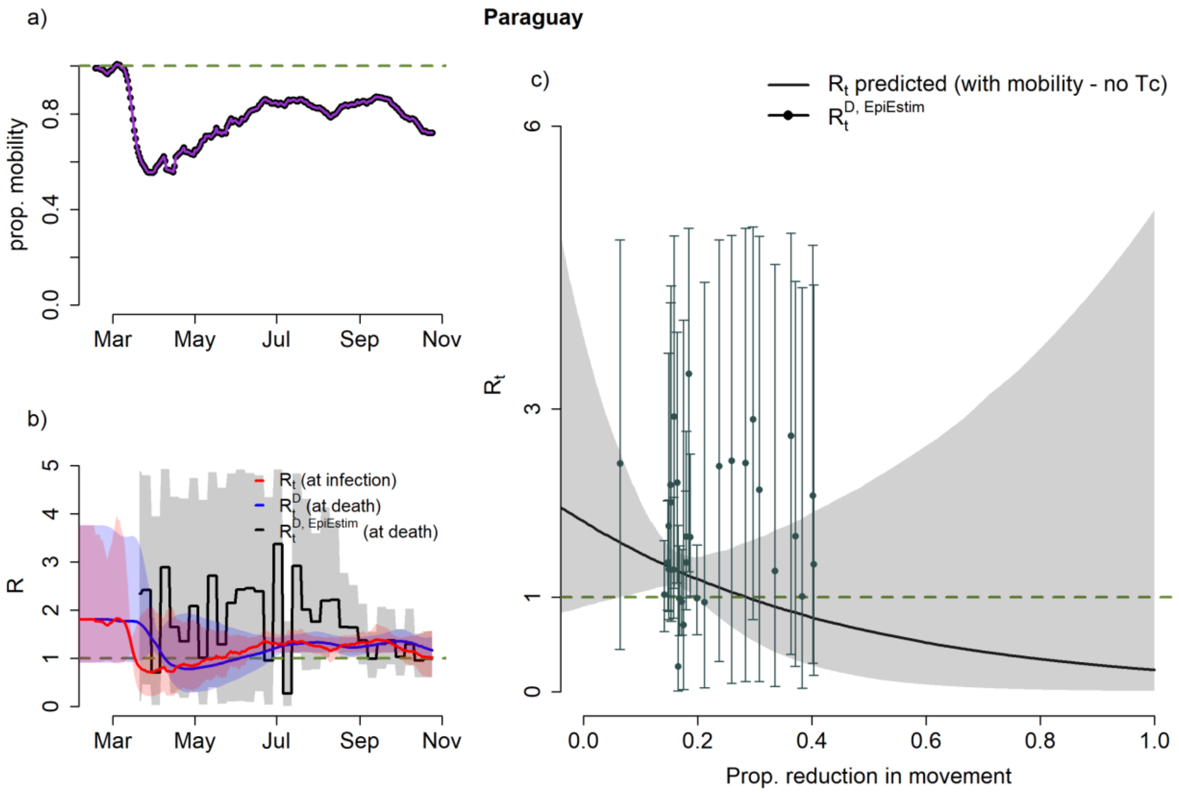
Paraguay
a)
prop. mobility
0.0
0.4
0.8
Mar
May
Jul
Sep
Nov
b)
R
0
1
2
3
4
5
R_t (at infection)
R_t^D (at death)
R_t^{D, EpiEstim} (at death)
c)
R_t predicted (with mobility - no Tc)
R_t^{D, EpiEstim}
R_t
0
1
3
6
0.0
0.2
0.4
0.6
0.8
1.0
Prop. reduction in movement

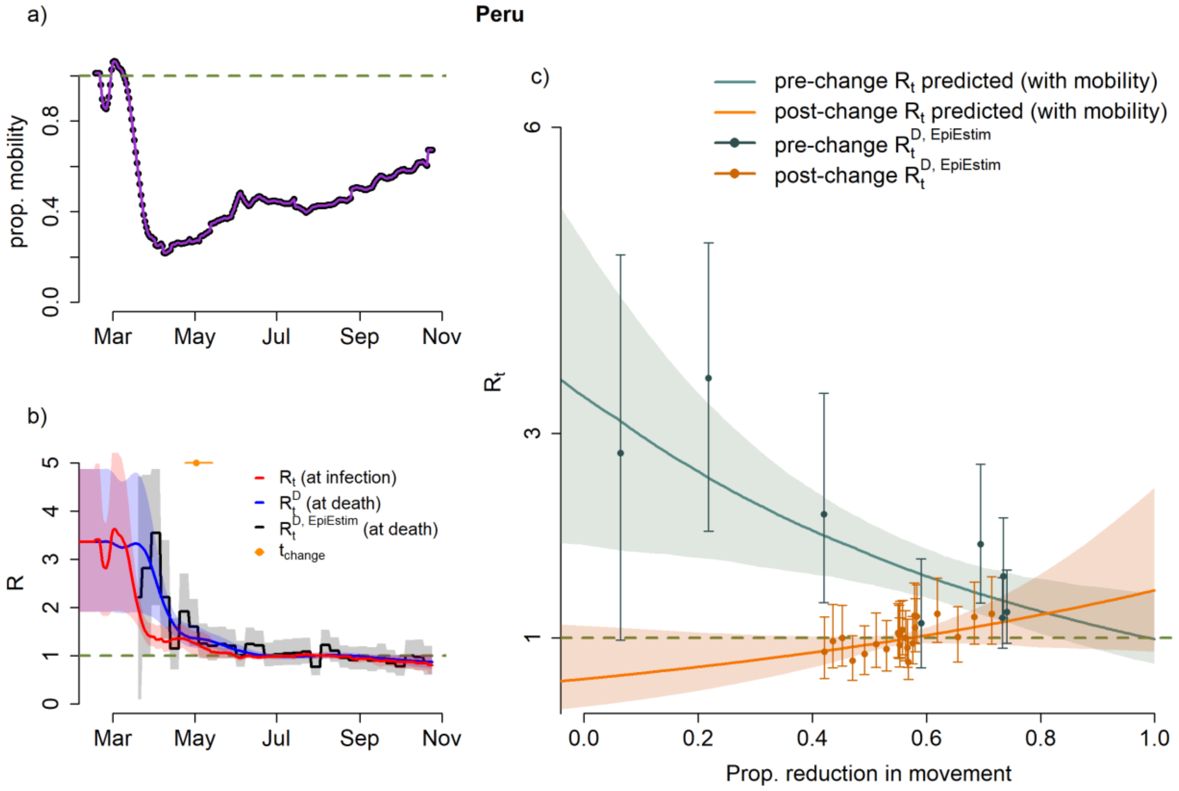
Peru
a)
prop. mobility
0.0
0.4
0.8
Mar
May
Jul
Sep
Nov
b)
R
0
1
2
3
4
5
R_t (at infection)
R_t^D (at death)
R_t^{D, EpiEstim} (at death)
t_change
c)
pre-change R_t predicted (with mobility)
post-change R_t predicted (with mobility)
pre-change R_t^{D, EpiEstim}
post-change R_t^{D, EpiEstim}
R_t
1
3
6
0.0
0.2
0.4
0.6
0.8
1.0
Prop. reduction in movement

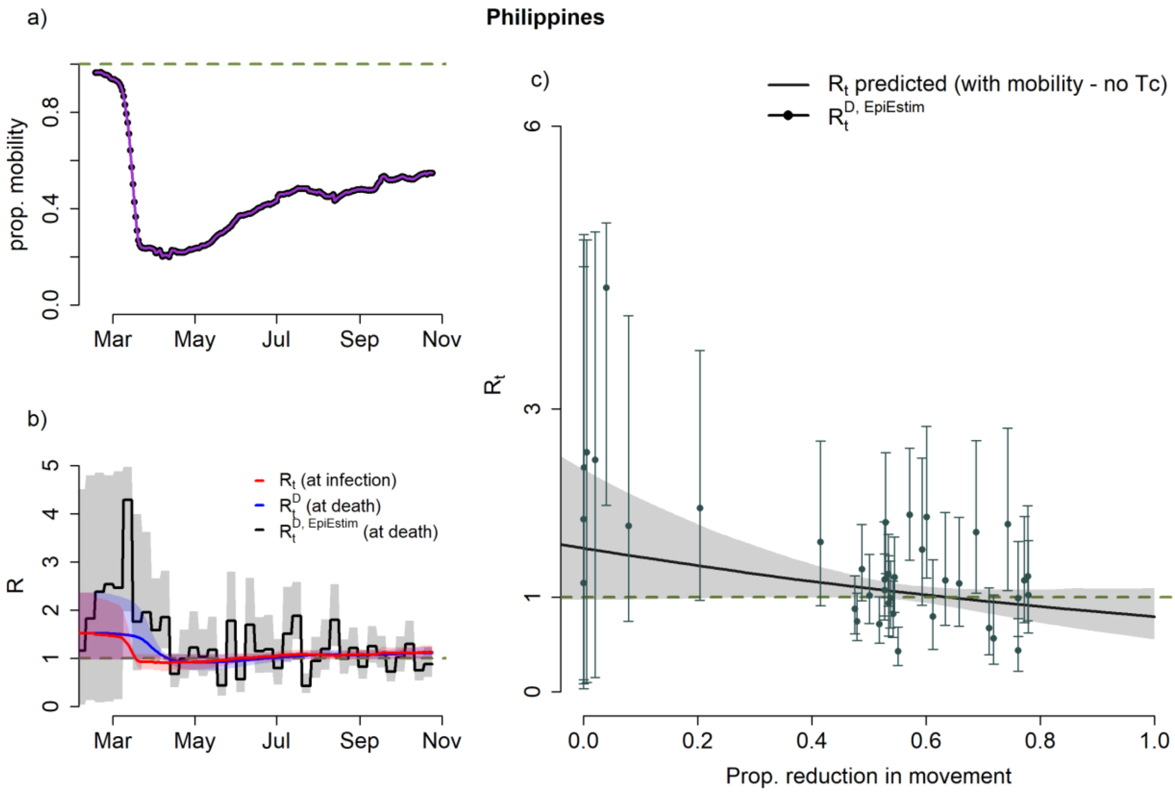
Philippines
a)
prop. mobility
0.0
0.4
0.8
Mar
May
Jul
Sep
Nov
b)
R
0
1
2
3
4
5
R_t (at infection)
R_t^D (at death)
$R_t^{D, EpiEstim}$ (at death)
c)
R_t predicted (with mobility - no Tc)
$R_t^{D, EpiEstim}$
R_t
0
1
3
6
0.0
0.2
0.4
0.6
0.8
1.0
Prop. reduction in movement

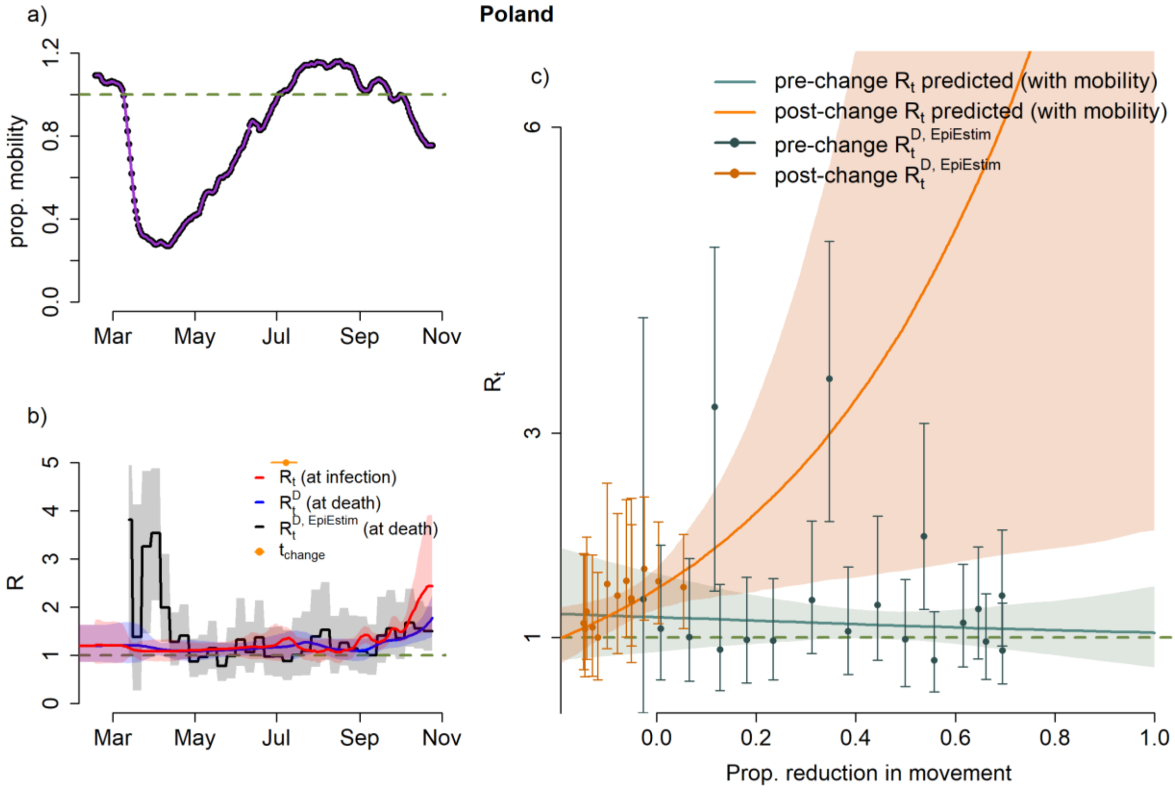
Poland
a)
prop. mobility
0.0
0.4
0.8
1.2
Mar
May
Jul
Sep
Nov
b)
R
0
1
2
3
4
5
R_t (at infection)
R_t^D (at death)
$R_t^{D, EpiEstim}$ (at death)
t_{change}
c)
pre-change R_t predicted (with mobility)
post-change R_t predicted (with mobility)
pre-change $R_t^{D, EpiEstim}$
post-change $R_t^{D, EpiEstim}$
R_t
1
3
6
0.0
0.2
0.4
0.6
0.8
1.0
Prop. reduction in movement

Portugal

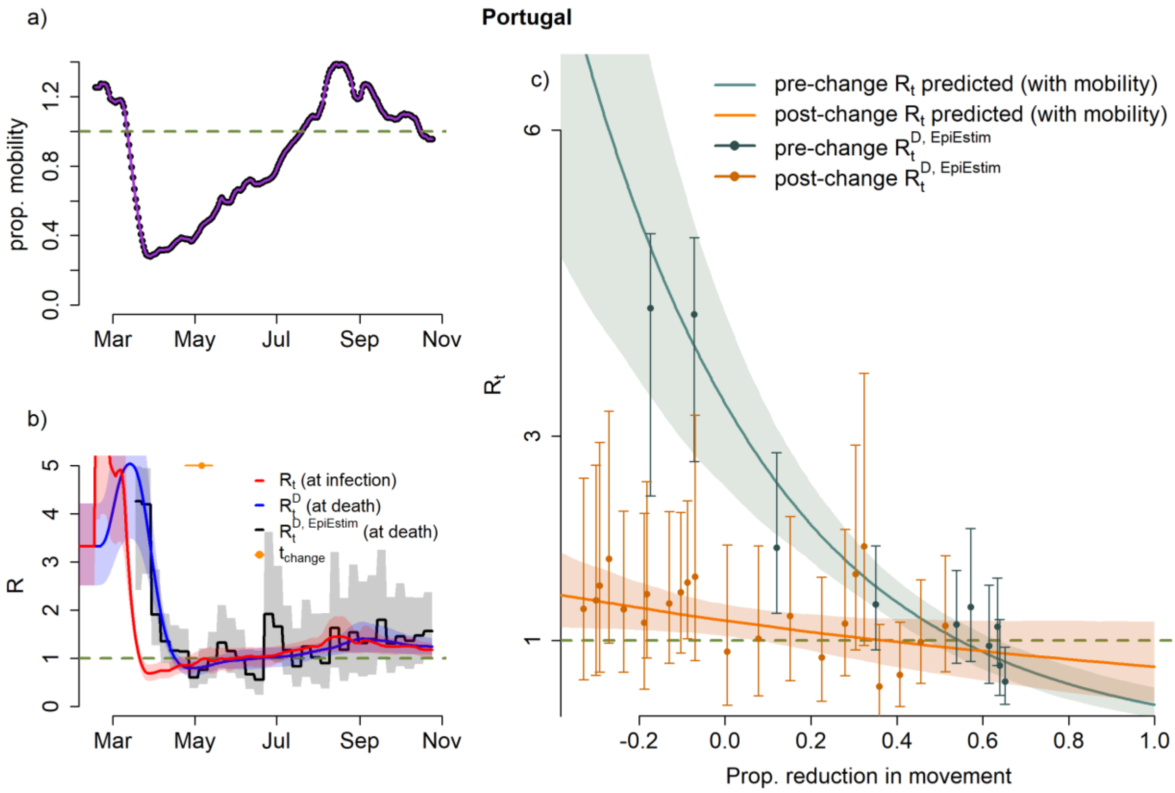

Romania

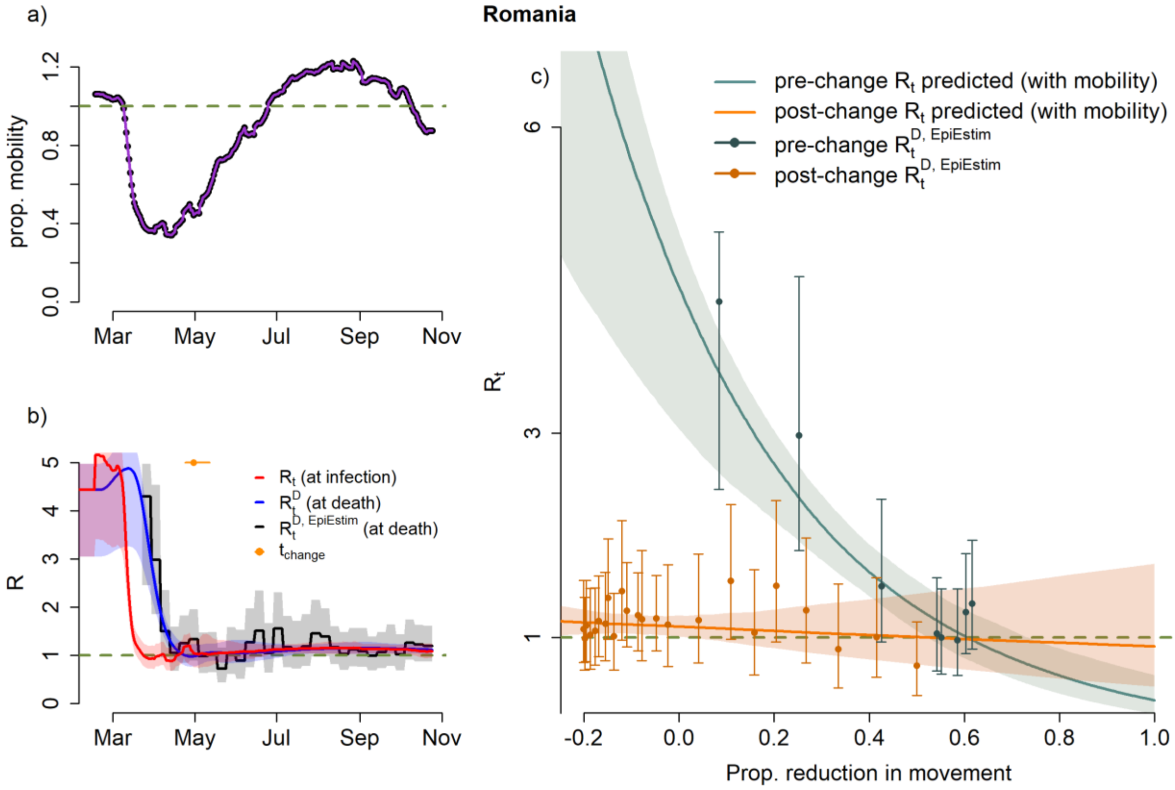

Russia

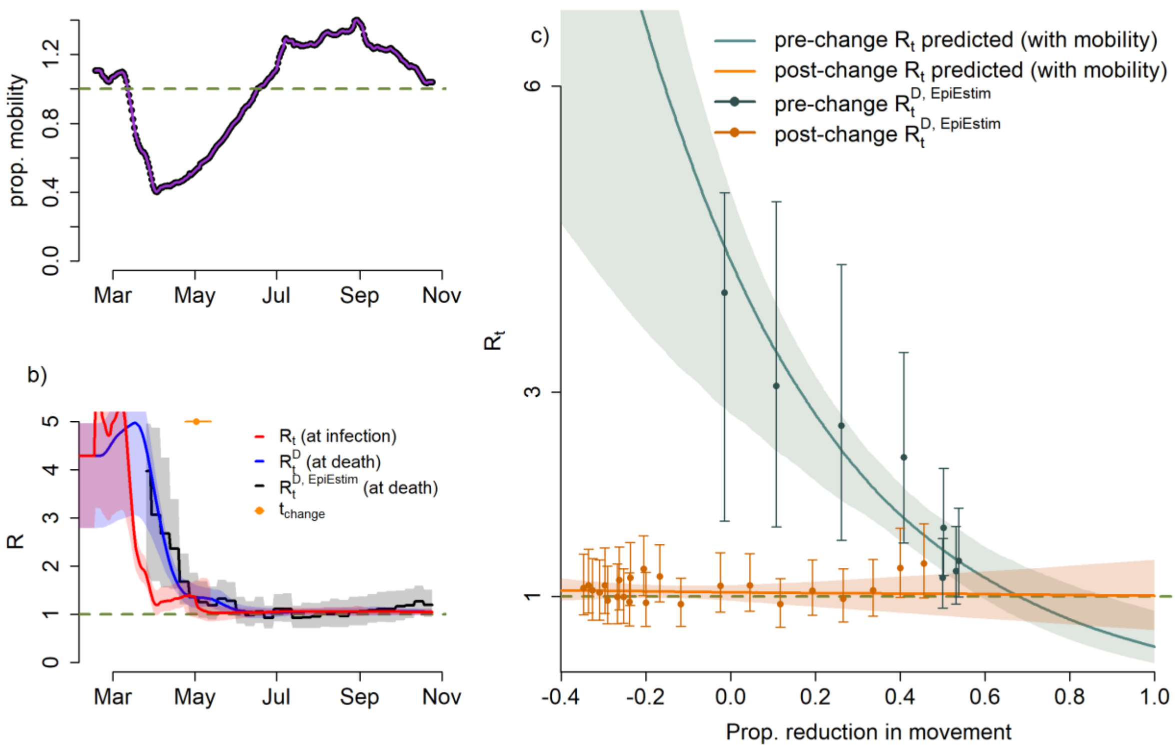

Saudi Arabia

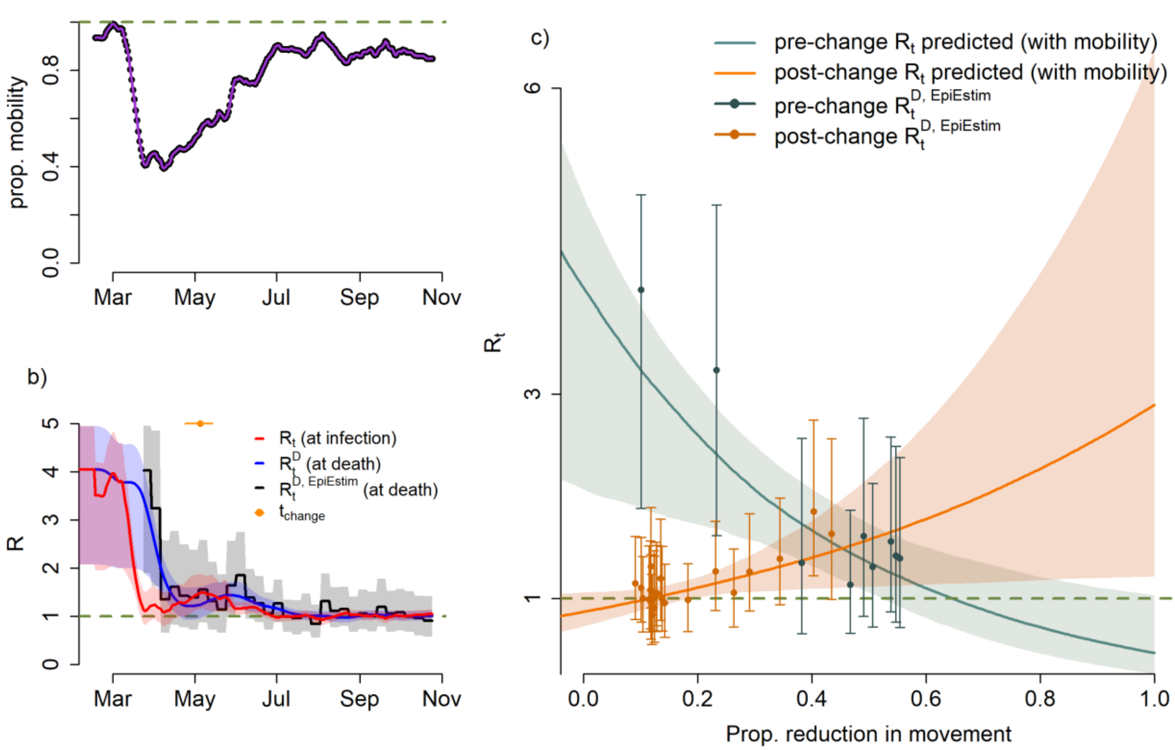

South Africa

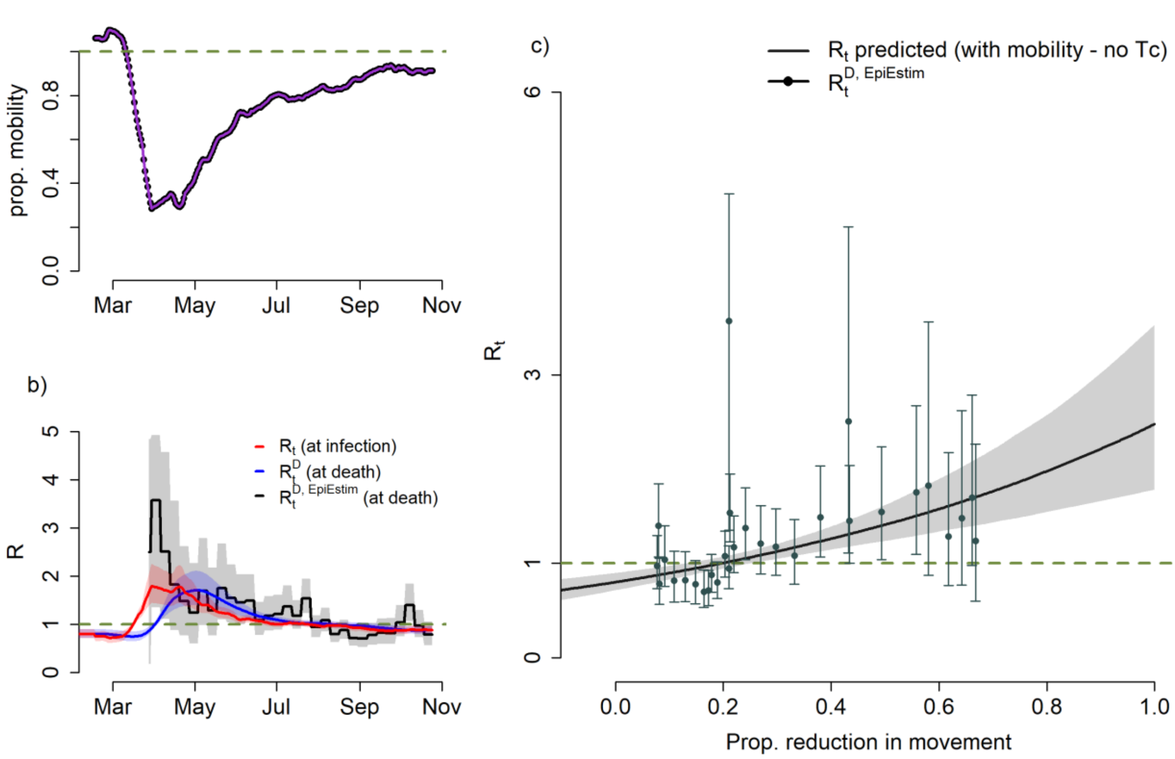

Spain

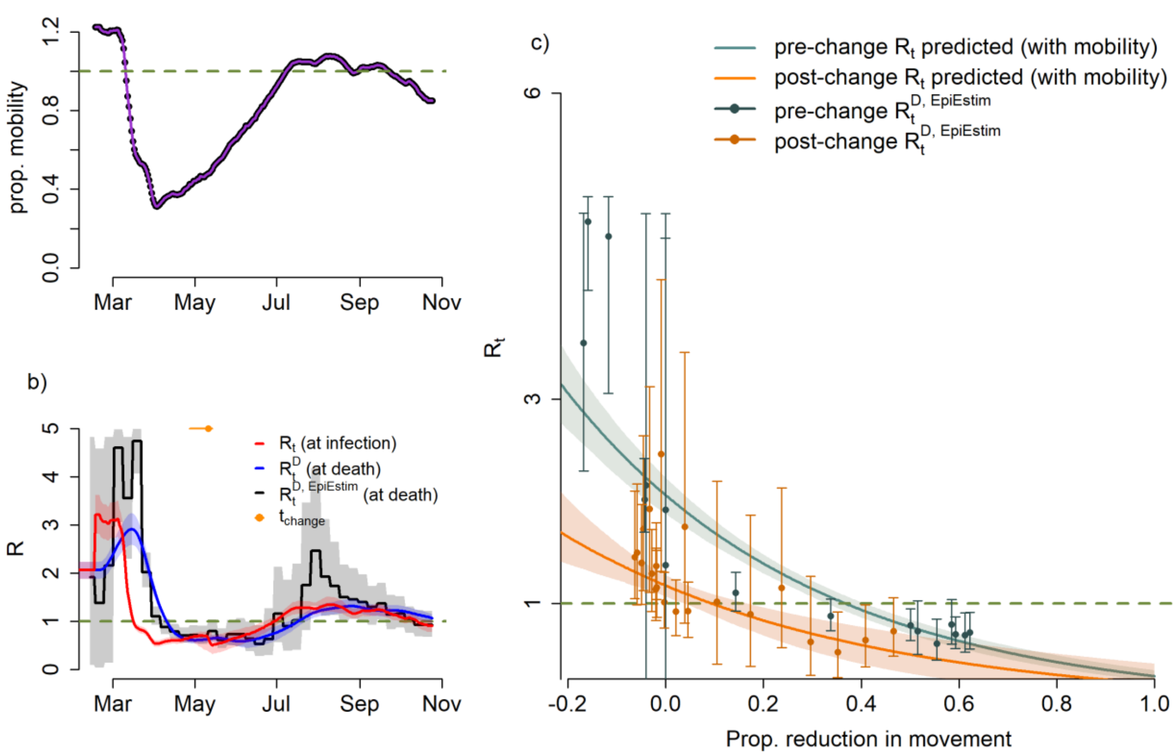

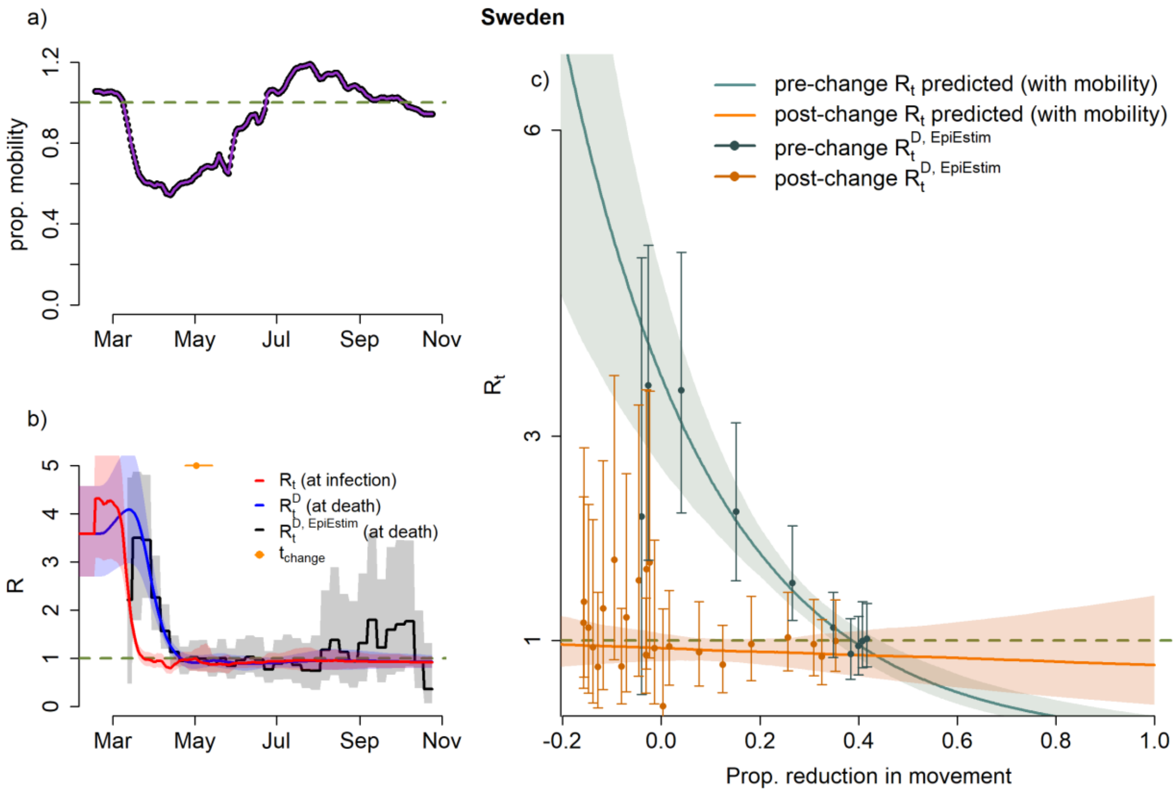
Sweden
a)
prop. mobility
0.0
0.4
0.8
1.2
Mar
May
Jul
Sep
Nov
b)
R
R_t (at infection)
R_t^D (at death)
$R_t^{D, EpiEstim}$ (at death)
t_{change}
c)
pre-change R_t predicted (with mobility)
post-change R_t predicted (with mobility)
pre-change $R_t^{D, EpiEstim}$
post-change $R_t^{D, EpiEstim}$
R_t
Prop. reduction in movement
-0.2
0.0
0.2
0.4
0.6
0.8
1.0

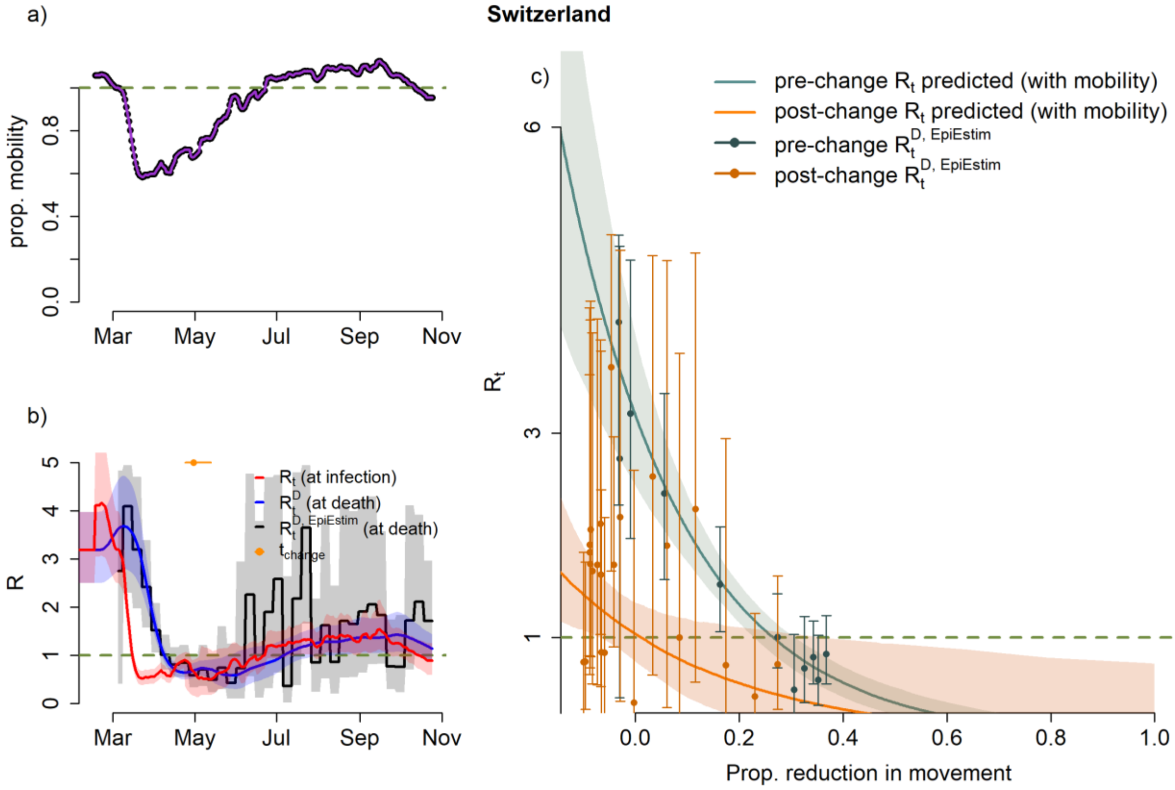
Switzerland
a)
prop. mobility
0.0
0.4
0.8
Mar
May
Jul
Sep
Nov
b)
R
R_t (at infection)
R_t^D (at death)
$R_t^{D, EpiEstim}$ (at death)
t_{change}
c)
pre-change R_t predicted (with mobility)
post-change R_t predicted (with mobility)
pre-change $R_t^{D, EpiEstim}$
post-change $R_t^{D, EpiEstim}$
R_t
Prop. reduction in movement
0.0
0.2
0.4
0.6
0.8
1.0

Turkey

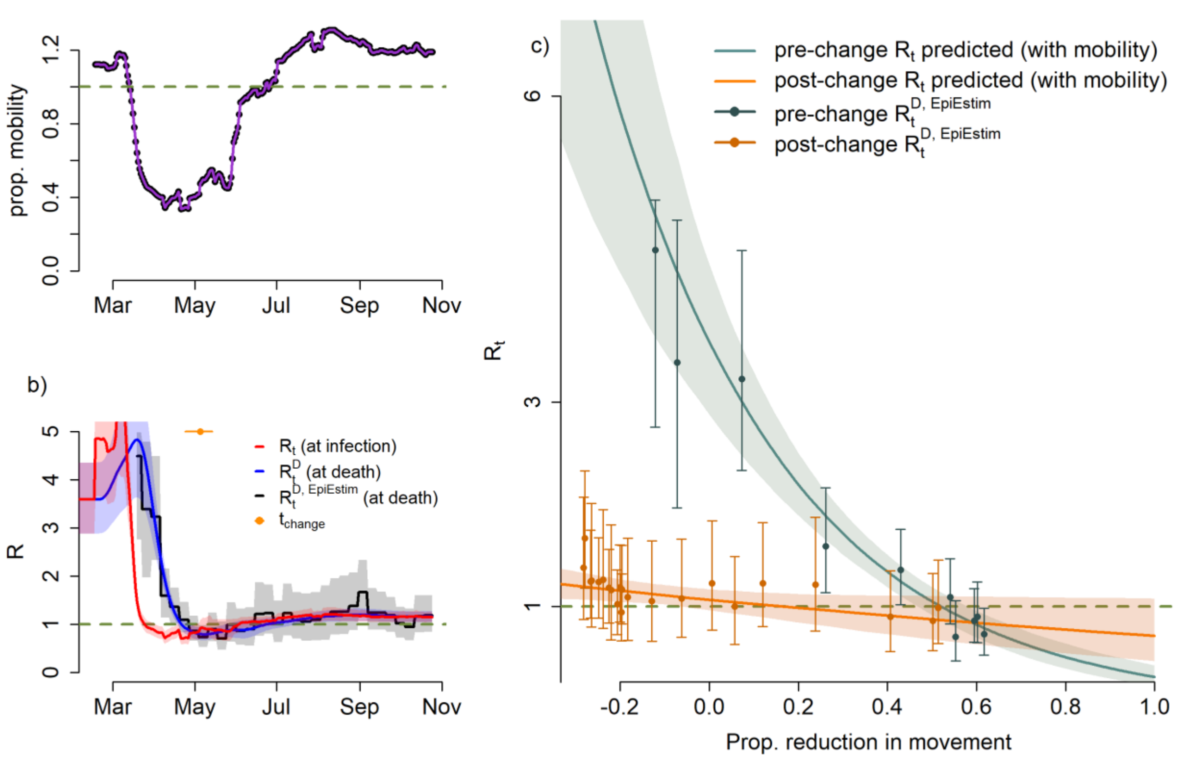

Ukraine

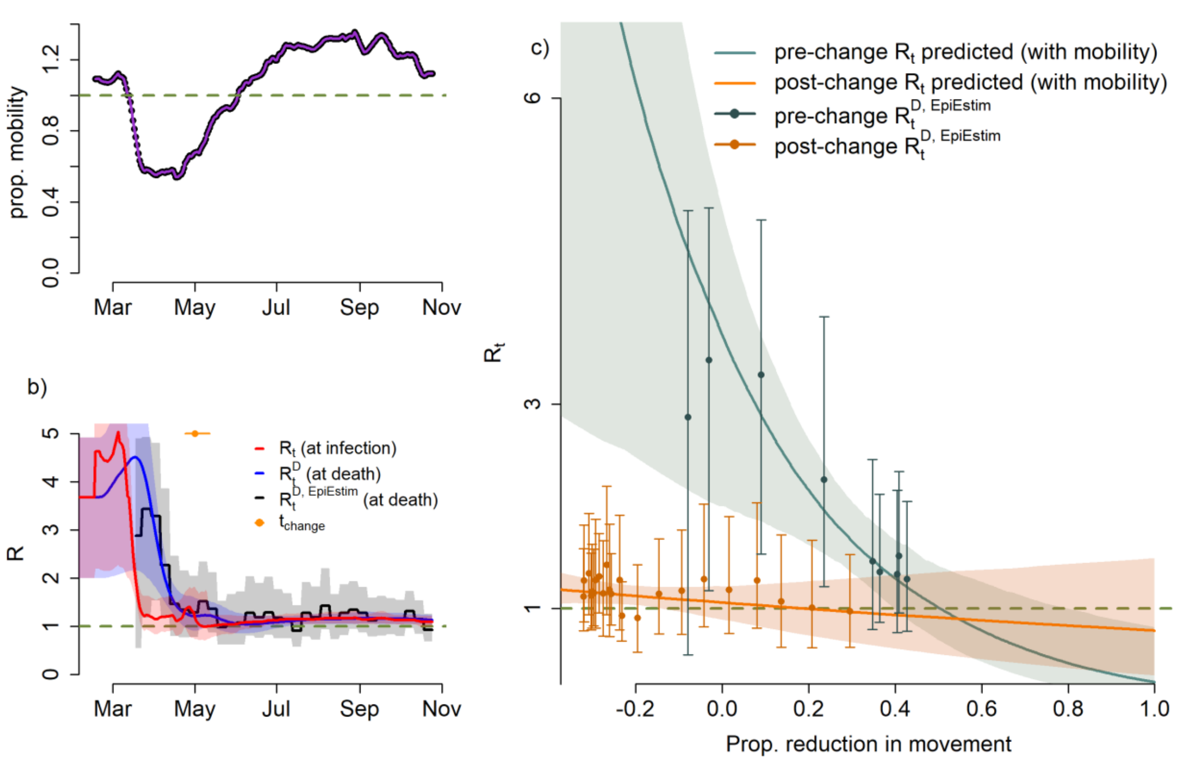

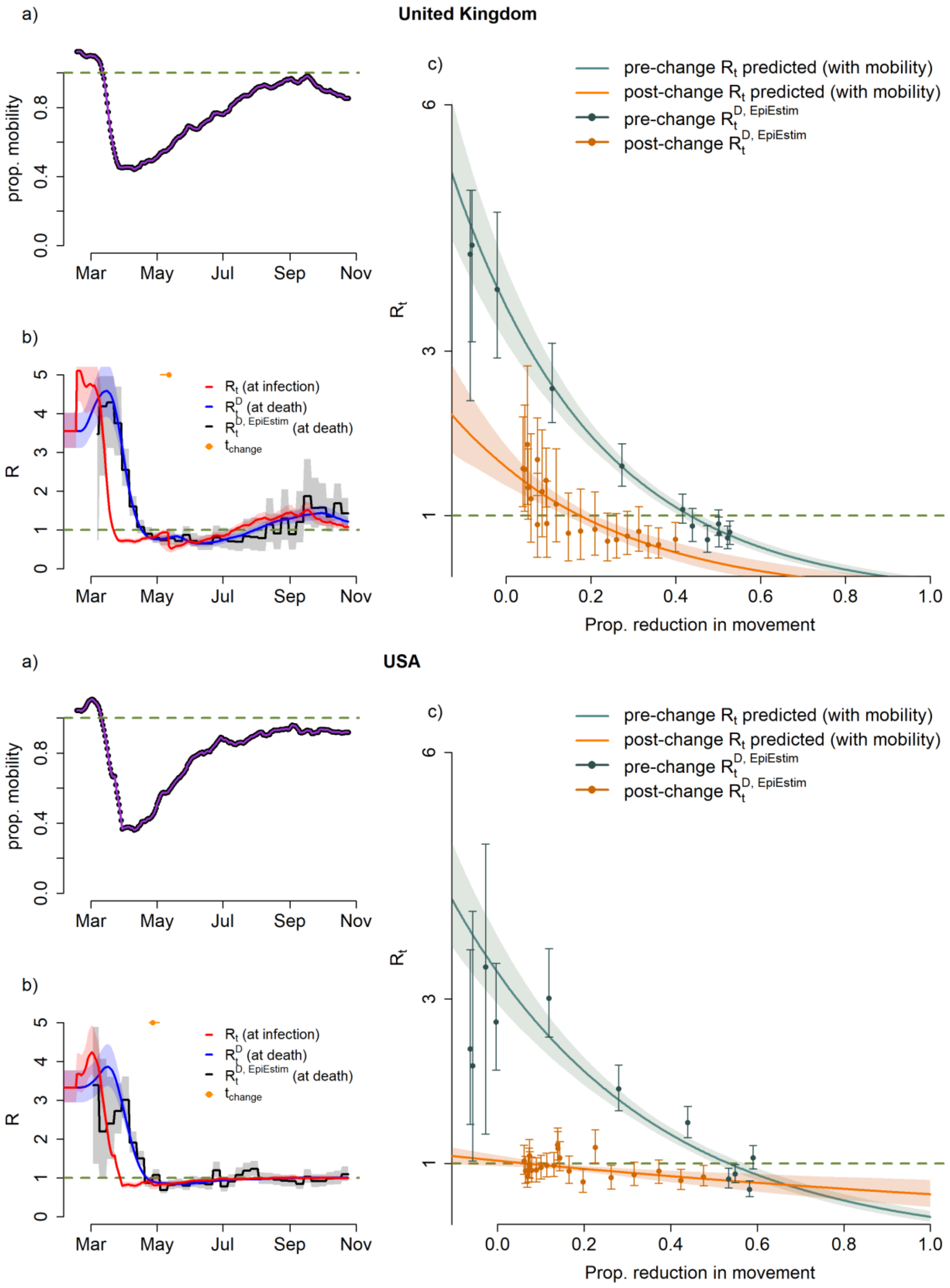

Supplementary Figure 4: Equivalent to Figure 1 in main text. Relationship between mobility and transmission. **a)** Apple-Google mobility (purple line) and daily mobility (reflecting other data streams). **b)** Estimated daily reproduction number for new infections (red) and deaths by date of reported death (blue) estimated using the best-fitting model and mobility data. Instantaneous

reproduction number estimated from deaths data alone using a daily 7-day sliding window (grey). In each case shading represents the 95% credible interval. Horizontal orange dot and line show the median and 95% CrI for the timing of the change in the relationship between mobility and transmission. **c)** Estimates of the reproduction number against changes in mobility using our best model (5 estimated parameters): Turquoise/orange lines showing the median predictions pre-post change in relationship, with shading indicating the 95% credible interval. The '*EpiEstim*' delayed effective reproduction numbers using *'EpiEstim'*-like method are shown as error-bars in turquoise/orange for approximate pre-post change in relationship with 95% credible interval.

Supplementary note 3: Improved fit using individual data stream in selected countries

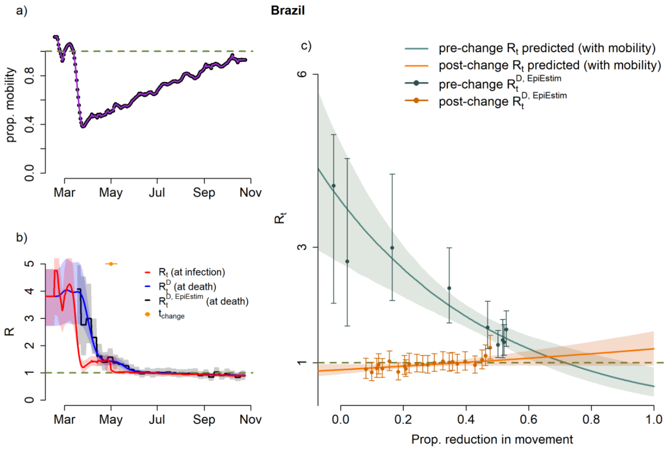

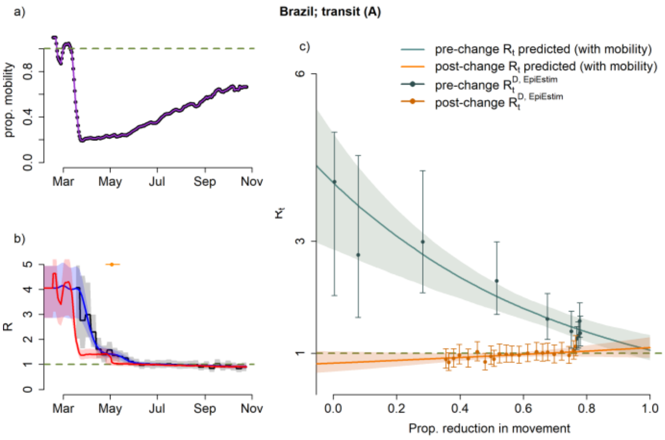

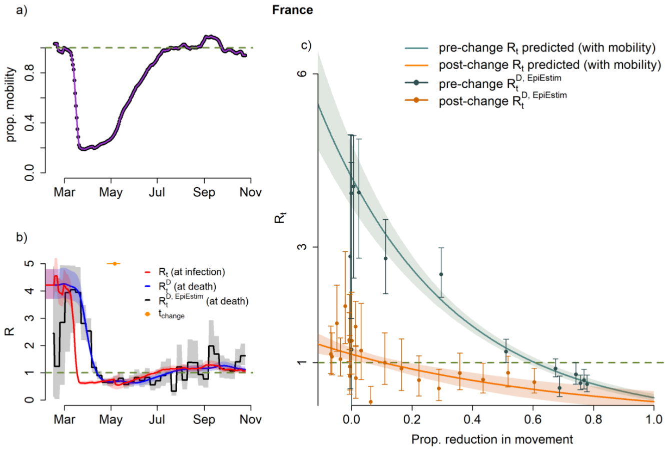

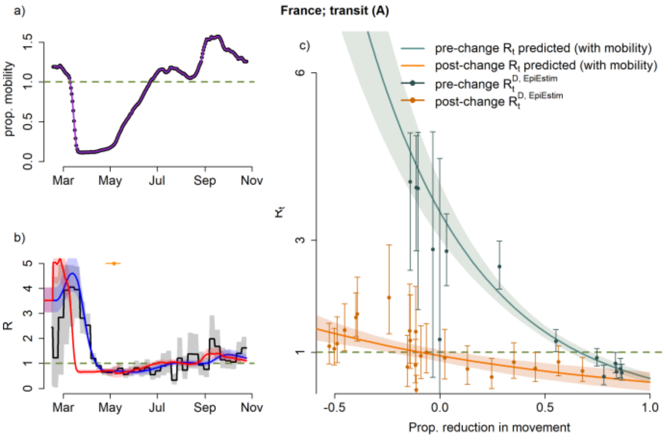

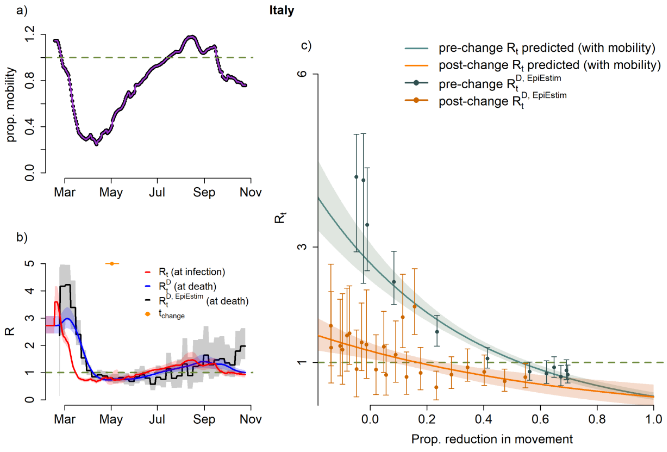

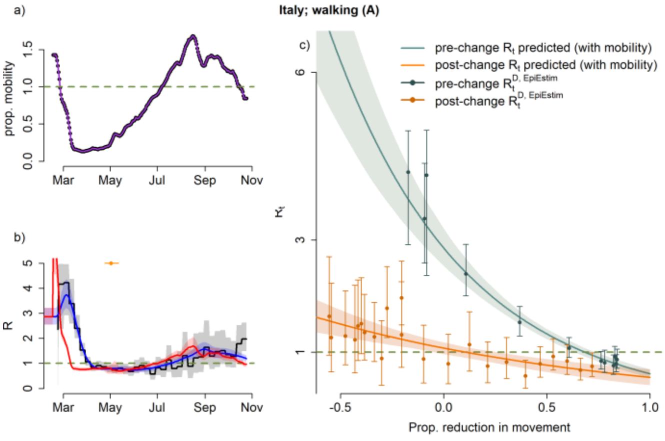

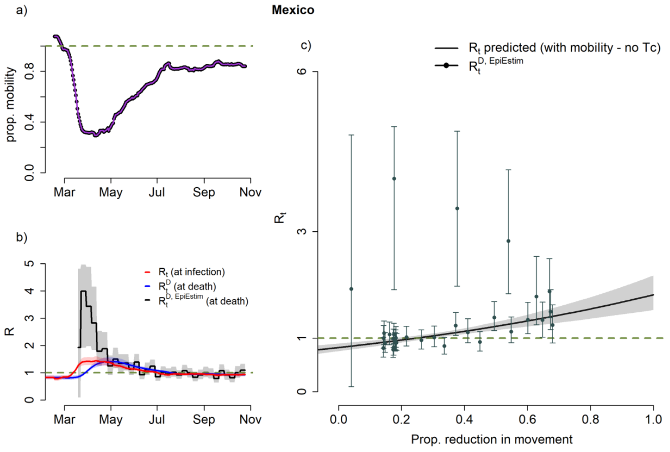
Mexico
a)
b)
c)
prop. mobility
R
R_t
Prop. reduction in movement
R_t predicted (with mobility - no Tc)
$R_t^{D, EpiEstim}$
R_t (at infection)
R_t^D (at death)
$R_t^{D, EpiEstim}$ (at death)

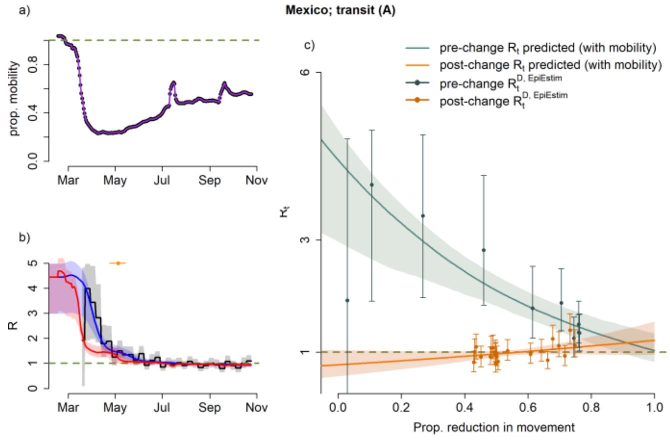
Mexico; transit (A)
a)
b)
c)
prop. mobility
R
R_t
Prop. reduction in movement
pre-change R_t predicted (with mobility)
post-change R_t predicted (with mobility)
pre-change $R_t^{D, EpiEstim}$
post-change $R_t^{D, EpiEstim}$

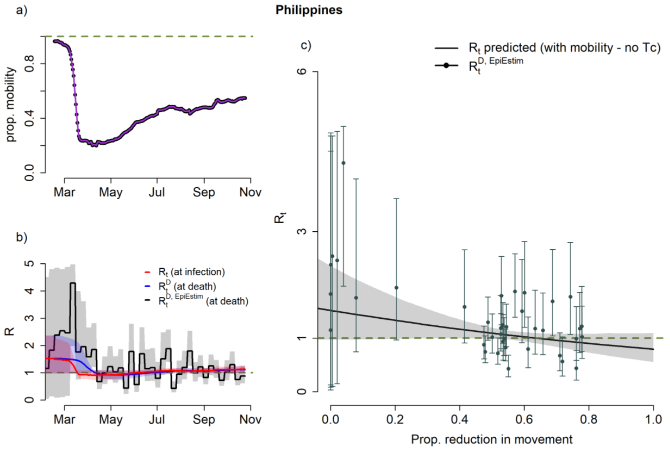
Philippines
a)
b)
c)
prop. mobility
R
R_t
Prop. reduction in movement
R_t predicted (with mobility - no Tc)
$R_t^{D, EpiEstim}$
R_t (at infection)
R_t^D (at death)
$R_t^{D, EpiEstim}$ (at death)

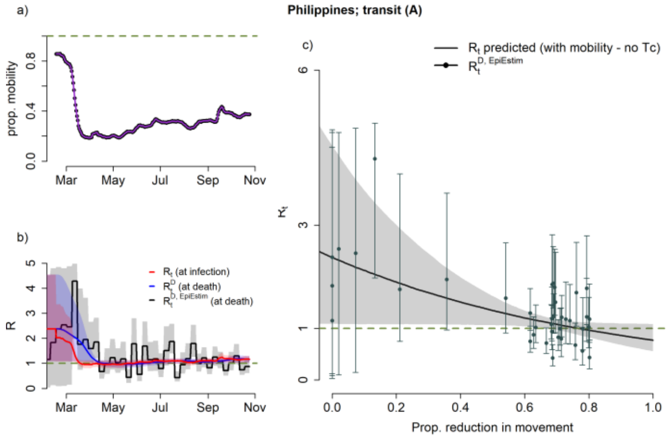
Philippines; transit (A)
a)
b)
c)
prop. mobility
R
R_t
Prop. reduction in movement
R_t predicted (with mobility - no Tc)
$R_t^{D, EpiEstim}$
R_t (at infection)
R_t^D (at death)
$R_t^{D, EpiEstim}$ (at death)

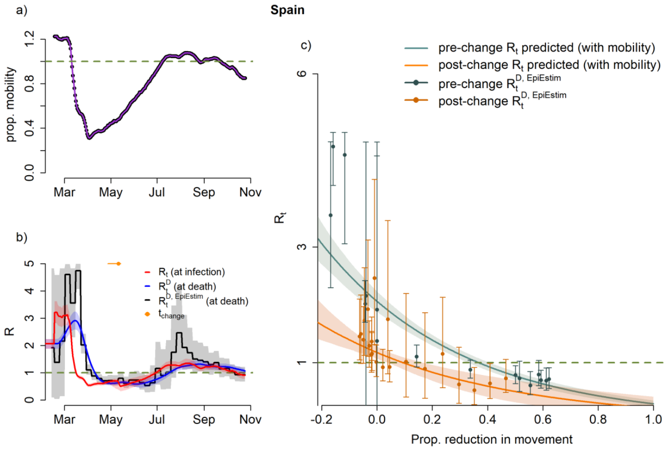
Spain
a)
b)
c)
prop. mobility
R
R_t
Prop. reduction in movement
pre-change R_t predicted (with mobility)
post-change R_t predicted (with mobility)
pre-change $R_t^{D, EpiEstim}$
post-change $R_t^{D, EpiEstim}$
R_t (at infection)
R_t^D (at death)
$R_t^{D, EpiEstim}$ (at death)
t_{change}

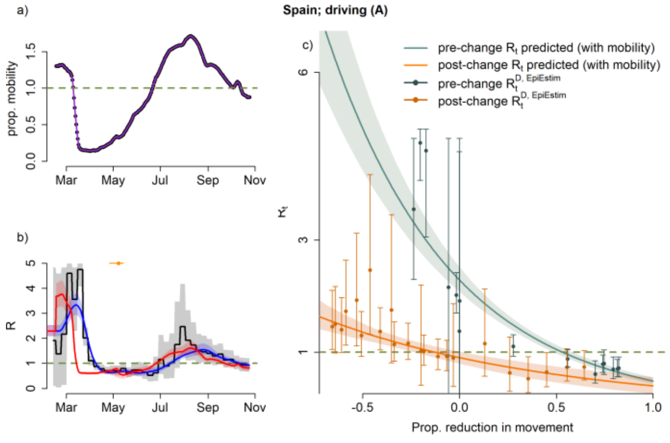
Spain; driving (A)
a)
b)
c)
prop. mobility
R
R_t
Prop. reduction in movement
pre-change R_t predicted (with mobility)
post-change R_t predicted (with mobility)
pre-change $R_t^{D, EpiEstim}$
post-change $R_t^{D, EpiEstim}$

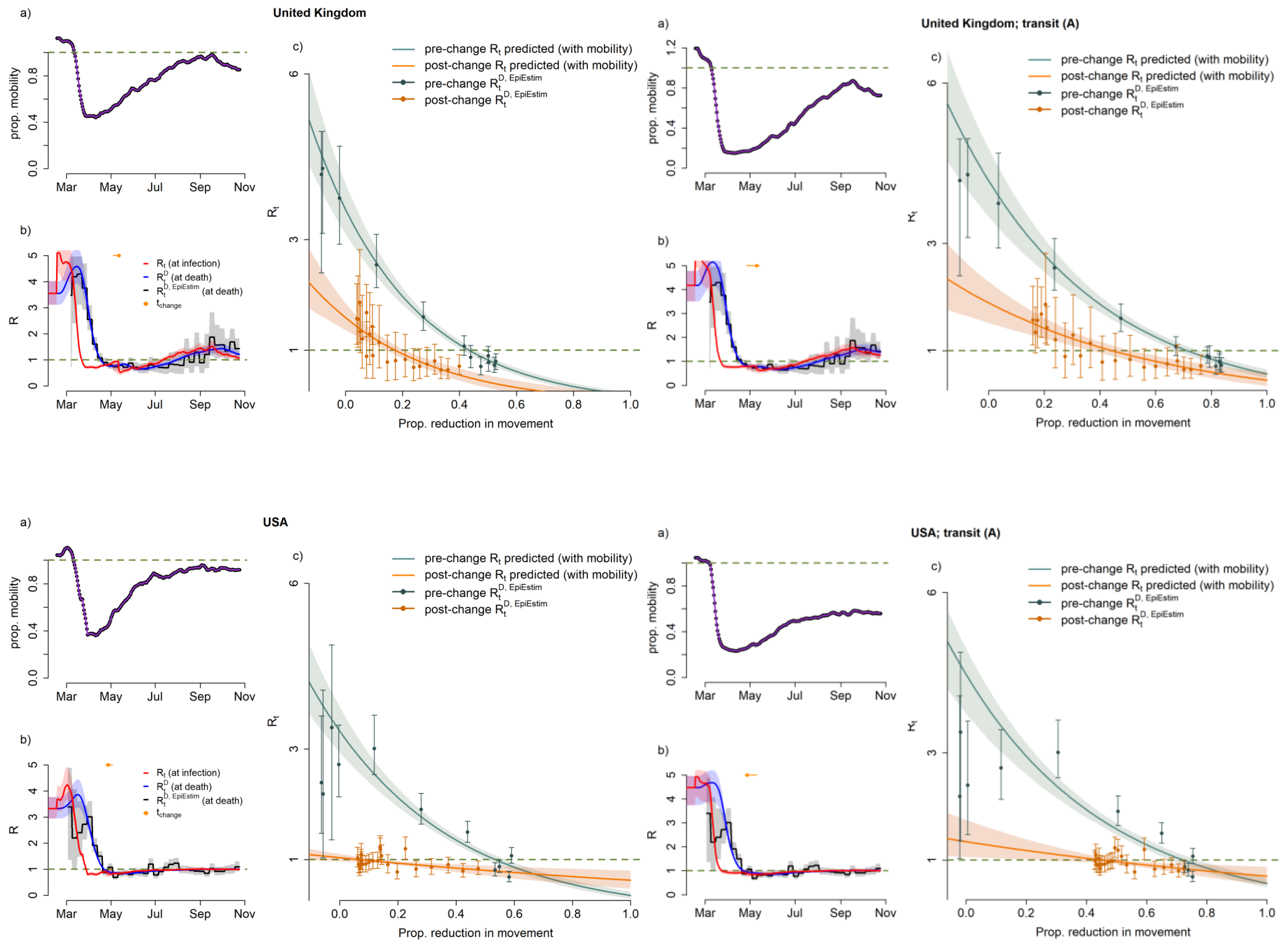

Supplementary Figure 5: For selected countries, equivalent of Supplementary Figure 4 with the combined Apple-Google mobility on the left and best mobility stream on the right. In b) and c), dots and solid lines represent median estimates, while error bars and bands represent 95%CrI.

Supplementary note 4: Mobility thresholds by countries

Supplementary Table 1: Estimated basic reproduction number and mobility thresholds with default serial interval distribution and negative binomial likelihood for Apple-Google mobility.

	First period				Second period			
**country**	R_0	β	Mobility threshold	Adjusted R-squared	R_0	β	Mobility threshold	Adjusted R-squared
**Afghanistan**	0.91 ; 95%CrI [0.76 ; 1.06]	-0.72 ; 95%CrI [-1.53 ; 0.09]	NA	0				
**Argentina**	3.26 ; 95%CrI [1.73 ; 4.81]	1.5 ; 95%CrI [0.47 ; 2.24]	0.8 ; 95%CrI [0.62 ; NA]	0.51	0.69 ; 95%CrI [0.37 ; 1.2]	-1.1 ; 95%CrI [-2.59 ; 0.1]	NA	0.27
**Australia**	3.36 ; 95%CrI [2.13 ; 4.68]	2.35 ; 95%CrI [1.13 ; 3.45]	0.51 ; 95%CrI [0.38 ; 0.76]	0.18	1.72 ; 95%CrI [0.16 ; 4.76]	2.9 ; 95%CrI [-7.36 ; 7.29]	NA	0.28
**Austria**	3.15 ; 95%CrI [2.27 ; 4.24]	3.33 ; 95%CrI [2.35 ; 4.37]	0.35 ; 95%CrI [0.28 ; 0.42]	0.83	1.24 ; 95%CrI [1.01 ; 1.48]	1.25 ; 95%CrI [-0.19 ; 3.1]	NA	0.09
**Bangladesh**	3.2 ; 95%CrI [1.65 ; 4.83]	1.56 ; 95%CrI [0.25 ; 2.6]	0.75 ; 95%CrI [0.52 ; NA]	0.26	0.93 ; 95%CrI [0.78 ; 1.12]	-0.54 ; 95%CrI [-1.18 ; 0.22]	NA	0.48
**Belgium**	3.37 ; 95%CrI [2.87 ; 3.87]	2.77 ; 95%CrI [2.34 ; 3.16]	0.44 ; 95%CrI [0.41 ; 0.48]	0.98	1.2 ; 95%CrI [1.05 ; 1.36]	2.06 ; 95%CrI [1.18 ; 3.27]	0.09 ; 95%CrI [0.02 ; 0.19]	0.49
**Bolivia**	2.68 ; 95%CrI [0.54 ; 4.82]	0.72 ; 95%CrI [-1.48 ; 1.7]	NA	0.38	0.69 ; 95%CrI [0.47 ; 1]	-0.89 ; 95%CrI [-1.64 ; -0.18]	NA	0.33
**Brazil**	3.81 ; 95%CrI [2.73 ; 4.81]	1.86 ; 95%CrI [1.13 ; 2.42]	0.72 ; 95%CrI [0.63 ; 0.93]	0.87	0.88 ; 95%CrI [0.8 ; 0.98]	-0.35 ; 95%CrI [-0.64 ; 0.02]	NA	0.73
**Canada**	3.79 ; 95%CrI [2.62 ; 4.85]	2.01 ; 95%CrI [1.24 ; 2.65]	0.66 ; 95%CrI [0.57 ; 0.82]	0.54	1.11 ; 95%CrI [0.95 ; 1.29]	1.19 ; 95%CrI [0.52 ; 2.02]	0.09 ; 95%CrI [-0.09 ; 0.17]	0.2
**Chile**	4.19 ; 95%CrI [2.53 ; 4.98]	2.02 ; 95%CrI [0.99 ; 2.56]	0.71 ; 95%CrI [0.61 ; 0.98]	0.68	1.39 ; 95%CrI [0.98 ; 1.96]	1.46 ; 95%CrI [0.29 ; 2.63]	0.23 ; 95%CrI [-0.05 ; 0.28]	0.31

**Colombia**	4.09 ; 95%CrI [2.2 ; 4.96]	1.97 ; 95%CrI [0.88 ; 2.57]	0.71 ; 95%CrI [0.58 ; NA]	0.76	0.82 ; 95%CrI [0.67 ; 0.99]	-0.92 ; 95%CrI [-1.53 ; -0.31]	NA	0.57
**Costa_Rica**	2.56 ; 95%CrI [0.9 ; 4.73]	0.63 ; 95%CrI [-1.42 ; 2.31]	NA	-0.01	1.04 ; 95%CrI [0.5 ; 2.23]	-0.26 ; 95%CrI [-2.27 ; 1.93]	NA	0.38
**Czechia**	4.17 ; 95%CrI [2.65 ; 4.96]	3.47 ; 95%CrI [2.19 ; 4.43]	0.41 ; 95%CrI [0.33 ; 0.51]	0.91	3.24 ; 95%CrI [0.95 ; 4.9]	2.07 ; 95%CrI [-1.32 ; 3.23]	NA	0.07
**Denmark**	4 ; 95%CrI [2.7 ; 4.92]	3.85 ; 95%CrI [2.52 ; 4.85]	0.36 ; 95%CrI [0.3 ; 0.45]	0.96	0.99 ; 95%CrI [0.73 ; 1.26]	1.69 ; 95%CrI [0.17 ; 3.32]	-0.01 ; 95%CrI [-0.19 ; 0.57]	0.27
**Dominican_R.**	1.08 ; 95%CrI [0.88 ; 1.32]	0.02 ; 95%CrI [-0.97 ; 1.07]	NA	0.06				
**Ecuador**	0.87 ; 95%CrI [0.76 ; 1]	-0.35 ; 95%CrI [-1.06 ; 0.43]	NA	0				
**Egypt**	3.37 ; 95%CrI [2.23 ; 4.7]	1.86 ; 95%CrI [1.03 ; 2.57]	0.65 ; 95%CrI [0.57 ; 0.82]	0.85	0.99 ; 95%CrI [0.86 ; 1.13]	0.62 ; 95%CrI [-0.28 ; 1.5]	NA	0.29
**France**	4.21 ; 95%CrI [3.7 ; 4.8]	2.36 ; 95%CrI [2.13 ; 2.6]	0.61 ; 95%CrI [0.58 ; 0.64]	0.7	1.15 ; 95%CrI [1.04 ; 1.26]	1.25 ; 95%CrI [0.77 ; 1.88]	0.11 ; 95%CrI [0.04 ; 0.19]	0.32
**Germany**	3.82 ; 95%CrI [3.12 ; 4.58]	3.48 ; 95%CrI [2.8 ; 4.08]	0.39 ; 95%CrI [0.35 ; 0.43]	0.94	0.97 ; 95%CrI [0.87 ; 1.09]	1.51 ; 95%CrI [0.96 ; 2.08]	-0.02 ; 95%CrI [-0.1 ; 0.06]	0.45
**Guatemala**	0.77 ; 95%CrI [0.63 ; 0.95]	-1.42 ; 95%CrI [-2.08 ; -0.72]	NA	0.42				
**Honduras**	0.98 ; 95%CrI [0.82 ; 1.12]	-0.52 ; 95%CrI [-1.13 ; 0.1]	NA	0.06				
**Hungary**	3.51 ; 95%CrI [2.03 ; 4.79]	2.1 ; 95%CrI [0.99 ; 2.93]	0.6 ; 95%CrI [0.49 ; 0.82]	0.68	1.65 ; 95%CrI [1.37 ; 2.04]	1.73 ; 95%CrI [0.73 ; 3.01]	0.29 ; 95%CrI [0.18 ; 0.55]	0.26
**India**	3.6 ; 95%CrI [2.44 ; 4.86]	1.53 ; 95%CrI [0.84 ; 2.1]	0.85 ; 95%CrI [0.72 ; NA]	0.65	0.8 ; 95%CrI [0.71 ; 0.91]	-0.75 ; 95%CrI [-1.06 ; -0.44]	NA	0.84
**Indonesia**	2.55 ; 95%CrI [1.79 ; 3.57]	1.5 ; 95%CrI [0.76 ; 2.24]	0.64 ; 95%CrI [0.5 ; 0.86]	0.44	0.95 ; 95%CrI [0.7 ; 1.33]	-0.37 ; 95%CrI [-1.2 ; 0.55]	NA	0.06

**Iraq**	1.76 ; 95%CrI [1.17 ; 2.67]	1.49 ; 95%CrI [-0.05 ; 3.39]	NA	0.28	0.78 ; 95%CrI [0.68 ; 0.9]	-3.61 ; 95%CrI [-4.92 ; -2.35]	NA	0.87
**Ireland**	3.91 ; 95%CrI [2.83 ; 4.88]	2.75 ; 95%CrI [1.98 ; 3.32]	0.5 ; 95%CrI [0.43 ; 0.58]	0.46	1.65 ; 95%CrI [1.08 ; 2.51]	1.88 ; 95%CrI [0.52 ; 3.3]	0.27 ; 95%CrI [0.08 ; 0.42]	0.14
**Israel**	3.07 ; 95%CrI [1.96 ; 4.62]	2.14 ; 95%CrI [1.09 ; 3.21]	0.52 ; 95%CrI [0.42 ; 0.69]	0.6	1.46 ; 95%CrI [1.18 ; 1.82]	2.85 ; 95%CrI [1.03 ; 4.83]	0.14 ; 95%CrI [0.09 ; 0.24]	0.49
**Italy**	2.73 ; 95%CrI [2.43 ; 3.07]	1.89 ; 95%CrI [1.64 ; 2.13]	0.53 ; 95%CrI [0.5 ; 0.57]	0.92	1.2 ; 95%CrI [1.06 ; 1.34]	1.09 ; 95%CrI [0.58 ; 1.65]	0.17 ; 95%CrI [0.08 ; 0.26]	0.27
**Japan**	1.52 ; 95%CrI [1.14 ; 2.01]	1.14 ; 95%CrI [0.31 ; 1.92]	0.37 ; 95%CrI [0.24 ; 0.63]	0.18	1 ; 95%CrI [0.77 ; 1.26]	-3.32 ; 95%CrI [-6.75 ; 0.36]	NA	0.48
**Libya**	1.07 ; 95%CrI [0.81 ; 1.38]	-1.22 ; 95%CrI [-2.86 ; 0.38]	NA	0.17				
**Mexico**	0.82 ; 95%CrI [0.76 ; 0.9]	-0.79 ; 95%CrI [-1.04 ; -0.55]	NA	0.05				
**Moldova**	2.78 ; 95%CrI [1.44 ; 4.59]	3.51 ; 95%CrI [0.23 ; 7.93]	0.29 ; 95%CrI [0.17 ; NA]	0.49	1.12 ; 95%CrI [0.72 ; 1.73]	-0.06 ; 95%CrI [-3.12 ; 3.26]	NA	0
**Morocco**	3.12 ; 95%CrI [2.2 ; 4.26]	2.34 ; 95%CrI [1.47 ; 3.23]	0.48 ; 95%CrI [0.39 ; 0.62]	0.48	2.59 ; 95%CrI [1.08 ; 4.61]	3.79 ; 95%CrI [-0.28 ; 6.65]	NA	0.65
**Netherlands**	3.39 ; 95%CrI [2.78 ; 4.06]	2.47 ; 95%CrI [1.99 ; 2.91]	0.5 ; 95%CrI [0.45 ; 0.55]	0.93	1.55 ; 95%CrI [1.26 ; 1.87]	1.83 ; 95%CrI [1.08 ; 2.82]	0.24 ; 95%CrI [0.16 ; 0.34]	0.29
**Pakistan**	3.43 ; 95%CrI [1.78 ; 4.88]	1.86 ; 95%CrI [0.45 ; 2.8]	0.66 ; 95%CrI [0.54 ; NA]	0.83	0.74 ; 95%CrI [0.58 ; 0.97]	-1.7 ; 95%CrI [-3.15 ; -0.18]	NA	0.12
**Panama**	2.53 ; 95%CrI [1.54 ; 3.93]	1.81 ; 95%CrI [0.53 ; 2.97]	0.52 ; 95%CrI [0.42 ; 0.9]	0.39	2.72 ; 95%CrI [0.6 ; 4.89]	3.54 ; 95%CrI [-1.9 ; 5.85]	NA	0.31
**Paraguay**	1.81 ; 95%CrI [0.9 ; 3.76]	2.07 ; 95%CrI [-1.74 ; 6.53]	NA	0				
**Peru**	3.36 ; 95%CrI [1.91 ; 4.87]	1.22 ; 95%CrI [0.32 ; 1.81]	0.99 ; 95%CrI [0.83 ; NA]	0.6	0.6 ; 95%CrI [0.32 ; 1.12]	-0.9 ; 95%CrI [-2.02 ; 0.24]	NA	0.37

**Philippines**	1.52 ; 95%CrI [0.97 ; 2.36]	0.64 ; 95%CrI [-0.1 ; 1.43]	NA	0.41				
**Poland**	1.2 ; 95%CrI [0.86 ; 1.63]	0.14 ; 95%CrI [-0.53 ; 0.79]	NA	0.01	1.48 ; 95%CrI [1.27 ; 1.68]	-2.01 ; 95%CrI [-3.66 ; -0.41]	NA	0.45
**Portugal**	3.32 ; 95%CrI [2.51 ; 4.22]	2.19 ; 95%CrI [1.61 ; 2.75]	0.55 ; 95%CrI [0.47 ; 0.64]	0.91	1.19 ; 95%CrI [1.04 ; 1.36]	0.49 ; 95%CrI [0 ; 1.06]	NA	0.19
**Romania**	4.43 ; 95%CrI [3.05 ; 4.97]	2.44 ; 95%CrI [1.64 ; 2.92]	0.61 ; 95%CrI [0.52 ; 0.73]	0.93	1.11 ; 95%CrI [1.01 ; 1.21]	0.19 ; 95%CrI [-0.39 ; 0.72]	NA	0
**Russia**	4.28 ; 95%CrI [2.79 ; 4.97]	2.14 ; 95%CrI [1.26 ; 2.61]	0.68 ; 95%CrI [0.59 ; 0.89]	0.89	1.04 ; 95%CrI [0.96 ; 1.11]	0.03 ; 95%CrI [-0.22 ; 0.39]	NA	0.29
**Saudi_Arabia**	4.04 ; 95%CrI [2.07 ; 4.94]	2.16 ; 95%CrI [0.76 ; 2.88]	0.65 ; 95%CrI [0.53 ; NA]	0.87	0.87 ; 95%CrI [0.73 ; 1.06]	-1.22 ; 95%CrI [-2.15 ; -0.16]	NA	0.64
**South_Africa**	0.8 ; 95%CrI [0.71 ; 0.9]	-1.13 ; 95%CrI [-1.58 ; -0.71]	NA	0.1				
**Spain**	2.06 ; 95%CrI [1.88 ; 2.23]	1.97 ; 95%CrI [1.74 ; 2.18]	0.37 ; 95%CrI [0.33 ; 0.41]	0.77	1.18 ; 95%CrI [1.07 ; 1.28]	1.7 ; 95%CrI [1.05 ; 2.64]	0.1 ; 95%CrI [0.04 ; 0.17]	0.47
**Sweden**	3.58 ; 95%CrI [2.69 ; 4.57]	3.31 ; 95%CrI [2.38 ; 4.07]	0.39 ; 95%CrI [0.35 ; 0.44]	0.82	0.92 ; 95%CrI [0.79 ; 1.07]	0.2 ; 95%CrI [-0.51 ; 0.98]	NA	0.08
**Switzerland**	3.19 ; 95%CrI [2.5 ; 3.97]	4.37 ; 95%CrI [3.29 ; 5.42]	0.27 ; 95%CrI [0.23 ; 0.31]	0.94	1.04 ; 95%CrI [0.77 ; 1.34]	3.14 ; 95%CrI [0.49 ; 6.61]	0.01 ; 95%CrI [-0.07 ; 0.36]	0.04
**Turkey**	3.59 ; 95%CrI [2.88 ; 4.36]	2.45 ; 95%CrI [1.96 ; 2.94]	0.52 ; 95%CrI [0.47 ; 0.58]	0.94	1.06 ; 95%CrI [0.96 ; 1.17]	0.4 ; 95%CrI [0.04 ; 0.78]	0.16 ; 95%CrI [-0.06 ; NA]	0.34
**Ukraine**	3.68 ; 95%CrI [2 ; 4.92]	2.57 ; 95%CrI [0.94 ; 3.73]	0.51 ; 95%CrI [0.4 ; 0.8]	0.81	1.06 ; 95%CrI [0.89 ; 1.22]	0.3 ; 95%CrI [-0.24 ; 0.96]	NA	0.02
**United_Kingdom**	3.55 ; 95%CrI [3.11 ; 4.02]	2.92 ; 95%CrI [2.6 ; 3.24]	0.43 ; 95%CrI [0.41 ; 0.46]	0.98	1.59 ; 95%CrI [1.37 ; 1.85]	2.64 ; 95%CrI [1.93 ; 3.45]	0.18 ; 95%CrI [0.14 ; 0.21]	0.69
**USA**	3.33 ; 95%CrI [2.94 ; 3.77]	2.25 ; 95%CrI [1.98 ; 2.53]	0.54 ; 95%CrI [0.51 ; 0.57]	0.72	1.03 ; 95%CrI [0.97 ; 1.1]	0.5 ; 95%CrI [0.2 ; 0.82]	0.07 ; 95%CrI [-0.14 ; 0.14]	0.13

Supplementary note 5: Correlation between R_0 and the mobility threshold

We evaluated the correlation between estimated mobility thresholds and basic reproduction number across countries to ensure the variation in the estimated thresholds was not driven by the variation in estimated basic reproduction number.

We found no evidence of a correlation between the estimated mobility threshold and the estimated basic reproduction number.

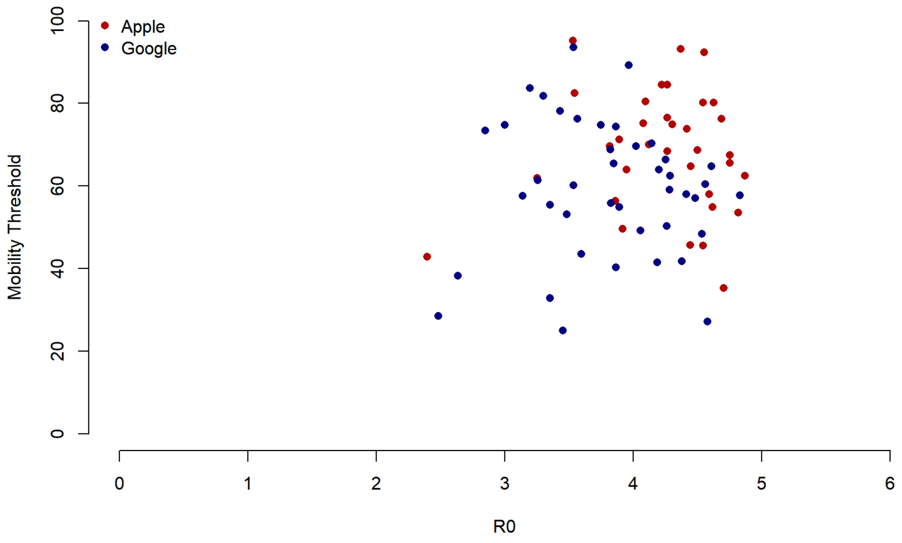

Supplementary Figure 6: Relationship between the estimated (medians) basic reproduction number and the estimated (medians) mobility thresholds. As we evaluate the relation with the basic reproduction number, this is based on data up to the 10th May 2020. For both Apple and Google mobility, we found no significant correlation between the estimated R_0 and mobility thresholds.

Supplementary note 6: Sensitivity of estimated parameters to early epidemic dynamics

As the reporting of deaths might have changed during the country-specific early phase of the epidemic, we re-estimated the mobility-transmission relationship excluding from the likelihood all days previous to the two consecutive weeks reporting each at least 10 deaths (the criteria for sustained epidemic). However, the estimated parameters were robust to excluding the very early dynamic from the likelihood (i.e. before our sustained epidemic criteria is met).

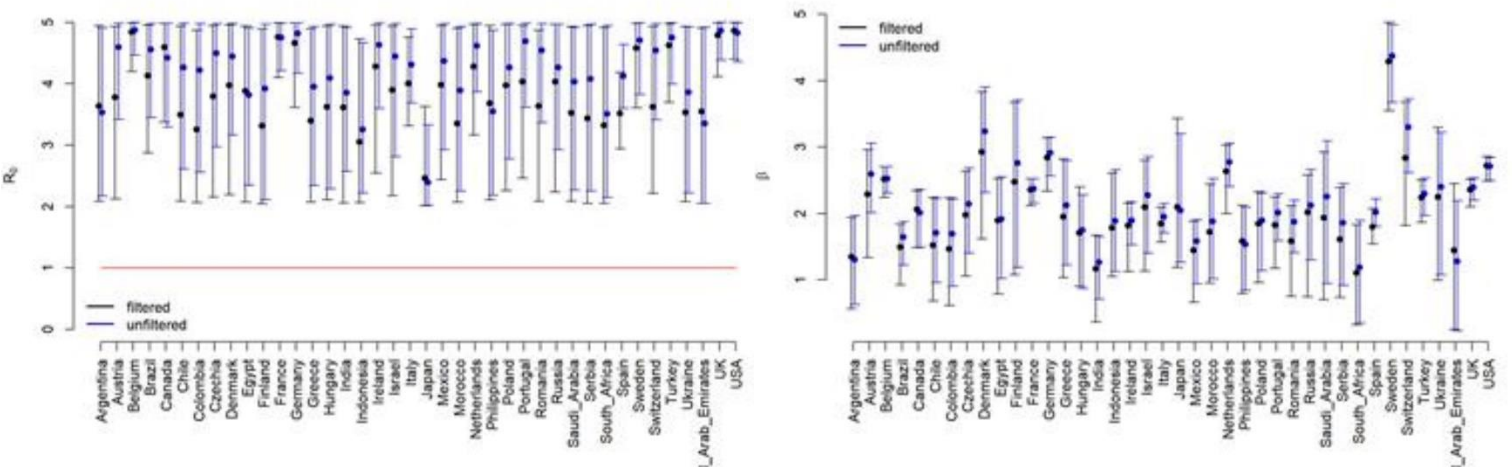

Supplementary Figure 7: Estimated parameters(median and 95%CrI) when excluding the early epidemic phase (black), or including the early epidemic phase (blue) from the likelihood calculation. As we evaluate the relation with the basic reproduction number, this is based on data up to the 10th May 2020.

Supplementary note 7: Sensitivity of estimated mobility thresholds to the serial interval distribution

We found that the estimated mobility thresholds and how those relate to observed mobility are consistent across assumptions of serial interval distribution and likelihood formulation.

Supplementary Table 2: Sensitivity of estimated thresholds to serial interval distribution, equivalent to Table SI.1 but with alternative serial interval (default serial interval: mean of 6.48 days and standard deviation 3.83 days, alternative serial interval: mean 4.8 and standard deviation 2.7 days).

	**First period**				**Second period**			
**country**	R_0	β	Mobility threshold	Adjusted R-squared	R_0	β	Mobility threshold	Adjusted R-squared
**Afghanistan**	0.93 ; 95%CrI [0.78 ; 1.08]	-0.48 ; 95%CrI [-1.31 ; 0.27]	NA	0				
**Argentina**	2.64 ; 95%CrI [1.33 ; 4.34]	1.23 ; 95%CrI [0.05 ; 2.12]	0.02 ; 95%CrI [NA ; NA]	0.51	0.72 ; 95%CrI [0.4 ; 1.2]	-0.95 ; 95%CrI [-2.28 ; 0.18]	NA	0.26
**Australia**	2.66 ; 95%CrI [1.64 ; 4.07]	1.87 ; 95%CrI [0.59 ; 3.1]	0.53 ; 95%CrI [0.39 ; NA]	0.19	2.19 ; 95%CrI [0.16 ; 4.83]	3.75 ; 95%CrI [-7.51 ; 7.25]	NA	0.22
**Austria**	2.46 ; 95%CrI [1.75 ; 3.42]	2.53 ; 95%CrI [1.48 ; 3.59]	0.32 ; 95%CrI [0.2 ; 0.59]	0.84	1.23 ; 95%CrI [0.99 ; 1.48]	1.05 ; 95%CrI [-0.48 ; 2.76]	NA	0.08
**Bangladesh**	2.54 ; 95%CrI [1.37 ; 4.48]	1.21 ; 95%CrI [-0.08 ; 2.48]	NA	0.27	0.97 ; 95%CrI [0.81 ; 1.17]	-0.35 ; 95%CrI [-1.02 ; 0.39]	NA	0.48
**Belgium**	2.63 ; 95%CrI [2.26 ; 3.07]	2.15 ; 95%CrI [1.74 ; 2.55]	0.45 ; 95%CrI [0.4 ; 0.51]	0.98	1.19 ; 95%CrI [1.06 ; 1.35]	1.49 ; 95%CrI [0.76 ; 2.56]	0.12 ; 95%CrI [0.04 ; 0.27]	0.49
**Bolivia**	2.09 ; 95%CrI [0.55 ; 4.66]	0.47 ; 95%CrI [-1.4 ; 1.71]	NA	0.35	0.8 ; 95%CrI [0.54 ; 1.16]	-0.58 ; 95%CrI [-1.35 ; 0.17]	NA	0.32
**Brazil**	2.78 ; 95%CrI [1.9 ; 3.81]	1.45 ; 95%CrI [0.61 ; 2.16]	0.71 ; 95%CrI [0.6 ; NA]	0.87	0.91 ; 95%CrI [0.83 ; 1.01]	-0.23 ; 95%CrI [-0.55 ; 0.12]	NA	0.73

**Canada**	2.9 ; 95%CrI [1.96 ; 4]	1.69 ; 95%CrI [0.89 ; 2.4]	0.63 ; 95%CrI [0.54 ; 0.83]	0.56	1.13 ; 95%CrI [0.97 ; 1.34]	1.06 ; 95%CrI [0.31 ; 2.26]	0.12 ; 95%CrI [-0.08 ; 0.22]	0.2
**Chile**	3.67 ; 95%CrI [1.75 ; 4.91]	1.96 ; 95%CrI [0.47 ; 2.68]	0.66 ; 95%CrI [0.57 ; NA]	0.68	1.35 ; 95%CrI [0.95 ; 1.9]	1.23 ; 95%CrI [0.12 ; 2.42]	0.24 ; 95%CrI [-0.37 ; 0.3]	0.32
**Colombia**	3.45 ; 95%CrI [1.66 ; 4.89]	1.81 ; 95%CrI [0.38 ; 2.6]	0.69 ; 95%CrI [0.56 ; NA]	0.77	0.87 ; 95%CrI [0.73 ; 1.05]	-0.67 ; 95%CrI [-1.22 ; -0.09]	NA	0.56
**Costa_Rica**	2.12 ; 95%CrI [0.74 ; 4.53]	0.41 ; 95%CrI [-1.84 ; 2.29]	NA	-0.01	1.08 ; 95%CrI [0.51 ; 2.29]	-0.11 ; 95%CrI [-2.29 ; 1.94]	NA	0.43
**Czechia**	3.4 ; 95%CrI [2.04 ; 4.87]	2.92 ; 95%CrI [1.49 ; 4.08]	0.5 ; 95%CrI [0.44 ; 0.66]	0.93	3.07 ; 95%CrI [0.98 ; 4.93]	2.08 ; 95%CrI [-1.13 ; 3.44]	NA	0.07
**Denmark**	3.11 ; 95%CrI [1.93 ; 4.5]	3.08 ; 95%CrI [1.49 ; 4.44]	0.37 ; 95%CrI [0.3 ; 0.49]	0.96	1.03 ; 95%CrI [0.76 ; 1.35]	1.41 ; 95%CrI [-0.26 ; 3.19]	NA	0.26
**Dominican_R.**	1.08 ; 95%CrI [0.89 ; 1.29]	0.12 ; 95%CrI [-0.9 ; 1.03]	NA	0.05				
**Ecuador**	0.89 ; 95%CrI [0.78 ; 1.02]	-0.17 ; 95%CrI [-0.89 ; 0.54]	NA	0				
**Egypt**	2.68 ; 95%CrI [1.69 ; 4.08]	1.5 ; 95%CrI [0.59 ; 2.41]	0.65 ; 95%CrI [0.55 ; 0.97]	0.85	1.01 ; 95%CrI [0.88 ; 1.16]	0.44 ; 95%CrI [-0.37 ; 1.38]	NA	0.29
**France**	3.07 ; 95%CrI [2.66 ; 3.58]	1.84 ; 95%CrI [1.58 ; 2.11]	0.61 ; 95%CrI [0.57 ; 0.65]	0.71	1.1 ; 95%CrI [1 ; 1.21]	0.93 ; 95%CrI [0.46 ; 1.51]	0.11 ; 95%CrI [0 ; 0.23]	0.31
**Germany**	2.92 ; 95%CrI [2.29 ; 3.53]	2.78 ; 95%CrI [2.06 ; 3.44]	0.39 ; 95%CrI [0.35 ; 0.44]	0.94	0.99 ; 95%CrI [0.88 ; 1.11]	1.15 ; 95%CrI [0.63 ; 1.77]	-0.01 ; 95%CrI [-0.11 ; 0.1]	0.45
**Guatemala**	0.82 ; 95%CrI [0.65 ; 1.01]	-1.14 ; 95%CrI [-1.85 ; -0.41]	NA	0.41				
**Honduras**	0.99 ; 95%CrI [0.84 ; 1.16]	-0.39 ; 95%CrI [-0.93 ; 0.19]	NA	0.06				
**Hungary**	2.76 ; 95%CrI [1.6 ; 4.37]	1.66 ; 95%CrI [0.51 ; 2.64]	0.61 ; 95%CrI [0.47 ; NA]	0.69	1.54 ; 95%CrI [1.27 ; 1.88]	1.42 ; 95%CrI [0.41 ; 2.66]	0.3 ; 95%CrI [0.18 ; 0.75]	0.26
**India**	2.87 ; 95%CrI [1.76 ; 4.04]	1.27 ; 95%CrI [0.39 ; 1.93]	0.83 ; 95%CrI [0.7 ; NA]	0.65	0.85 ; 95%CrI [0.75 ; 0.96]	-0.57 ; 95%CrI [-0.89 ; -0.24]	NA	0.84

**Indonesia**	2.06 ; 95%CrI [1.44 ; 2.93]	1.11 ; 95%CrI [0.37 ; 1.83]	0.66 ; 95%CrI [0.48 ; NA]	0.44	0.98 ; 95%CrI [0.71 ; 1.39]	-0.24 ; 95%CrI [-1.06 ; 0.73]	NA	0.06
**Iraq**	1.53 ; 95%CrI [0.94 ; 2.34]	0.93 ; 95%CrI [-0.79 ; 2.74]	NA	0.27	0.84 ; 95%CrI [0.73 ; 0.97]	-2.76 ; 95%CrI [-4.1 ; -1.49]	NA	0.86
**Ireland**	3.05 ; 95%CrI [2.19 ; 4.15]	2.24 ; 95%CrI [1.49 ; 2.99]	0.44 ; 95%CrI [0.37 ; 0.54]	0.48	1.53 ; 95%CrI [0.96 ; 2.41]	1.45 ; 95%CrI [0.06 ; 3.09]	NA ; 95%CrI [NA ; NA]	0.14
**Israel**	2.49 ; 95%CrI [1.59 ; 3.93]	1.66 ; 95%CrI [0.62 ; 2.76]	0.55 ; 95%CrI [0.43 ; 0.9]	0.58	1.41 ; 95%CrI [1.14 ; 1.76]	2.63 ; 95%CrI [0.88 ; 4.61]	0.13 ; 95%CrI [0.08 ; 0.23]	0.47
**Italy**	2.16 ; 95%CrI [1.89 ; 2.44]	1.45 ; 95%CrI [1.21 ; 1.71]	0.53 ; 95%CrI [0.49 ; 0.58]	0.92	1.19 ; 95%CrI [1.05 ; 1.35]	0.87 ; 95%CrI [0.34 ; 1.45]	0.21 ; 95%CrI [0.09 ; 0.36]	0.27
**Japan**	1.47 ; 95%CrI [1.11 ; 1.98]	1 ; 95%CrI [0.2 ; 1.83]	0.43 ; 95%CrI [0.22 ; NA]	0.18	1.04 ; 95%CrI [0.79 ; 1.31]	-2.25 ; 95%CrI [-5.87 ; 0.96]	NA	0.48
**Libya**	1.06 ; 95%CrI [0.81 ; 1.36]	-1.03 ; 95%CrI [-2.6 ; 0.64]	NA	0.18				
**Mexico**	0.87 ; 95%CrI [0.8 ; 0.94]	-0.58 ; 95%CrI [-0.82 ; -0.32]	NA	0.04				
**Moldova**	2.32 ; 95%CrI [1.1 ; 4.37]	2.85 ; 95%CrI [-0.98 ; 7.53]	NA	0.5	1.12 ; 95%CrI [0.72 ; 1.84]	0 ; 95%CrI [-3.11 ; 3.78]	NA	0
**Morocco**	2.53 ; 95%CrI [1.73 ; 3.57]	1.81 ; 95%CrI [0.84 ; 2.69]	0.52 ; 95%CrI [0.4 ; 0.75]	0.5	2.16 ; 95%CrI [0.82 ; 4.28]	2.99 ; 95%CrI [-1.58 ; 6.46]	NA	0.66
**Netherlands**	2.61 ; 95%CrI [2.11 ; 3.21]	1.93 ; 95%CrI [1.45 ; 2.4]	0.5 ; 95%CrI [0.45 ; 0.57]	0.93	1.44 ; 95%CrI [1.18 ; 1.77]	1.39 ; 95%CrI [0.57 ; 2.37]	0.26 ; 95%CrI [0.16 ; 0.46]	0.28
**Pakistan**	2.77 ; 95%CrI [1.44 ; 4.61]	1.58 ; 95%CrI [0.13 ; 2.75]	0.65 ; 95%CrI [0.52 ; NA]	0.84	0.85 ; 95%CrI [0.67 ; 1.1]	-1.04 ; 95%CrI [-2.45 ; 0.62]	NA	0.12
**Panama**	2.12 ; 95%CrI [1.26 ; 3.34]	1.41 ; 95%CrI [0.02 ; 2.69]	0.54 ; 95%CrI [0.41 ; NA]	0.4	2.81 ; 95%CrI [0.67 ; 4.91]	3.65 ; 95%CrI [-1.52 ; 5.79]	NA	0.3
**Paraguay**	1.57 ; 95%CrI [0.83 ; 3.57]	1.53 ; 95%CrI [-2.26 ; 6.18]	NA	0				

**Peru**	2.57 ; 95%CrI [1.43 ; 4.33]	0.97 ; 95%CrI [0.08 ; 1.74]	0.97 ; 95%CrI [0.78 ; NA]	0.61	0.68 ; 95%CrI [0.37 ; 1.13]	-0.69 ; 95%CrI [-1.74 ; 0.28]	NA	0.36
**Philippines**	1.29 ; 95%CrI [0.83 ; 2.01]	0.41 ; 95%CrI [-0.36 ; 1.17]	NA	0.41				
**Poland**	1.23 ; 95%CrI [0.88 ; 1.68]	0.2 ; 95%CrI [-0.42 ; 0.82]	NA	0.04	1.37 ; 95%CrI [1.18 ; 1.57]	-1.65 ; 95%CrI [-3.33 ; 0.06]	NA	0.45
**Portugal**	2.59 ; 95%CrI [1.94 ; 3.39]	1.72 ; 95%CrI [1.13 ; 2.31]	0.55 ; 95%CrI [0.46 ; 0.69]	0.92	1.19 ; 95%CrI [1.04 ; 1.36]	0.39 ; 95%CrI [-0.17 ; 0.99]	NA	0.19
**Romania**	3.92 ; 95%CrI [2.36 ; 4.95]	2.28 ; 95%CrI [1.33 ; 2.94]	0.6 ; 95%CrI [0.52 ; 0.72]	0.92	1.11 ; 95%CrI [1 ; 1.21]	0.06 ; 95%CrI [-0.54 ; 0.57]	NA	-0.01
**Russia**	3.68 ; 95%CrI [2.21 ; 4.9]	2.03 ; 95%CrI [0.97 ; 2.74]	0.65 ; 95%CrI [0.55 ; 0.88]	0.89	1.03 ; 95%CrI [0.96 ; 1.11]	0.05 ; 95%CrI [-0.21 ; 0.38]	NA	0.24
**Saudi_Arabia**	3.6 ; 95%CrI [1.59 ; 4.92]	2 ; 95%CrI [0.25 ; 2.98]	0.64 ; 95%CrI [0.49 ; NA]	0.87	0.92 ; 95%CrI [0.76 ; 1.11]	-0.87 ; 95%CrI [-1.9 ; 0.12]	NA	0.64
**South_Africa**	0.84 ; 95%CrI [0.75 ; 0.94]	-0.88 ; 95%CrI [-1.31 ; -0.44]	NA	0.1				
**Spain**	1.75 ; 95%CrI [1.6 ; 1.91]	1.51 ; 95%CrI [1.29 ; 1.75]	0.37 ; 95%CrI [0.33 ; 0.42]	0.75	1.14 ; 95%CrI [1.04 ; 1.25]	1.09 ; 95%CrI [0.48 ; 1.88]	0.13 ; 95%CrI [0.04 ; 0.28]	0.48
**Sweden**	2.7 ; 95%CrI [2.05 ; 3.51]	2.57 ; 95%CrI [1.7 ; 3.4]	0.39 ; 95%CrI [0.34 ; 0.46]	0.83	0.99 ; 95%CrI [0.85 ; 1.15]	0.25 ; 95%CrI [-0.51 ; 1.09]	NA	0.08
**Switzerland**	2.48 ; 95%CrI [1.91 ; 3.12]	3.36 ; 95%CrI [2.26 ; 4.39]	0.27 ; 95%CrI [0.23 ; 0.33]	0.93	1.07 ; 95%CrI [0.79 ; 1.42]	2.19 ; 95%CrI [-0.97 ; 5.82]	NA	0.03
**Turkey**	2.78 ; 95%CrI [2.19 ; 3.46]	1.96 ; 95%CrI [1.43 ; 2.46]	0.52 ; 95%CrI [0.46 ; 0.6]	0.94	1.08 ; 95%CrI [0.98 ; 1.18]	0.28 ; 95%CrI [-0.13 ; 0.67]	NA	0.33
**Ukraine**	2.96 ; 95%CrI [1.64 ; 4.54]	2.21 ; 95%CrI [0.54 ; 3.61]	0.5 ; 95%CrI [0.37 ; NA]	0.81	1.06 ; 95%CrI [0.88 ; 1.24]	0.2 ; 95%CrI [-0.39 ; 0.91]	NA	0
**United_Kingdom**	2.71 ; 95%CrI [2.37 ; 3.07]	2.29 ; 95%CrI [1.92 ; 2.63]	0.44 ; 95%CrI [0.41 ; 0.47]	0.98	1.43 ; 95%CrI [1.21 ; 1.67]	1.98 ; 95%CrI [1.19 ; 2.82]	0.18 ; 95%CrI [0.14 ; 0.23]	0.68
**USA**	2.5 ; 95%CrI [2.18 ; 2.81]	1.75 ; 95%CrI [1.47 ; 2.01]	0.52 ; 95%CrI [0.49 ; 0.57]	0.73	1.02 ; 95%CrI [0.96 ; 1.09]	0.37 ; 95%CrI [0.04 ; 0.72]	0.06 ; 95%CrI [-0.87 ; 0.16]	0.12

Supplementary note 8: Modelling over-dispersion

Over-dispersion using a negative binomial likelihood is typically modelled in two ways[35], which differ in how the variance is linked to the mean:

$$NB1: var(X) = \mu_x + \frac{\mu_x}{\delta_1} = \omega_1 \, \mu_x \quad where \quad \omega_1 = 1 + \frac{1}{\delta_1}$$

$$NB2: var(X) = \mu_x + \frac{\mu_x^2}{\delta_2} = \omega_2 \, \mu_x \quad where \quad \omega_2 = 1 + \frac{\mu_x}{\delta_2}$$

While, arguably, NB2 has somehow more solid statistical foundation with this formulation being formally derived from X following a binomial distribution with heterogeneous μ_x following a gamma distribution, the NB1 is also very popular (generalized linear models) due to its simple interpretation (scaling the Poisson variance).

There are no clear rule on using one formulation or another, and if the range of observed X is not too wide, both formulations will give similar results.

However, when the range of X spans multiple orders of magnitude, the formulation will affect the regression parameter estimates (see Supplementary Figure 8 below).

NB1; $\omega_1 \sim 13$ NB2; $\delta_2 \sim 5$

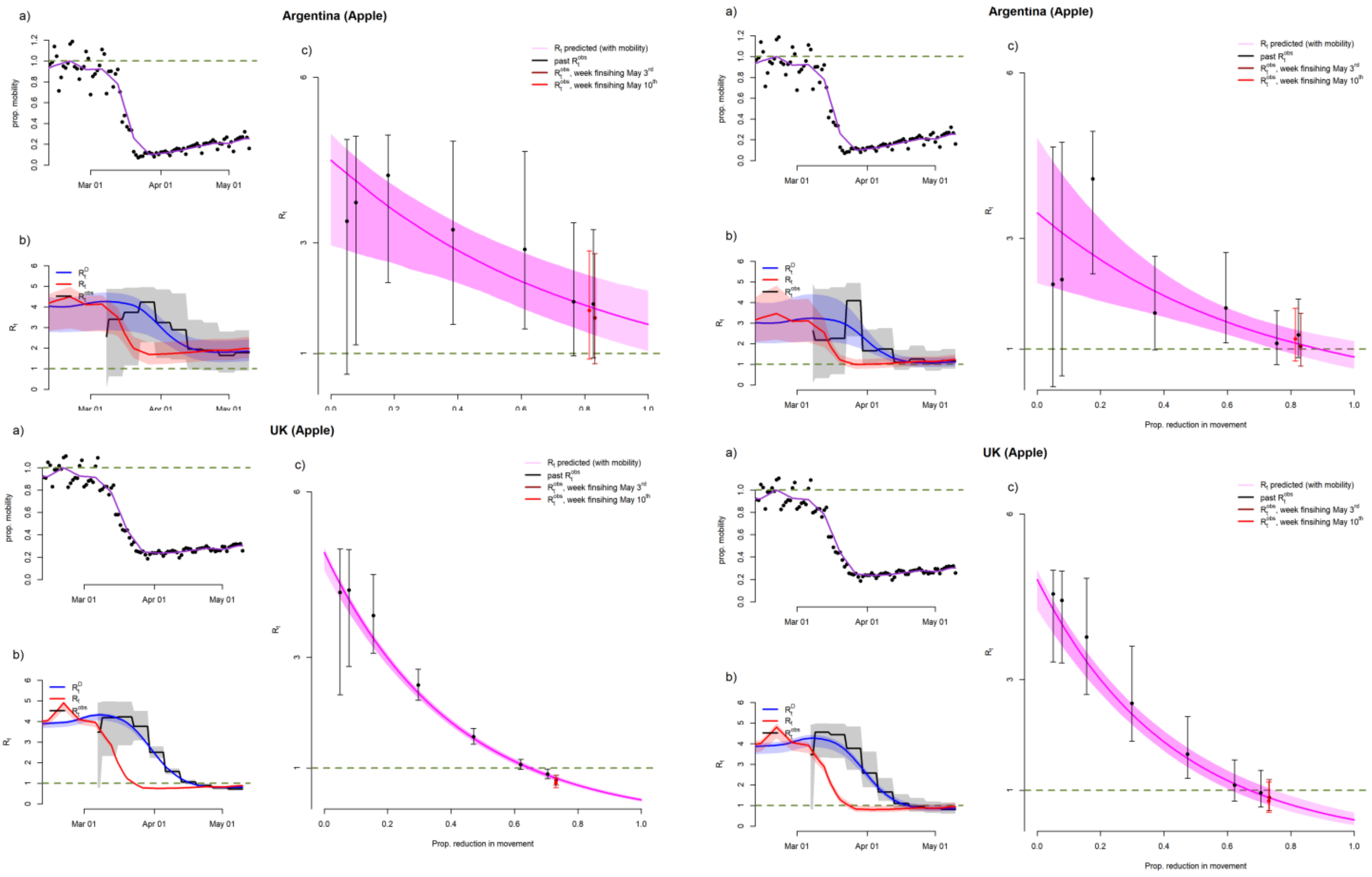

Supplementary Figure 8: Estimated R_t's (dots and lines: medians; error bars and bands: 95%CrI) using NB1 or NB2 for Argentina (low-incidence setting) and the UK (high-incidence setting). Results based on Apple mobility up to the 10th of May 2020.

This in turns has implications in term of forecasts- i.e. projecting forward based on the same relationship and assumption of future mobility patterns (figure below)

NB1; $\omega_1 \sim 13$ NB2; $\delta_2 \sim 5$

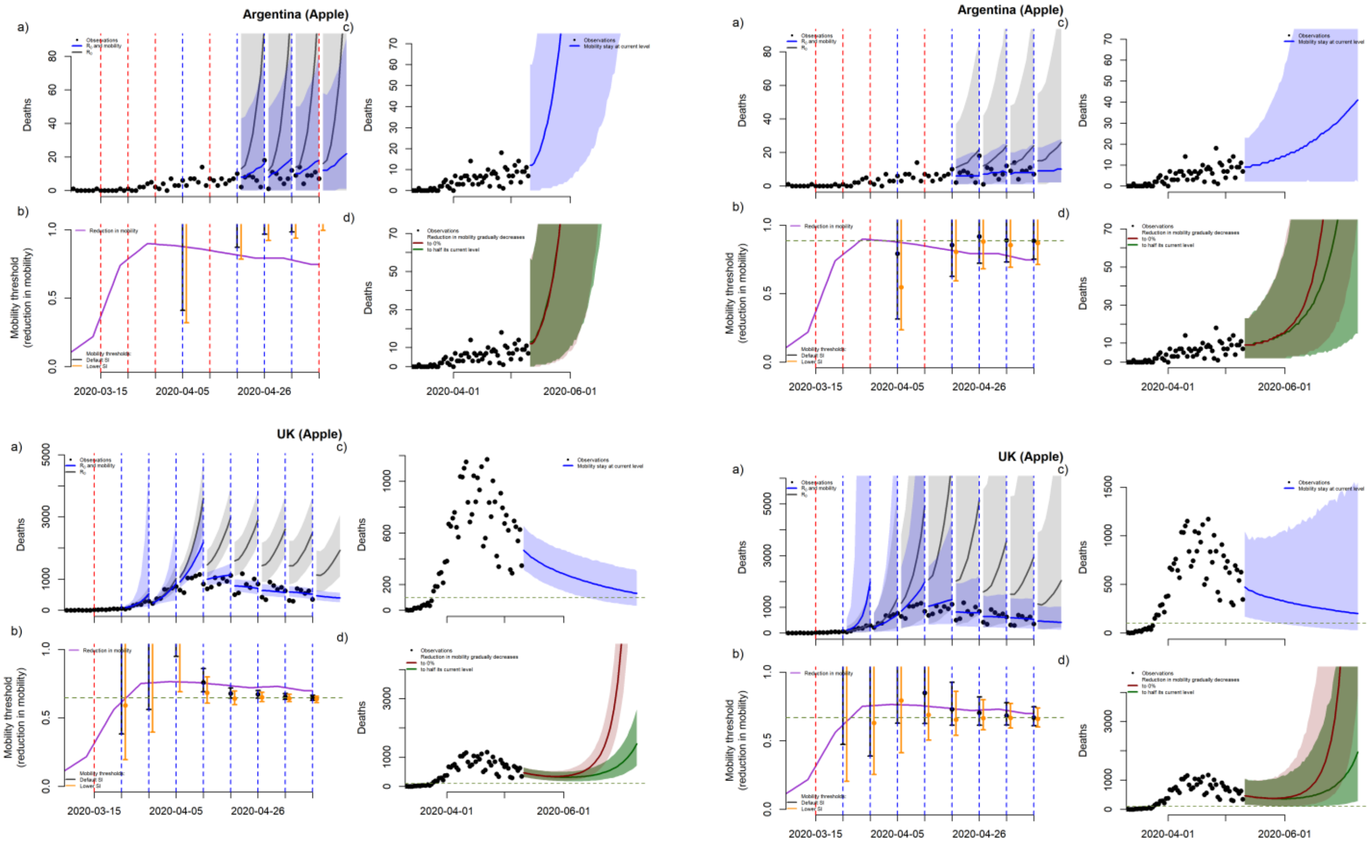

Supplementary Figure 9: Forecasted incidence of deaths using NB1 or NB2 for Argentina (low-incidence setting) and the UK (high-incidence setting). Results based on Apple mobility . Dots and lines: medians; error bars and bands: 95%CrI.

From the estimated R_t's and forecasts, the NB1 structure tends to over-estimate R_t and the variance in forecasts when the incidence is low (i.e. Argentina). The NB2 structure tends to over-estimate the uncertainty in R_t and the variance in forecasts when the incidence is high (i.e. UK).

This suggests that:

- When incidence is low, $var(X)$ is over-estimated with NB1, but reasonable for NB2,
- When incidence is high, $var(X)$ is over-estimated with NB2, but reasonable for NB1.

We therefore propose that instead of 'scaling' the variance by μ_x^2 (NB2) or μ_x (NB1), we use an alternative formation where the increase variance slows down as μ_x increases (Supplementary Table 3):

$$NBsqrt: var(X) = \mu_x + \frac{\mu_x^2}{\delta_{sqrt}\sqrt{\mu_x}} = \omega_{sqrt}\,\mu_x \quad where \quad \omega_{sqrt} = 1 + \frac{\sqrt{\mu_x}}{\delta_{sqrt}}$$

Supplementary Table 3: Factor to multiply the mean to obtain the variance given estimated over-dispersion under alternative NB models.

	NB1	NBsqrt	NB2
$\boldsymbol{X}$ (incidence)	$\omega_1 = 1 + \frac{1}{\delta_1} \sim 13$ With estimated $\delta_1 \sim 0.08$	$\omega_{sqr} = 1 + \frac{\sqrt{\mu_x}}{\delta_{sqrt}}$ With estimated $\delta_{sqrt} \sim 0.8$	$\omega_2 = 1 + \frac{\mu_x}{\delta_2}$ With estimated $\delta_2 \sim 5$
**1**	13	2.3	1.2
**10**	13	5.0	3
**20**	13	6.6	5
**50**	13	9.8	11
**100**	13	13.5	21
**500**	13	29.0	101
**1000**	13	40.5	201

The NBsqrt model outlined seems to better capture the link between variance and mean (Supplementary Figure 10 below). This could reflect the fact that the variance is reflecting 2 processes generating heterogeneity:

- Heterogeneity in transmissibility (i.e. super spreading), which would be linked to NB2, see[36], and would impact more when incidence is small.
- Heterogeneity in reporting, which would be linked to NB1 as the variance would not necessarily dramatically increase as incidence increased.

Based on DIC, we confirmed the improved fit (NB1 DIC = 39,115. NB2 DIC = 36,060, NBsqrt DIC = 35,916).

$NBsqrt; \delta_{sqrt} \sim 0.8$

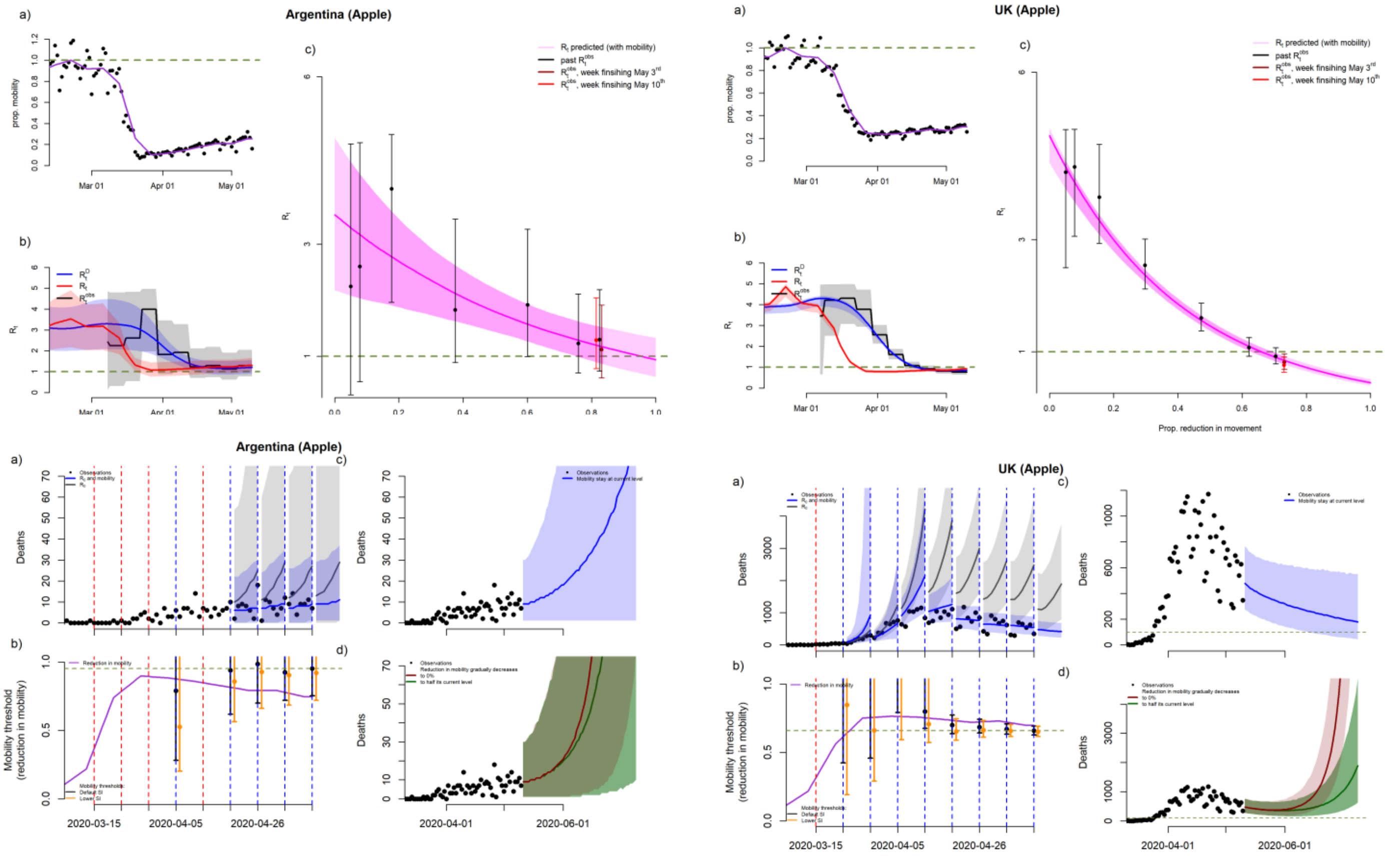

Supplementary Figure 10: Estimated R_t's and forecasted incidence rates of deaths using NBsqrt for Argentina (low-incidence setting) and the UK (high-incidence setting). Results based on Apple mobility. Dots and lines: medians; error bars and bands: 95%CrI.

In the final models, we estimate the median over-dispersion at 0.56 (95%CrI [0.55-0.58]) for the model without time change, and the median over-dispersion at 0.87 (95%CrI [0.74-1.01]) for the model with a time change.

Supplementary note 9: Linking current transmission to transmission measured at deaths

We first characterise incidence of reported deaths:

$$D_{t,i} \sim P\left(R_{t,i}^{D} \sum_{s=0}^{t} \left[D_{s,i}\, w_{t-s} \right] \right)$$

If we do the same for incidence of infections:

$$I_{t,i} \sim P\left(R_{t,i}^{I} \sum_{s=0}^{t} \left[I_{s,i}\, w_{t-s} \right] \right)$$

Relating deaths to infections, we have:

$$R_{t,i}^{D} \sim \frac{D_{t,i}}{\sum_{s=0}^{t}\left[D_{s,i}\, w_{t-s} \right]} = \frac{IFR \sum_{s=0}^{t}\left[I_{s,i}\, h(t-s) \right]}{\sum_{s=0}^{t}\left[D_{s,i}\, w_{t-s} \right]}$$

$$= \frac{\sum_{s=0}^{t}\left[R_{s,i}^{I}\, h(t-s)\left(\sum_{x=0}^{s}\left[I_{x,i}\, w_{s-x} \right] \right) \right]}{\sum_{s=0}^{t}\left[w_{t-s}\left(\sum_{x=0}^{s}\left[I_{x,i}\, h(s-x) \right] \right) \right]}$$

Re-arranging the sum at the bottom, we have:

$$= \frac{\sum_{s=0}^{t}\left[R_{s,i}^{I}\, h(t-s)\left(\sum_{x=0}^{s}\left[I_{x,i}\, w_{s-x} \right] \right) \right]}{\sum_{s=0}^{t}\left[h(t-s)\left(\sum_{x=0}^{s}\left[I_{x,i}\, w_{s-x} \right] \right) \right]}$$

Where $O_s = \sum_{x=0}^{s}\left[I_{x,i}\, w_{s-x} \right]$ is the overall infectivity on day s, so:

$$= \frac{\sum_{s=0}^{t}\left[R_{s,i}^{I}\, h(t-s) O_s \right]}{\sum_{s=0}^{t}\left[h(t-s) O_s \right]}$$

$\sum_{s=0}^{t}\left[h(t-s) O_s \right]$ can be interpreted as an average overall infectivity (weighted by the infection-death delay) at time t: $E[O_t]$, so we have:

$$R_{t,i}^{D} \sim \sum_{s=0}^{t} \left[R_{s,i}^{I}\, h(t-s) \frac{O_s}{E[O_t]} \right]$$

Therefore, when O_s is constant then $R_{t,i}^{D} = \sum_{s=0}^{t}\left[R_{s,i}^{I}\, h(t-s) \right]$, as used in the analysis.

The simplified equation is therefore an approximation. When, epidemic is growing, more weight should be put on recent mobility, so our approximation under-estimates slightly recent changes in mobility. When the epidemic is declining, more weight should be put on past mobility, so our approximation over-estimates slightly recent changes in mobility. Effectively when estimating $R_{t,i}^{D}$ at time t: when R=1, the largest weight is put on $R_{s,i}^{I}$ when s=t-14 days (i.e. 14 days being mode of the h distribution); when R=1.5, the largest weight is put on $R_{s,i}^{I}$ when s=t-11 days; when R=0.8, the largest weight is put on $R_{s,i}^{I}$ when s=t-16 days.

Given uncertainty surrounding transmission, the unknown O_s (we use an estimate of the overall infectivity linked to deaths but not infections), the delay between infection and deaths, and the serial interval, we believe we can confidently use the approximation. The only situation where the approximation would significantly fail would be if we were observing rapid fluctuations (i.e. decrease followed by increase) in mobility with a 2-3 days period. What we observed are smooth changes in mobility, and while the reduction in mobility may be sharp, we never observe a reversion of the mobility reductions on such timescale.

Supplementary note 10: Additional information for the inference

For epidemiological data:

We define a matrix of deaths on day t, for location i:

$$D = \begin{bmatrix} D_{1,1} & \dots & D_{1,i} & \dots & D_{1,n\_loc} \\ D_{2,1} & \dots & D_{2,i} & \cdots & D_{2,n\_loc} \\ D_{3,1} & & & & D_{3,n\_loc} \\ \vdots & & & & \\ D_{n\_days,1} & \dots & D_{n\_days,i} & & D_{n\_days,n\_loc} \end{bmatrix}$$

Overall transmissibility matrix:

$$Ot = \begin{bmatrix} \sum_{s=0}^{1}[D_{s,1}w_{t-s}] & 0 & \\ \sum_{s=0}^{2}[D_{s,1}w_{t-s}] & \sum_{s=0}^{1}[D_{s,2}w_{t-s}] & \dots \\ \sum_{s=0}^{3}[D_{s,1}w_{t-s}] & \sum_{s=0}^{2}[D_{s,2}w_{t-s}] & \\ \vdots & & \dots \\ \sum_{s=0}^{t}[D_{s,1}w_{t-s}] & \dots & \end{bmatrix} = W.D$$

with

$$W = \begin{bmatrix} w_1 & 0 & 0 & \dots & 0 \\ w_2 & w_1 & 0 & \cdots & 0 \\ w_3 & w_2 & w_1 & & 0 \\ \vdots & & & & \\ w_{n\_days} & w_{n_{days}-1} & w_{n_{days}-2} & & w_1 \end{bmatrix}$$

For mobility data:

We define a matrix of deaths reported on day t, for location i:

$$M = \begin{bmatrix} m_{1,1} & \dots & m_{1,i} & \dots & m_{1,n\_loc} \\ m_{2,1} & \dots & m_{2,i} & \cdots & m_{2,n\_loc} \\ m_{3,1} & & & & m_{3,n\_loc} \\ \vdots & & & & \\ m_{n\_days,1} & \dots & m_{n\_days,i} & & m_{n\_days,n\_loc} \end{bmatrix}$$

The mobility at time of death relevant to the time of infection:

$$M^D = \begin{bmatrix} \sum_{s=0}^{1}[m_{s,1}h_{t-s}] & \sum_{s=0}^{1}[m_{s,2}h_{t-s}] & \cdots \\ \sum_{s=0}^{2}[m_{s,1}h_{t-s}] & \sum_{s=0}^{1}[m_{s,2}h_{t-s}] & \\ \sum_{s=0}^{3}[m_{s,1}h_{t-s}] & \sum_{s=0}^{1}[m_{s,2}h] & \\ \vdots & & \cdots \\ \sum_{s=0}^{n\_days}[m_{s,1}h] & \cdots & \end{bmatrix} = H.M$$

with

$$H = \begin{bmatrix} h_1 & 0 & 0 & \dots & 0 \\ h_2 & h_1 & 0 & \cdots & 0 \\ h_3 & h_2 & h_1 & & 0 \\ \vdots & & & & \\ h_{n\_days} & h_{n_{days}-1} & h_{n_{days}-2} & & h_{n_{days}} \end{bmatrix}$$

For the full model:

Given a vector of basic reproduction number and the parameter linking mobility and transmissibility, R_0 , β, the matrix of daily effective reproduction number is:

$$R = \ log(R_0) - B\ (1 - M)$$

where B is a matrix, of size n_days, n_loc ,with each column equal to β.

The reproduction number relevant at the time of death becomes:

$$R^{D,2} = H.R$$

The likelihood is computed from D and $Ot * R^{D,2}$ (with * the element by element product)

For short-term forecasts and longer-term scenarios, we augment the mobility matrix above for future dates, get the effective reproduction matrices (using the joint posterior distribution of estimate R_0 and β), obtain the new augmented matrices of reproduction number at time of death $R^{D,2}$, and finally compute the expected numbers of daily deaths in the future.

Using a Poisson/negative binomial random number generator, we get short-term forecasts or longer-term scenarios.
